# To assemble or not to resemble—A validated Comparative Metatranscriptomics Workflow (CoMW)

**DOI:** 10.1093/gigascience/giz096

**Published:** 2019-07-30

**Authors:** Muhammad Zohaib Anwar, Anders Lanzen, Toke Bang-Andreasen, Carsten Suhr Jacobsen

**Affiliations:** 1 Department of Environmental Science, Aarhus University RISØ Campus, Frederiksborgvej 399, 4000 Roskilde, Denmark; 2 AZTI, Herrera Kaia, Portualdea z/g, 20110 Pasaia, Basque Country, Spain; 3 IKERBASQUE, Basque Foundation for Science, 48011 Bilbao, Spain; 4 Department of Biology, University of Copenhagen, Ole Maaloes Vej 5, 2200 Copenhagen, Denmark

**Keywords:** metatranscriptomics, benchmarking, assembly, alignment, precision, recall, false-positive results

## Abstract

**Background:**

Metatranscriptomics has been used widely for investigation and quantification of microbial communities’ activity in response to external stimuli. By assessing the genes expressed, metatranscriptomics provides an understanding of the interactions between different major functional guilds and the environment. Here, we present a *de novo* assembly-based Comparative Metatranscriptomics Workflow (CoMW) implemented in a modular, reproducible structure. Metatranscriptomics typically uses short sequence reads, which can either be directly aligned to external reference databases (“assembly-free approach”) or first assembled into contigs before alignment (“assembly-based approach”). We also compare CoMW (assembly-based implementation) with an assembly-free alternative workflow, using simulated and real-world metatranscriptomes from Arctic and temperate terrestrial environments. We evaluate their accuracy in precision and recall using generic and specialized hierarchical protein databases.

**Results:**

CoMW provided significantly fewer false-positive results, resulting in more precise identification and quantification of functional genes in metatranscriptomes. Using the comprehensive database M5nr, the assembly-based approach identified genes with only 0.6% false-positive results at thresholds ranging from inclusive to stringent compared with the assembly-free approach, which yielded up to 15% false-positive results. Using specialized databases (carbohydrate-active enzyme and nitrogen cycle), the assembly-based approach identified and quantified genes with 3–5 times fewer false-positive results. We also evaluated the impact of both approaches on real-world datasets.

**Conclusions:**

We present an open source *de novo* assembly-based CoMW. Our benchmarking findings support assembling short reads into contigs before alignment to a reference database because this provides higher precision and minimizes false-positive results.

## Introduction

Metatranscriptomics provides an unprecedented insight to complex functional dynamics of microbial communities in various environments. The method has been applied to study the microbial activity in thawing permafrost and the related biogeochemical mechanisms contributing to greenhouse gas emissions [1], and Gonzalez et al. [2] applied metatranscriptomics to evaluate root microbiome response to soil contamination. Metatranscriptomics has also been used to study the functional human gut microbiota [3, 4]. The method is typically used to identify, quantify, and compare the functional response of microbial communities in natural habitats or in relation to environmental or physio-chemical impacts.

Using high-throughput sequencing techniques such as Illumina, metatranscriptomics offers a non-PCR–biased method for looking at transcriptional activity occurring within a complex and diverse microbial population at a specific point in time [5]. However, curation and annotation of these complex data has emerged as a major challenge. To date, several studies have used various analytic workflows. Typically, short sequence reads are used, which can be either individually aligned directly to external reference databases (hereafter “assembly-free”) or assembled into longer contiguous fragments (contigs) for alignment (hereafter “assembly-based”). Various studies have used either of these 2 general approaches. For example, Poulsen et al. [6] used an assembly-based approach. An open-source pipeline, IMP [7] also uses this approach in integrated metagenomic and metatranscriptomic analyses. The assembly-free approach has instead been used by, e.g., Jung et al. [8], aligning short reads to reference genomes of lactic acid bacterial strains associated with the kimchi microbial community. Similarly, an open source pipeline developed by Martinez et al. [9] to analyse metatranscriptomics datasets also aligns short reads directly to a protein database before annotation. The choice of either of these 2 alternatives for metatranscriptomics analyses may depend on lack of thorough comparisons. Because no independent and direct comparison between them has been performed, various metatranscriptomics analysis approaches may at times produce inconsistent observations, even if identical databases are used in the analysis. Thus, standardization of computational analysis is necessary to enable further propagation of metatranscriptomics approaches and their integration into microbial ecology research. Benchmarking provides a critical view of the efficiency and precision of different workflows, and use of simulated communities for benchmarking enables the analysis to be independent of experimental variation and biases [10].

Here, we present the Comparative Metatranscriptomic Workflow (CoMW) implemented using the *de novo* assembly-based approach, standardized and validated for functional annotation and quantitative expression analysis. We validated the suitability of CoMW for functional analysis by comparing it with a typical assembly-free approach using simulated datasets and evaluated the accuracy of both approaches using precision, recall, and false discovery rates (FDRs). Three different protein databases were selected for this benchmarking in order to include a representative selection of 3 different degrees of specialization, on a range from a more inclusive database with wide coverage (universality) and low degree of expert curation to a smaller, highly curated database, with more narrow coverage: (i) M5nr [11], an inclusive and comprehensive non-redundant protein database in combination with Evolutionary Genealogy of Genes: Non-supervised Orthologous Groups (eggNOG) hierarchical annotation; (ii) Carbohydrate-Active Enzymes (CAZy) [12], a database dedicated to describing the families of structurally related catalytic and carbohydrate-binding modules of enzymes; and (iii) Nitrogen Cycling Database (NCycDB) [13], a specialized and manually curated database covering only nitrogen cycle genes. Finally, to estimate the consistency and variance in the results caused by the choice of approach, we then applied them to real-world metatranscriptomes from microbial communities in (i) active-layer permafrost soil from Svalbard, Norway [14], and (ii) ash-impacted Danish forest soil [15].

## Findings

### Comparative Metatranscriptomics Workflow

We have standardized, implemented, and validated a metatranscriptomic workflow (CoMW) using a *de novo* assembly-based approach that can assist in analysing large metatranscriptomics data. It makes each step of the metatranscriptomic workflow straightforward and helps to make these complex analyses more reproducible and the components re-useable in different contexts. The core processes such as open reading frame (ORF) detection and alignment against the functional database are vital in any metatranscriptomic analyses and are, therefore, present uniformly in all workflows. However, because most of the tools performing these core processes are ever improving, the workflow is implemented in modular format to provide the possibility of using alternative tools and databases if preferred or a newer version of these tools. Modularity additionally provides choice—optional steps can be skipped, changed, or even improved in a structural manner; e.g., the scripts are designed to cater contigs from >1 assembler. In addition to core process CoMW has a couple of optional steps such as abundance-based and non-coding RNA filtering, which can be different in datasets from a different environment. CoMW is an open source workflow written in Python available at GitHub [16] and published as a computational capsule on codeocean [17]. An Anaconda cloud environment is created with the provided configuration file to install third-party tools and dependencies. Help regarding input, output, and parameters is provided with each script, and a comprehensive tutorial is presented in the GitHub repository.

### Evaluation of CoMW (assembly-based approach) and comparison with an assembly-free method

**Figure 1:**
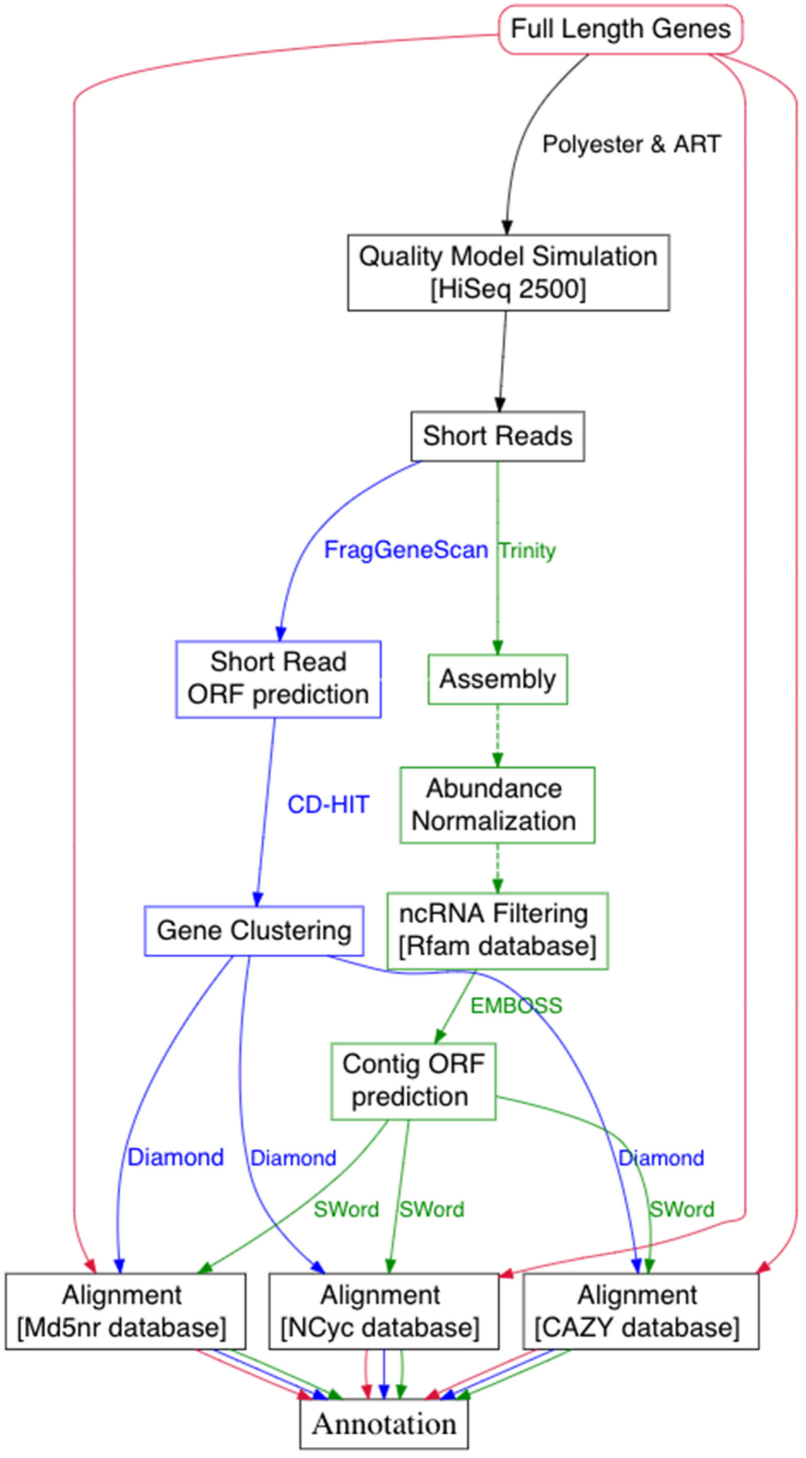
Flow chart illustrating the evaluation and benchmarking scheme used for the comparison of alternative approaches. Red path indicates the full-length genes workflow, green indicates the steps in the assembly-based workflow CoMW, and blue indicates the steps in the assembly-free approach.

To compare the performance of the assembly-based workflow CoMW and assembly-free approaches, we simulated community transcript data using 4,943 full-length genes provided by Martinez et al. [9]. We analysed both approaches separately and compared against direct annotation of full-length genes. The full-length genes were annotated using all 3 databases (M5nr, CAZy, and NCycDB) independently to classify them into functional subsystems and gene families. Fig. 1 shows a detailed workflow of comparative analysis using both approaches.

### Functional assignment

#### M5nr alignment

Full-length genes of the simulated community dataset were aligned and identified into 671 unique eggNOG orthologs, belonging to 19 distinct functional subsystems (Level II). At the default confidence threshold (bit score 50), the assembly-free approach produced alignments to 820 orthologs with a precision of 85% (14.9% false-positive results [FPs]), whereas CoMW identified 665 orthologs with a precision of 99.3% (0.6% FPs) at the default confidence threshold of 1E−5. Repeating the alignments using a gradient of 15 varying confidence thresholds for each approach (low [T_L_], medium [T_M_], and high [T_H_]; 5 thresholds/category) resulted in dissimilar performance for the 2 approaches. The precision and recall of CoMW did not decrease below 99.3% and 98.5%, respectively, throughout all categories whereas the assembly-free approach had a maximum precision of 96.3% at T_M_ and decreased to 85% at T_L_ and T_H_. CoMW also produced fewer (only 0.6%) FPs consistently compared to the assembly-free approach, in which FPs ranged from 14.9% to a minimum of 3.6% at highest precision. Based on F-score the most optimal alignment for each approach is given in Table 1, whereas detailed values for precision, recall, F-score, and FDR are listed in Supplementary Table S1. We then also evaluated both approaches by selectively removing sequences belonging to a certain functional subsystem from the M5nr database in a controlled manner (segmented cross-validation) in order to replicate real-world metatranscriptomes where a certain functional subsystem can be completely or partially absent from the reference database. We removed 4 (Level II) subsystems (“[D] Cell cycle control, cell division, chromosome partitioning”; “[L] Replication, recombination, and repair”; “[E] Amino acid transport and metabolism”; and “[R] General function prediction only” and “[S] Function unknown”). The Level II subsystems were randomly removed (see data availability for the script used for the removal) one at a time, realigning full-length genes and simulated reads using both CoMW and assembly-free approaches to the cropped database to compare identification consistency. In each validation round, both precision and recall of CoMW were significantly higher than with the assembly-free approach. The recall ability of the assembly-free approach decreased significantly in this validation as compared to the full database comparison. CoMW also produced fewer FPs as compared to the assembly-free approach. Table 2 provides details for each validation cycle.

**Table 1:** Comparison of precision, recall, F-score, and FDR for the assembly-free and the CoMW (assembly-based) approaches using all 3 databases based on best F-score

Database	Approach	Threshold	Threshold category	Recall	Precision	F-score	FDR (%)
eggNOG	Assembly-free	BTS 120	Strict (T_H_)	**0.9880**	0.9540	0.9707	4.5977
	CoMW	1.00E−15	Strict (T_H_)	0.9851	**0.9939**	**0.9895**	**0.6006**
CAZy	Assembly-free	BTS 110	Strict (T_H_)	0.3510	0.5325	0.4231	46.7433
	CoMW	1.00E−08	Medium (T_M_)	**0.8131**	**0.7759**	**0.7940**	**22.4096**
NCycDB	Assembly-free	BTS 150	Strict (T_H_)	0.1666	0.0581	0.0862	94.1860
	CoMW	1.00E−14	Strict (T_H_)	**0.6666**	**0.8333**	**0.7407**	**16.6666**

Full table for both approaches and databases can be seen in Tables S1–S3. Boldface emphasizes better precision, recall, F-score, and FDR in each database between both approaches.

**Table 2:** Comparison of precision, recall, F-score, and FDR for the assembly-free and CoMW (assembly-based) approaches using the selective removal of functional subsystems from eggNOG database (segmented cross-validation) to evaluate the consistency of both approaches

Removed subsystem	Approach	Recall	Precision	F-score	FDR (%)
Cell wall/membrane/envelope biogenesis [M]	Assembly-free	0.8726	0.9580	0.9133	4.1958
	CoMW	**0.9792**	**0.9855**	**0.9824**	**1.4423**
Replication, recombination, and repair [L]	Assembly-free	0.8734	0.9588	0.9141	4.1166
	CoMW	**0.9796**	**0.9858**	**0.9827**	**1.415**
Amino acid transport and metabolism [E]	Assembly-free	0.8750	0.9589	0.9150	4.1095
	CoMW	**0.9812**	**0.9874**	**0.9843**	**1.2578**
General function prediction only and Function unknown [R], [S]	Assembly-free	0.8933	0.9281	0.9104	7.1856
	CoMW	**0.9884**	**0.97443**	**0.9814**	**2.5568**

Boldface emphasizes better consistency compared with full-length genes.

#### CAZy alignment

From 2,395 full-length genes, 500 sequences were aligned to 395 unique functional genes in the CAZy database, which belonged to 130 gene families and were further classified as 7 enzyme classes. Using default confidence thresholds (BTS 50, 1E−5), the assembly-free approach identified 765 functional genes belonging to 112 unique families and 6 enzyme classes with a precision of 28.5% (71.4% FPs). CoMW identified 488 functional genes from the CAZy database that were classified into 147 gene families from 7 enzyme classes with a precision of 66.0% (FDR 33.9%) at the default confidence threshold. However, when we repeated the process with 15 various confidence thresholds, precision improved consistently and FPs decreased, whereas for the assembly-free approach, precision decreased significantly with increasing confidence threshold (see Table 1 and Supplementary Table S2).

#### NCycDB alignment

A total of 410 of the 2,395 full-length genes were aligned to this database, identified as 29 unique nitrogen cycle genes and further belonging to 15 functional gene families in 5 pathways. Using default confidence thresholds, the assembly-free approach identified 1,541 functional genes belonging to 25 functional gene families classified into 6 pathways with a precision of 0.9% (99.0% FPs). CoMW identified 42 nitrogen cycle genes classified into 25 gene families from 6 pathways with a precision of 59.5% (40.4% FPs) at a default confidence threshold of 1E−5. As with the comparisons against M5nr and CAZy we repeated the process with 15 different confidence thresholds for each approach. Precision improved significantly for CoMW at stringent thresholds whereas for the assembly-free approach, the best precision achieved was 5.8% (Table   1, Supplementary Table S3).

### Expression quantification

We also compared the ability of both approaches to quantify the expression of identified transcripts by performing differential expression analysis of 2 groups in simulated communities and compared against the full-length gene expression simulated. We selected the 3 best identification thresholds for both approaches based on highest F-score and performed differential expression analysis. This analysis for both approaches was carried out against all 3 databases using the most specific level of hierarchy in the respective databases in order to capture their ability to quantify expression levels of specific genes.

**Figure 2:**
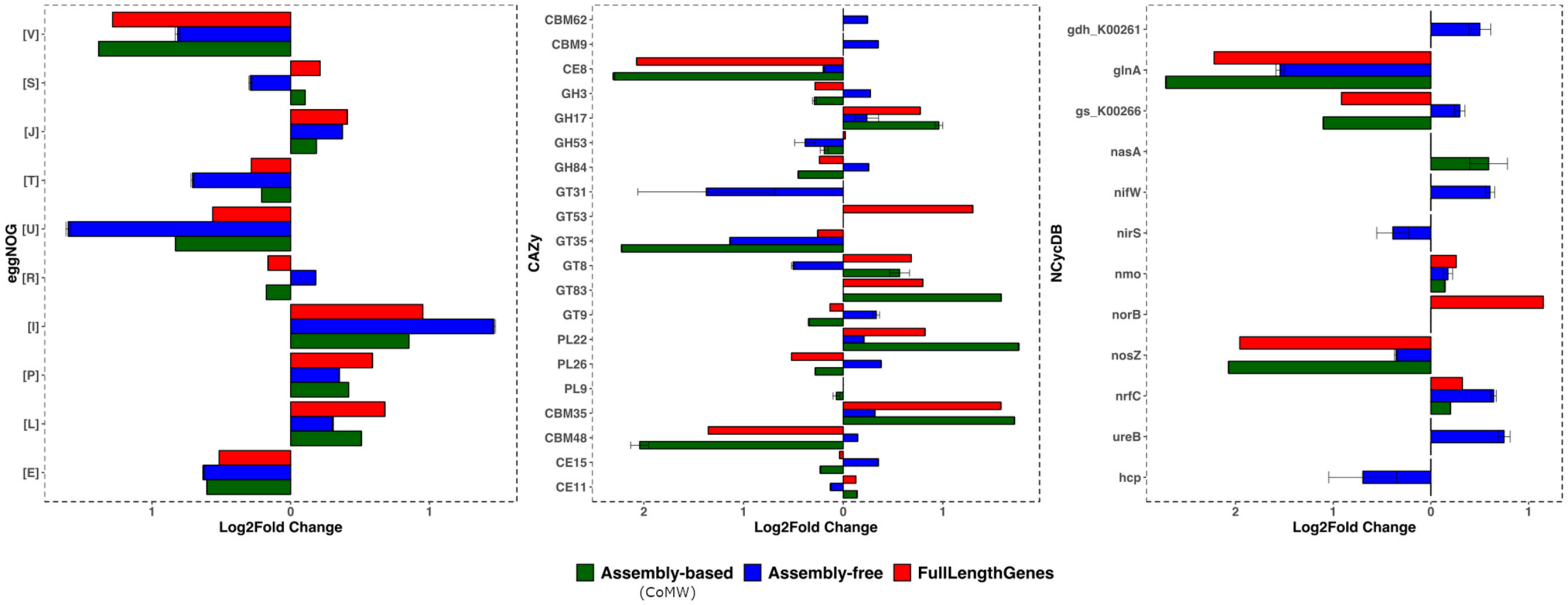
Differential expression comparison of the assembly-free and the CoMW assembly-based approaches using (A) eggNOG database, (B) CAZy, and (C) NCycDB database.

According to full-length gene alignments against eggNOG, 123 genes were significantly upregulated and 270 were significantly downregulated. According to the assembly-free approach (with the best resulting F-score), 73 genes were upregulated (precision 94.5%, 5.4% FPs) and 380 (precision 65.7%, 34.2% FPs) were downregulated, whereas using the assembly-based approach (CoMW), 99 genes were identified as upregulated (precision 94.9%, 5.1% FPs) and 249 downregulated (precision 97.1%, 2.8% FPs). For the CAZy database full-length genes, 81 and 189 genes were identified as significantly up- and downregulated, respectively. Using the assembly-free approach 31 upregulated (precision 19.3%, 80.6% FPs) and 137 downregulated genes (precision 52.5%, 47.4% FPs) were identified, whereas the CoMW identified 83 (precision 71.2%, 28.9% FPs) and 191 (precision 73.8%, 26.1% FPs), respectively. In the NCycDB expression analysis, 3 and 14 genes were seen as significantly up- and downregulated, respectively, using full-length genes. According to the assembly-free approach, 26 (precision 0%, 100% FPs) and 107 (precision 4.6%, 95.3% FPs) genes were up- and downregulated, respectively, whereas according to CoMW, 3 (precision 33.3%, 66.6% FPs) genes were upregulated and 18 (precision 55.5%, 44.4% FPs) were downregulated. Precision, recall, and FDR for both approaches against all 3 databases are available in Supplementary Table S4. Additionally, we collapsed the functional genes into functional subsystems and gene families to remove FPs produced due to identification of homologous proteins or proteins with multiple inheritance. Fold change (log_2_ transformed) was then calculated for each subsystem/gene family (see Fig. 2).

#### Real-world metatranscriptomes

**Figure 3:**
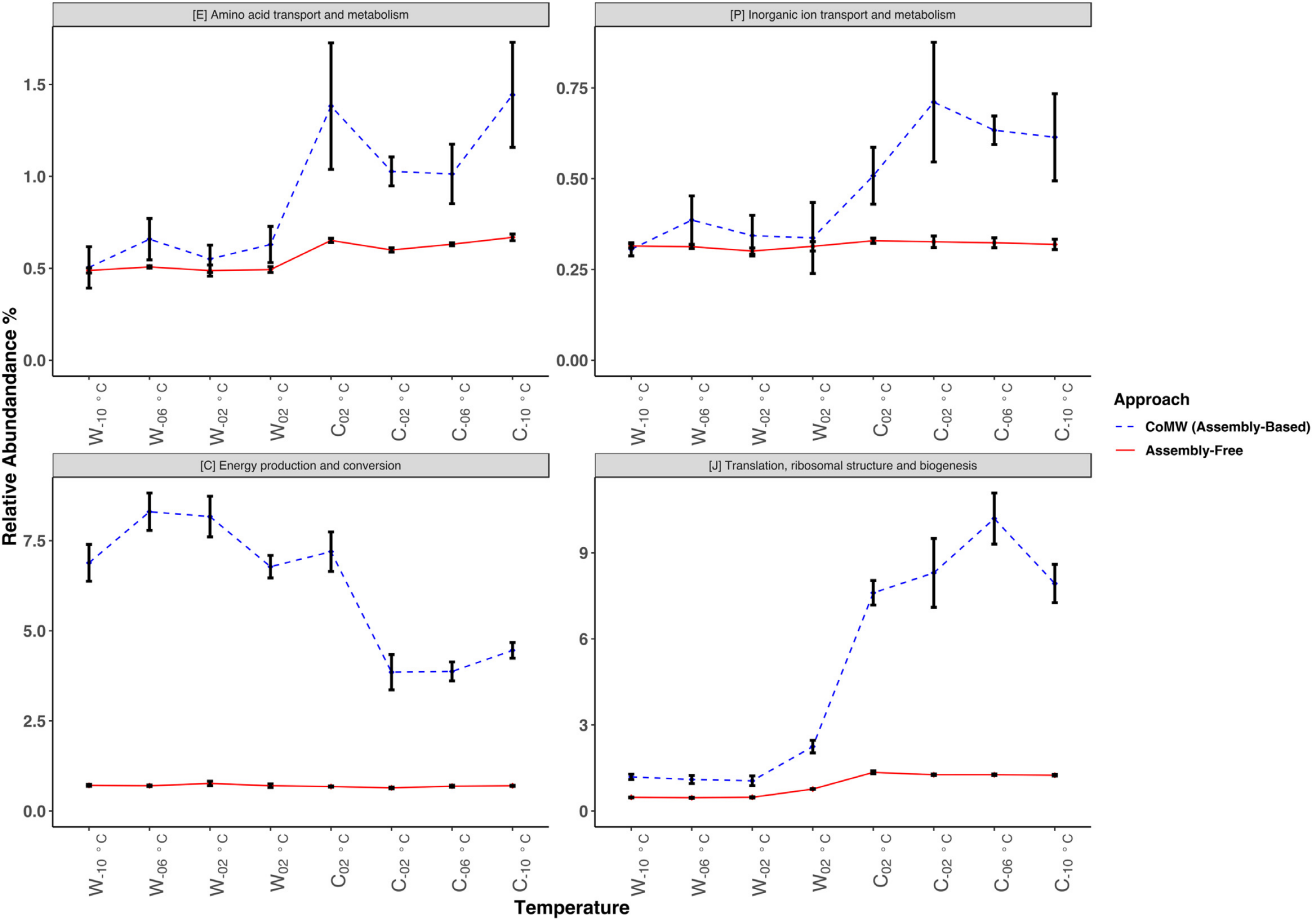
Relative abundance of eggNOG functional subsystems in Arctic permafrost soil identified and quantified using both CoMW and the assembly-free approach compares the differences in observed functional dynamics. Blue dotted line represents trends using CoMW (assembly-based) whereas red solid line represents the assembly-free approach.

To evaluate the effect of the 2 approaches on real-world data, 2 metatranscriptomes from microbial communities were studied. In the first study we investigated the transcriptional response during warming from −10°C to 2°C and subsequent cooling from 2°C to −10°C of an Arctic tundra active layer soil from Svalbard, Norway. The aim of the study was to understand taxonomic and functional shifts in microbial communities caused by thawing and freezing of Arctic soil. A pronounced shift during the incubation period was noticed by Schostag et al. [14] that was not replicated by the assembly-free approach. However, using CoMW, we identified an increase of genes in the subsystem “[P] Inorganic ion transport and metabolism.” During cooling, CoMW also captured the upregulation and downregulation of genes related to “[J] Translation, ribosomal structure, and biogenesis” and “[C] Energy production and conversion,” respectively (Fig. 3), unlike the assembly-free approach. These findings may have implications for our understanding of carbon dioxide emission, nitrogen cycling, and plant nutrient availability in Arctic soils.

**Figure 4:**
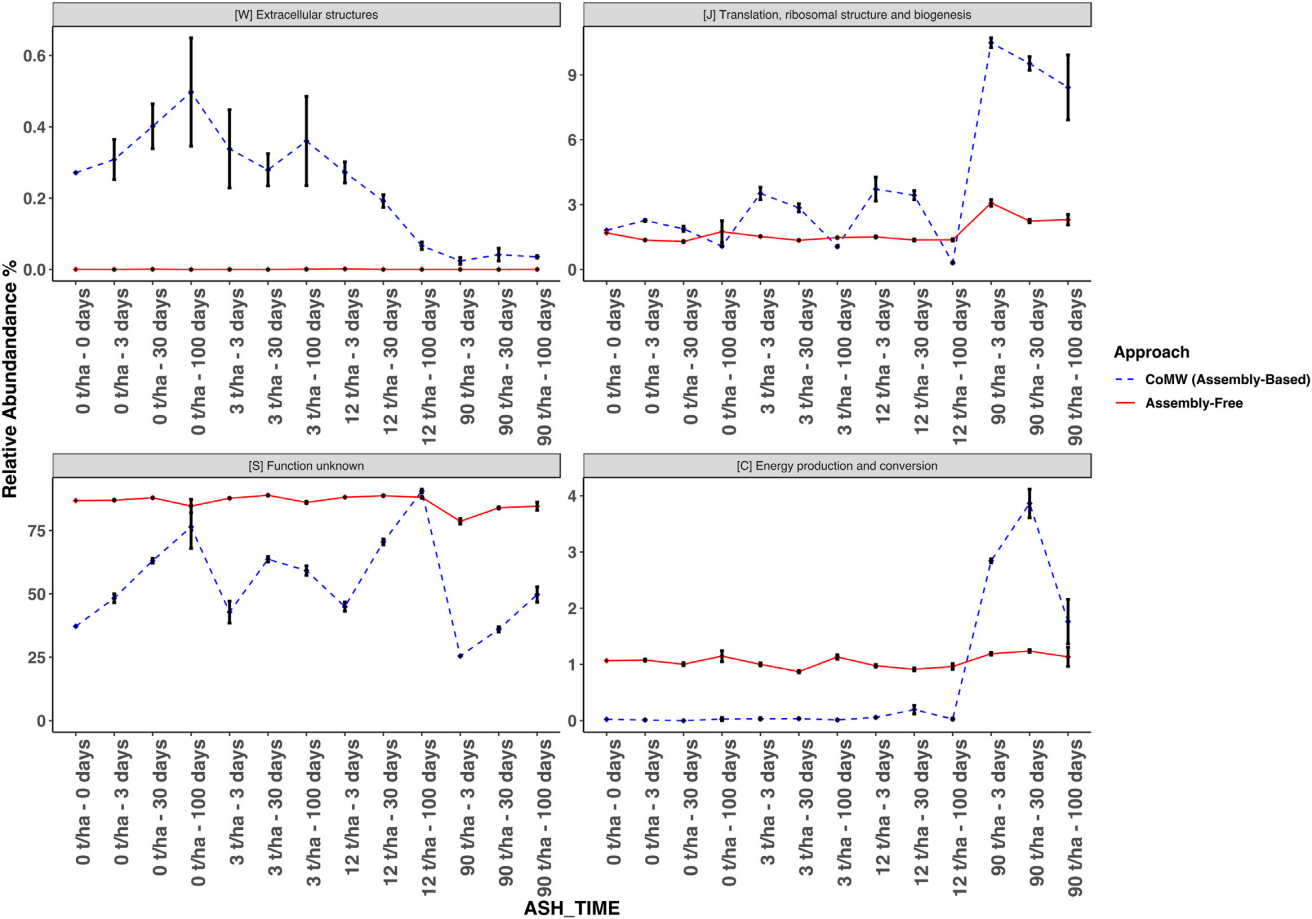
Relative abundance of eggNOG functional subsystems in ash-deposited Danish forest soil with time identified using both the CoMW and an assembly-free approach. Blue dotted line represents trends using CoMW (assembly-based) whereas red solid line represents the assembly-free approach.

In the second study, we investigated the effects of wood ash amendment on Danish forest soils [15]. Ash was added in 3 different quantities (0/control, 3, 12, and 90 tonnes ash per hectare [t ha^−1^]) and the effect over time was analysed in soil communities at 0, 3, 30, and 100 days after ash addition. This resulted in strong effects on functional expression as seen in Fig. 4. Both approaches once again displayed varying results such as changes in genes related to eggNOG functional subsystem “[W] Extracellular structures.” The assembly-free approach also identified 75% of genes as “[S] Function unknown” consistently, unlike the assembly-based approach.

## Discussion

The application of metatranscriptomics is less common than other DNA-based genomics techniques, and thus most analysis pipelines are built ad hoc [18]. An assembly-free approach is used in a few pipelines/workflows such as COMAN [19], Metatrans [9], and SAMSA2 [20], while an assembly-based approach is used in a few pipelines as well such as IMP [7]. The lack of thorough benchmarking studies and standardized workflows in metatranscriptomics has made it a more challenging task to analyse the typically big datasets produced. Previous studies, e.g., Zhao et al. [21] and Celaj et al. [22], have compared *de novo* sequence assemblers including Trinity [23], MetaVelvet [24], Oases [25], ABySS [26], and SOAPdenovo [27]. Similarly, for the assembly-free approach direct short-read mappers have been compared thoroughly such as DIAMOND [28], BLASTX [29], and RAPSearch2 [30], but an independent comparison of the 2 different approaches based on including assembly or directly aligning reads (here “assembly-free”) has been lacking. Critical Assessment of Metagenomic Interpreter (CAMI) [31] is so far the most comprehensive benchmarking effort; however, it lacks any similar metatranscriptomics benchmarking. IMP [7] uses an integrated approach of metagenomics and metatranscriptomics and has some overlapping areas to CoMW and can be used together owing to the modular approach of CoMW.

Using simulated samples composed of genes collected from abundant genomes provided by Martinez et al. [9], we show that both approaches provide similarly high recall rates against the general comprehensive database M5nr. However, CoMW provided a significantly better precision and a lower FDR for identification and quantification. For relatively compact and specialized databases, recall and precision decrease for both approaches (especially for the most compact database NCycDB); whereas CoMW still seemed to be more precise, meaning that fewer genes were misassigned against these databases and significantly fewer FPs were produced.

We have attempted to assist this decision making for processing metatranscriptomic analysis by independently assessing the performance of the 2 most common approaches and provide a road map for functional annotation and expression quantification against databases ranging from inclusive to specialized. The significantly higher precision in identification and quantification for gene families and functional subsystems in simulated samples, against all 3 databases, confirmed that while an assembly step is challenging computationally, it holds the potential to reveal information regarding gene expression that is not attainable without it. Selecting a single best workflow or pipeline for all types of metatranscriptomics studies is not a straightforward affair, and we believe that choice of approach changes the outcome of study significantly as observed with real-world datasets from active-layer permafrost soil from Svalbard, Norway, and ash-impacted Danish forest soil. In addition to choosing the right workflow, combining that with the appropriate reference database is equally important to ensure the best annotation performance. With databases specialized for ≥1 specific environments or functional categories, the assembly-free approach underperforms owing to its inability to identify alignments to homologues in the reference database. We also show that the assembly-free approach can increase the FDR in annotation when a database is dominant in specific functional subsystem, which can also lead to wrong estimation of fold change in expression.

While taxonomic annotation is beyond the scope of CoMW and thus our benchmarking analyses, it is important to consider the limited value of most functional genes for and thus functional metatranscriptomics alone for structural profiling of environmental communities, due to the high rate of horizontal gene transfer [32]. Approaches for this purpose include the identification of a limited set of “phylogenetic marker genes” (e.g., [33]) or “total RNA” metatranscriptomics whereby the ribosomal RNA content is retained and used for taxonomic analysis [34]. Although not shown here, we expect that the former approach would also benefit in accuracy from assembling messenger RNA to full-length transcripts before classification, based on our results regarding functional diversity. The total RNA approach also benefits from custom ribosomal RNA targeted assembly [15], which may be incorporated into CoMW thanks to its modularity.

In summary, we present the assembly-based workflow CoMW and show that this approach results in consistently better accuracy for functional analysis of metatranscriptomics data. Our benchmarking results show that the choice of approach (assembly-free vs assembly-based) and database significantly affects the quality of the identification, annotation, and expression results. Given the impact of each of these variables, it is inevitable that it significantly affects the results of an individual study and comparison across studies. We believe that the work presented here will both provide a useful tool for and assist the microbial ecology research community to make more informed decisions about the most appropriate methodological approach to analyse large metatranscriptomic datasets with improved precision.

## Methods

### CoMW implementation

CoMW (assembly-based) is based on 4 major steps: (i) *de novo* assembly and mapping, (ii) filtering, (iii) gene prediction and alignment, and (iv) annotation.

*De novo* assembly and mapping of short reads back to assembled contigs is done using Trinity [23] and BWA [35], respectively. Various tools have been developed for *de novo*metatranscriptome reconstruction that usually rely on graph theory. Trinity, however, generates the most optimal assemblies for coding RNA reads [18, 22, 36]. Nevertheless, in CoMW, the user can assemble short reads into contigs by any assembler preferred but it can reduce the quality of the following steps such as alignment of contigs.

Filtering of contigs is done to remove variance in sequences/samples. Because CoMW is assembly-based, after we assemble the reads into longer contigs we also propose a 2-step filtering of the contigs to remove any chimeric or false contig made as a result of assembly or sequencing error by removing contigs that have an expression level less than a specific threshold and to remove any potential non-coding RNA contigs assembled. We can filter contig abundance data by removing all contigs with relative expression lower than a specific cut-off, e.g., 1% (selected on the basis of dataset variance) of the number of sequences in the dataset with the fewest sequences. This threshold is also flexible for different datasets and in some cases not required at all, so CoMW allows the user to bypass this step or change the threshold up and down on the basis of data variation. The filtered contigs are subject to potential non-coding RNA filtration by aligning them against the RFam database [37] using infernal [38], which is a secondary-structure–aware aligner that predicts the secondary structure of RNA sequences and similarities based on the consensus structure models. Once again, the non-coding RNA filtering is an optional step in CoMW, although highly recommended in order to reduce FPs.

Gene prediction and alignment is done using Transeq from EMBOSS [39] to predict probable ORFs of the contigs (customizable, by default 6 per contig). We used SWORD [40] as alignment tool against reference databases. SWORD can be used in parallel based on computational resources available, and the aligned results are parsed and cut off at a specific confidence threshold of combination of E-value and alignment length (usually 1E−5, can be changed given the assembly distribution in datasets).

Annotation of aligned transcripts from the previous step can be done using the databases such as eggNOG, which is a hierarchically structured annotation using a graph-based unsupervised clustering available algorithm to produce genome-wide orthology inferences; CAZy, which is a knowledge-based resource specializing in glycogenomics; and NCycDB, a nitrogen cycle database. Aligned proteins are then placed into functional subsystems or gene families based on their best hits. This results in a count table with a contig and eggNOG ortholog or CAZy gene or NCyc gene having a certain count from each sample depending on database used. This count table can be then used for differential expression using a state-of-the-art expression analysis suite such as DESeq2 [41] or its wrapper SARTools [42]. For evaluation of CoMW we used the template script provided by the SARTools for DeSeq2 analysis, where we specified first group of samples as the reference samples and second group as condition with a parametric mean variance and Benjamini-Hochberg method for *P* adjustment [43].

### Assembly-free workflow

For the assembly-free approach we used the Metatrans pipeline [9], which uses FragGeneScan [44] for ORF predictions in short reads, CD-HIT [45] for gene clustering, and Diamond [28] for alignment against the M5nr, CAZy, and NCyc [11–13] databases. We then used the same annotation script, which Is included in CoMW. For expression analysis gene counts were normalized between samples using the DESeq2 [41] algorithm. Significantly differentially expressed genes were analysed in SARTools [42] using a parametric relationship and *P*-value of 0.05 as significance threshold. The Benjamini-Hochberg correction procedure [43] was used to adjust *P*-value. For parameters and versions of tools used in Metatrans see supplementary GitHub repository in data availability.

### Composition of simulated communities

In this study we used a set of simulated communities from Martinez et al. [9], who collected 4,943 genes (coding regions) from 5 abundant microbial genomes: *Bacteroides vulgatus* ATCC 8482, *Ruminococcus torques* L2–14, *Faecalibacterium prausnitzii* SL3/3, *Bacteroides thetaiotaomicron* VPI-5482, and *Parabacteroides distasonis* ATCC 8503. We simulated short reads into 100 samples using Polyester [46] embedded in a script provided by Martinez et al. [9] at coverage of 20×, which resulted in a count table and short reads with 2,395 genes to add the impact of sequencing coverage that the simulator mimics. The process of regulation of abundance was done by first dividing the 100 samples into 2 groups (“A” and “B”) and then the abundance of a randomly selected 10% of the genes was upregulated and downregulated ≤4-fold; in addition, we knocked out (0 abundance) 5% of genes completely from both simulated reads and count tables. The process of selection of samples and genes was random but tracked. To include quality and coverage bias, we used the ART simulator [47] that mimics the coverage bias, and thus some genes were removed to produce an equal number of reads in FASTQ format to those produced by Polyester. ART was initially trained with Hi-Seq 2500 Illumina quality error model from the aforementioned dataset to have a consistent error bias. After simulating FASTQ files we then extracted the quality data and bound them to the FASTA files, generating new FASTQ files. With the coverage bias and quality training included we had a total of 62,035,912 reads (310,179 ± 3,454 reads/sample).

### Evaluation measures

We used the standard measures of precision (aka positive predictive value), accounting for how many annotations and identifications of significantly differentially expressed gene families and subsystems are correct and defined as TP/(TP + FP), and recall (aka sensitivity or true positive [TP] rate), accounting for how many correct annotations are selected, defined as TP/(TP + FN), where TP indicates the number of orthologs that have been correctly annotated, FN indicates the number of orthologs/genes/functional subsystems that are in the simulated communities but were not found by a certain approach, and FP indicates the number of orthologs/genes/functional subsystems that have been wrongly annotated (because they do not appear in the simulated communities). The F-score is the harmonic mean of precision and recall, defined as (2 × Precision × Recall)/(Precision + Recall).

## Availability of source code and requirements

- **Project name**: Comparative Metatranscriptomics Workflow (CoMW)
- **Project home page**: https://github.com/anwarMZ/CoMW
- **Operating system(s)**: Platform independent
- **Programming language**: Python, R, and bash
- **Other requirements**: Requirements mentioned in detailed manual at GitHub
- **License**: GNU General Public License v3.0

## Availability of supporting data and materials

- An archival copy of the code and supporting data are available via the *GigaScience* database, GigaDB [48]
- Raw sequence data generated using simulation of full-length genes were deposited in the NCBI SRA and are accessible through BioProject accession number PRJNA509064
- Project supplementary scripts: https://github.com/anwarMZ/CoMW_supp
- CoMW is published as computational capsule on codeocean [17] and can be accessed through https://doi.org/10.24433/CO.1793842.v1
- CoMW is registered at SciCrunch.org with RRID:SCR_017109

## Additional files

**Supplementary File 1**–Precision recall analysis of both approaches.

**Supplementary File 2**–Differential expression analysis of all approaches using eggNOG database.

**Supplementary File 3**–Differential expression analysis of all approaches using CAZy database.

**Supplementary File 4**–Differential expression analysis of all approaches using NCyc database.

giz096_GIGA-D-19-00009_Original_Submission

giz096_GIGA-D-19-00009_Revision_1

giz096_GIGA-D-19-00009_Revision_2

giz096_Response_to_Reviewer_Comments_Original_Submission

giz096_Response_to_Reviewer_Comments_Revision_1

giz096_Reviewer_1_Report_Original_Submission Patrick May -- 2/10/2019 Reviewed

giz096_Reviewer_1_Report_Revision_1 Patrick May -- 6/10/2019 Reviewed

giz096_Reviewer_2_Report_Original_Submission Harriet Alexander -- 2/24/2019 Reviewed

giz096_Supplemental_Files

## Abbreviations

ABySS: Assembly By Short Sequences; BLAST: Basic Local Alignment Search Tool; BWA: Burrows-Wheeler Aligner; CAZy: Carbohydrate-Active EnZymes database; COMAN: Comprehensive Metatranscriptomics Analysis; eggNOG: Evolutionary Genealogy of Genes: Non-supervised Orthologous Groups; EMBOSS: European Molecular Biology Open Software Suite; FDR: false discovery rate; FN: false-negative result; FP: false-positive result; IMP: Integrated Meta-omic Pipeline; NCBI: National Center for Biotechnology Information; NCycDB: Nitrogen Cycling Database; ORF: open reading frame; SAMSA2: Simple Annotation of Metatranscriptomes by Sequence Analysis 2; SRA: Sequence Read Archive; TP: true-positive result; BTS: Bit-score

## Competing interests

The authors declare that they have no competing interests.

## Funding

This work was supported by a grant from the European Commission's Marie Sklowdowska Curie Actions program under project number 675546 (MicroArctic).

## Authors' contributions

M.Z.A. and C.S.J. conceived and designed the study. M.Z.A., T.B.A., and A.L. carried out the data production. M.Z.A. and A.L. carried out analysis. M.Z.A. drafted the manuscript, and A.L., T.B.A., and C.S.J. revised and approved the final version.

## References

[1] Coolen MJL, Orsi WD The transcriptional response of microbial communities in thawing Alaskan permafrost soils. Front Microbiol. 2015;6:197. 25852660 10.3389/fmicb.2015.00197 PMC4360760

[2] Gonzalez E, Pitre FE, Pagé AP, et al. Trees, fungi and bacteria: tripartite metatranscriptomics of a root microbiome responding to soil contamination. Microbiome. 2018;6:53. 29562928 10.1186/s40168-018-0432-5 PMC5863371

[3] Gosalbes MJ, Durbán A, Pignatelli M, et al. Metatranscriptomic approach to analyze the functional human gut microbiota. PLoS One. 2011;6:e17447. 21408168 10.1371/journal.pone.0017447 PMC3050895

[4] Abu-Ali GS, Mehta RS, Lloyd-Price J, et al. Metatranscriptome of human faecal microbial communities in a cohort of adult men. Nat Microbiol. 2018;3:356. 29335555 10.1038/s41564-017-0084-4 PMC6557121

[5] Leimena MM, Ramiro-Garcia J, Davids M, et al. A comprehensive metatranscriptome analysis pipeline and its validation using human small intestine microbiota datasets. BMC Genomics. 2013;14:530. 23915218 10.1186/1471-2164-14-530 PMC3750648

[6] Poulsen M, Schwab C, Jensen BB, et al. Methylotrophic methanogenic Thermoplasmata implicated in reduced methane emissions from bovine rumen. Nat Commun. 2013;4:1428. 23385573 10.1038/ncomms2432

[7] Narayanasamy S, Jarosz Y, Muller EEL, et al. IMP: a pipeline for reproducible reference-independent integrated metagenomic and metatranscriptomic analyses. Genome Biol. 2016;17:260. 27986083 10.1186/s13059-016-1116-8 PMC5159968

[8] Jung JY, Lee SH, Jin HM, et al. Metatranscriptomic analysis of lactic acid bacterial gene expression during kimchi fermentation. Int J Food Microbiol. 2013;163:171–9. 23558201 10.1016/j.ijfoodmicro.2013.02.022

[9] Martinez X, Pozuelo M, Pascal V, et al. MetaTrans: an open-source pipeline for metatranscriptomics. Sci Rep. 2016;6:26447. 27211518 10.1038/srep26447 PMC4876386

[10] Almeida A, Mitchell AL, Tarkowska A, et al. Benchmarking taxonomic assignments based on 16S rRNA gene profiling of the microbiota from commonly sampled environments. GigaScience. 2018;7, doi:10.1093/gigascience/giy054. PMC5967554 29762668

[11] Wilke A, Harrison T, Wilkening J, et al. The M5nr: a novel non-redundant database containing protein sequences and annotations from multiple sources and associated tools. BMC Bioinformatics. 2012;13:141. 22720753 10.1186/1471-2105-13-141 PMC3410781

[12] Cantarel BL, Coutinho PM, Rancurel C, et al. The Carbohydrate-Active EnZymes database (CAZy): an expert resource for glycogenomics. Nucleic Acids Res. 2009;37:D233–8. 18838391 10.1093/nar/gkn663 PMC2686590

[13] Tu Q, Lin L, Cheng L, et al. NCycDB: a curated integrative database for fast and accurate metagenomic profiling of nitrogen cycling genes. Bioinformatics. 2019;35:1040–8. 30165481 10.1093/bioinformatics/bty741

[14] Schostag MD, Anwar MZ, Jacobsen CS, et al. Transcriptomic responses to warming and cooling of an Arctic tundra soil microbiome. bioRxiv. 2019, doi:10.1101/599233.

[15] Bang-Andreasen T, Anwar MZ, Lanźen A, et al. Total RNA-sequencing reveals multi-level microbial community changes and functional responses to wood ash application in agricultural and forest soil. bioRxiv. 2019, doi:10.1101/621557. PMC7028008 32009159

[16] Comparative Metatranscriptomics Workflow. https://github.com/anwarMZ/CoMW.

[17] Anwar MZ, Lanzen A, Bang-Andreasen T, et al. Comparative Metatranscriptomic Workflow (CoMW) [Source Code]. Code Ocean 2019, doi:10.24433/CO.1793842.v1. PMC6667343 31363751

[18] Aguiar-Pulido V, Huang W, Suarez-Ulloa V, et al. Metagenomics, metatranscriptomics, and metabolomics approaches for microbiome analysis. Evol Bioinform Online. 2016;12(Suppl 1):5–16. 27199545 10.4137/EBO.S36436 PMC4869604

[19] Ni Y, Li J, Panagiotou G COMAN: a web server for comprehensive metatranscriptomics analysis. BMC Genomics. 2016;17:622. 27515514 10.1186/s12864-016-2964-z PMC4982211

[20] Westreich ST, Treiber ML, Mills DA, et al. SAMSA2: a standalone metatranscriptome analysis pipeline. BMC Bioinformatics. 2018;19:175. 29783945 10.1186/s12859-018-2189-z PMC5963165

[21] Zhao Q-Y, Wang Y, Kong Y-M, et al. Optimizing de novo transcriptome assembly from short-read RNA-Seq data: a comparative study. BMC Bioinformatics. 2011;12:S2. 10.1186/1471-2105-12-S14-S2 PMC3287467 22373417

[22] Celaj A, Markle J, Danska J, et al. Comparison of assembly algorithms for improving rate of metatranscriptomic functional annotation. Microbiome. 2014;2:39. 25411636 10.1186/2049-2618-2-39 PMC4236897

[23] Grabherr MG, Haas BJ, Yassour M, et al. Trinity: reconstructing a full-length transcriptome without a genome from RNA-Seq data. Nat Biotechnol. 2011;29:644–52. 21572440 10.1038/nbt.1883 PMC3571712

[24] Namiki T, Hachiya T, Tanaka H, et al. MetaVelvet: an extension of Velvet assembler to de novo metagenome assembly from short sequence reads. Nucleic Acids Res. 2012;40:e155. 22821567 10.1093/nar/gks678 PMC3488206

[25] Schulz MH, Zerbino DR, Vingron M, et al. Oases: robust de novo RNA-seq assembly across the dynamic range of expression levels. Bioinformatics. 2012;28:1086–92. 22368243 10.1093/bioinformatics/bts094 PMC3324515

[26] Simpson JT, Wong K, Jackman SD, et al. ABySS: a parallel assembler for short read sequence data. Genome Res. 2009;19:1117–23. 19251739 10.1101/gr.089532.108 PMC2694472

[27] Luo R, Liu B, Xie Y, et al. SOAPdenovo2: an empirically improved memory-efficient short-read de novo assembler. GigaScience. 2012;1:18. 23587118 10.1186/2047-217X-1-18 PMC3626529

[28] Buchfink B, Xie C, Huson DH Fast and sensitive protein alignment using DIAMOND. Nat Methods. 2015;12:59–60. 25402007 10.1038/nmeth.3176

[29] Altschul SF, Gish W, Miller W, et al. Basic Local Alignment Search Tool. J Mol Biol. 1990;215:403–10. 2231712 10.1016/S0022-2836(05)80360-2

[30] Zhao Y, Tang H, Ye Y RAPSearch2: a fast and memory-efficient protein similarity search tool for next-generation sequencing data. Bioinformatics. 2012;28:125–6. 22039206 10.1093/bioinformatics/btr595 PMC3244761

[31] Sczyrba A, Hofmann P, Belmann P, et al. Critical Assessment of Metagenome Interpretation – a benchmark of computational metagenomics software. Nat Methods. 2017;14:1063–71. 28967888 10.1038/nmeth.4458 PMC5903868

[32] Simonson AB, Servin JA, Skophammer RG, et al. Decoding the genomic tree of life. Proc Natl Acad Sci U S A. 2005;102:6608–13. 15851667 10.1073/pnas.0501996102 PMC1131872

[33] Segata N, Izard J, Waldron L, et al. Metagenomic biomarker discovery and explanation. Genome Biol. 2011;12:R60. 21702898 10.1186/gb-2011-12-6-r60 PMC3218848

[34] Urich T, Lanzén A, Qi J, et al. Simultaneous assessment of soil microbial community structure and function through analysis of the meta-transcriptome. PLoS One. 2008;3(6):e2527. 18575584 10.1371/journal.pone.0002527 PMC2424134

[35] Li H, Durbin R Fast and accurate short read alignment with Burrows-Wheeler transform. Bioinformatics. 2009;25:1754–60. 19451168 10.1093/bioinformatics/btp324 PMC2705234

[36] Lau MCY, Harris RL, Oh Y, et al. Taxonomic and functional compositions impacted by the quality of metatranscriptomic assemblies. Front Microbiol. 2018;9:1235. 29973918 10.3389/fmicb.2018.01235 PMC6019464

[37] Griffiths-Jones S, Bateman A, Marshall M, et al. Rfam: an RNA family database. Nucleic Acids Res. 2003;31:439–41. 12520045 10.1093/nar/gkg006 PMC165453

[38] Nawrocki EP, Eddy SR Infernal 1.1: 100-fold faster RNA homology searches. Bioinformatics. 2013;29:2933–5. 24008419 10.1093/bioinformatics/btt509 PMC3810854

[39] Rice P, Longden I, Bleasby A EMBOSS: the European Molecular Biology Open Software Suite. Trends Genet. 2000;16:276–7. 10827456 10.1016/s0168-9525(00)02024-2

[40] Vaser R, Pavlović D, Šikić M SWORD—a highly efficient protein database search. Bioinformatics. 2016;32:i680–4. 27587689 10.1093/bioinformatics/btw445

[41] Love MI, Huber W, Anders S Moderated estimation of fold change and dispersion for RNA-seq data with DESeq2. Genome Biol. 2014;15:550. 25516281 10.1186/s13059-014-0550-8 PMC4302049

[42] Varet H, Brillet-Guéguen L, Coppée J-Y, et al. SARTools: a DESeq2- and EdgeR-based R pipeline for comprehensive differential analysis of RNA-Seq data. Plos One. 2016;11:e0157022. 27280887 10.1371/journal.pone.0157022 PMC4900645

[43] Benjamini Y, Hochberg Y Controlling the false discovery rate: a practical and powerful approach to multiple testing. J R Stat Soc Ser B Methodol. 1995;57:289–300.

[44] Rho M, Tang H, Ye Y FragGeneScan: predicting genes in short and error-prone reads. Nucleic Acids Res. 2010;38:e191. 20805240 10.1093/nar/gkq747 PMC2978382

[45] Li W, Godzik A CD-HIT: a fast program for clustering and comparing large sets of protein or nucleotide sequences. Bioinformatics. 2006;22:1658–9. 16731699 10.1093/bioinformatics/btl158

[46] Frazee AC, Jaffe AE, Langmead B, et al. Polyester: simulating RNA-seq datasets with differential transcript expression. Bioinformatics. 2015;31:2778–84. 25926345 10.1093/bioinformatics/btv272 PMC4635655

[47] Huang W, Li L, Myers JR, et al. ART: a next-generation sequencing read simulator. Bioinformatics. 2012;28:593–4. 22199392 10.1093/bioinformatics/btr708 PMC3278762

[48] Anwar MZ, Lanzén A, Bang-Andreasen T, et al. Supporting data for “To assemble or not to resemble—a validated Comparative Metatranscriptomics Workflow (CoMW).”. GigaScience Database. 2019 10.5524/100630. PMC6667343 31363751

