# Supplementary material for: To assemble or not to resemble—A validated Comparative Metatranscriptomics Workflow (CoMW)
Source: Gigascience. 2019 Jul 30;8(8):giz096. doi: 10.1093/gigascience/giz096 (PMC6667343; doi:10.1093/gigascience/giz096)
Supplement: giz096_GIGA-D-19-00009_Original_Submission [file giz096_giga-d-19-00009_original_submission.pdf]

GigaScience

To assemble or not to resemble – benchmarking of metatranscriptomic practices and a validated Comparative Metatranscriptomics Workflow (CoMW)

--Manuscript Draft--

**Manuscript Number:**	GIGA-D-19-00009
**Full Title:**	To assemble or not to resemble – benchmarking of metatranscriptomic practices and a validated Comparative Metatranscriptomics Workflow (CoMW)
**Article Type:**	Research
**Funding Information:**	H2020 Marie Skłodowska-Curie Actions (675546) — Mr. Muhammad Zohaib Anwar
**Abstract:**	Background<br><br>Metatranscriptomics has been used widely for investigation and quantification of microbial communities' activity in response to external stimuli. By assessing the genes expressed metatranscriptomics provides an understanding of the interactions between different major functional guilds and the environment. Metatranscriptomics typically utilize short sequence reads, which can either be directly aligned to external reference databases ("assembly-free approach") or first assembled into contigs before alignment ("assembly-based approach"). Here we compared workflows representing both alternatives, using simulated and real-world metatranscriptomes from Arctic and Temperate terrestrial environments. We evaluate their accuracy in precision and recall using generic and specialized hierarchical protein databases.<br><br>Results<br><br>We show that the assembly-based approach provides significantly fewer false positives resulting in more precise identification and quantification of functional genes in metatranscriptomes. Using the comprehensive database M5nr, the assembly-based approach identifies genes with only 0.6% false positives at thresholds ranging from inclusive to stringent compared to assembly-free approach (3.6 to 15% false positives). Using specialized databases (Carbohydrate Active-enzyme and Nitrogen Cycle) the assembly-based approach identifies and quantifies genes with 3-5x less false positives. We also evaluated the impact of both approaches on real-world datasets. Based on this benchmarking we present a standardized and optimized workflow for identifying functional genes from metatranscriptomes.<br><br>Conclusions<br><br>Our findings support the argument of assembling short reads into contigs before alignment to a reference database, since this provides higher precision and minimizes false positives. By virtue of the extensive benchmarking we also present the open source metatranscriptomics analysis workflow Comparative Metatranscriptomics Workflow CoMW.
**Corresponding Author:**	Muhammad Zohaib Anwar<br>Aarhus University<br>Roskilde, Copenhagen DENMARK
**Corresponding Author Secondary Information:**	
**Corresponding Author's Institution:**	Aarhus University
**Corresponding Author's Secondary Institution:**	
**First Author:**	Muhammad Zohaib Anwar
**First Author Secondary Information:**	
**Order of Authors:**	Muhammad Zohaib Anwar

Powered by Editorial Manager® and ProduXion Manager® from Aries Systems Corporation

	Anders Lanzen
	Toke Bang-Andreasen
	Carsten Suhr Jacobsen
**Order of Authors Secondary Information:**	
**Additional Information:**	
**Question**	**Response**
Are you submitting this manuscript to a special series or article collection?	No
**Experimental design and statistics**<br><br>Full details of the experimental design and statistical methods used should be given in the Methods section, as detailed in our Minimum Standards Reporting Checklist. Information essential to interpreting the data presented should be made available in the figure legends.<br><br>Have you included all the information requested in your manuscript?	Yes
**Resources**<br><br>A description of all resources used, including antibodies, cell lines, animals and software tools, with enough information to allow them to be uniquely identified, should be included in the Methods section. Authors are strongly encouraged to cite Research Resource Identifiers (RRIDs) for antibodies, model organisms and tools, where possible.<br><br>Have you included the information requested as detailed in our Minimum Standards Reporting Checklist?	Yes
**Availability of data and materials**<br><br>All datasets and code on which the conclusions of the paper rely must be either included in your submission or deposited in publicly available repositories (where available and ethically	Yes

Powered by Editorial Manager® and ProduXion Manager® from Aries Systems Corporation

appropriate), referencing such data using a unique identifier in the references and in the “Availability of Data and Materials” section of your manuscript.<br><br>Have you have met the above requirement as detailed in our Minimum Standards Reporting Checklist?	

Powered by Editorial Manager® and ProduXion Manager® from Aries Systems Corporation

Manuscript

Click here to download Manuscript Anwar_CoMW_ManuscriptFile.docx

To assemble or not to resemble – benchmarking of metatranscriptomic practices and a validated Comparative Metatranscriptomics Workflow (CoMW)

Authors

Muhammad Zohaib Anwar[1]*

Anders Lanzen[2,3]

Toke Bang-Andreasen[1,4]

Carsten Suhr Jacobsen[1]*

Author Affiliations

1 Department of Environmental Science, Aarhus University RISØ Campus, Frederiksborgvej 399, 4000 Roskilde, Denmark

2 AZTI, Herrera Kaia, Pasaia, Spain

3 IKERBASQUE, Basque Foundation for Science, 48011 Bilbao, Spain

4 Department of Biology, University of Copenhagen, Copenhagen, Denmark

***Corresponding Authors:**

Muhammad Zohaib Anwar: mzanwar@envs.au.dk

Carsten Suhr Jacobsen: csj@envs.au.dk

Abstract

Background

Metatranscriptomics has been used widely for investigation and quantification of microbial communities' activity in response to external stimuli. By assessing the genes expressed metatranscriptomics provides an understanding of the interactions between different major functional guilds and the environment. Metatranscriptomics typically utilize short sequence reads, which can either be directly aligned to external reference databases ("assembly-free approach") or first assembled into contigs before alignment ("assembly-based approach"). Here we compared workflows representing both alternatives, using simulated and real-world metatranscriptomes from Arctic and Temperate terrestrial environments. We evaluate their accuracy in precision and recall using generic and specialized hierarchical protein databases.

Results

We show that the assembly-based approach provides significantly fewer false positives resulting in more precise identification and quantification of functional genes in metatranscriptomes. Using the comprehensive database M5nr, the assembly-based approach identifies genes with only 0.6% false positives at thresholds ranging from inclusive to stringent compared to assembly-free approach (3.6 to 15% false positives). Using specialized databases (Carbohydrate Active-enzyme and Nitrogen Cycle) the assembly-based approach identifies and quantifies genes with 3-5x less false positives. We also evaluated the impact of both approaches on real-world datasets. Based on this benchmarking we present a standardized and optimized workflow for identifying functional genes from metatranscriptomes.

Conclusions

Our findings support the argument of assembling short reads into contigs before alignment to a reference database, since this provides higher precision and minimizes false positives. By virtue of the extensive benchmarking we also present the open source metatranscriptomics analysis workflow Comparative Metatranscriptomics Workflow *CoMW*.

Key Words

Metatranscriptomics, Benchmarking, Assembly, Alignment, Precision, Recall, False positives

1. Introduction

Metatranscriptomics provides an unprecedented insight to complex functional dynamics of microbial communities in various environments. The method has been applied to study the microbial activity in thawing permafrost and the related biogeochemical mechanisms contributing to greenhouse gas emissions [1], and Gonzalez *et al.* [2] applied metatranscriptomics to evaluate root microbiome response to soil contamination. The method is typically used to identify, quantify and compare the functional response of microbial communities in natural habitats or in relation to environmental or physio-chemical impacts.

Using high-throughput sequencing techniques such as Illumina, metatranscriptomics offers a non PCR biased method for looking at transcriptional activity occurring within a complex and diverse microbial population at a specific point in time [3]. However, curation and annotation of this complex data has emerged as a major challenge. To date, several studies have used various analytic workflows. Typically, short sequence reads are utilized, which can either be individually aligned directly to external reference databases (hereafter "assembly-free") or assembled into longer contiguous fragments (contigs) for alignment (hereafter "assembly-based"). Various studies have used either of these two general approaches. For example, Jung *et al.* [4] used an assembly-free approach (with BWA [5] to map reads to reference genomes of lactic acid bacterial strains associated with the kimchi microbial community) while Poulsen *et al.* [6] used an assembly-based approach (using SHE-RA [7] assembly before aligning to protein database). Similarly, an open source pipeline developed by Martinez *et al.* [8] to analyze

metatranscriptomics data-sets also aligns short reads directly to the M5nr database [9] and provides eggNOG annotation [10]. Most of the studies have used an assembly-free approach [11] due to less computational expense in addition to lack of thorough comparison available. Since no independent and direct comparison between these two alternative approaches has been performed presently, various metatranscriptomics analysis approaches may at times produce inconsistent observations, even if identical databases are used in the analysis. Thus, standardization of computational analysis is necessary to enable further propagation of metatranscriptomics approaches and their integration into microbial ecology research. Benchmarking provides a critical view of the efficiency and precision of different workflows and use of simulated communities for benchmarking enables the analysis to be independent of experimental variation and biases [12].

Here, we compared the assembly-free vs. assembly-based approach using simulated datasets. We evaluated the accuracy of both approaches using precision, recall and False Discovery Rates (FDR) with three different databases ranging from a generic or inclusive to specialized database dedicated to structurally or functionally related functional families: 1) M5nr: an inclusive and comprehensive non-redundant protein database in combination with eggNOG hierarchical annotation 2) Carbohydrate-Active Enzymes (CAZymes) [13]: a database dedicated to describing the families of structurally-related catalytic and carbohydrate-binding modules of enzymes and 3) Nitrogen Cycling Database (NCycDB) [14] a specialized and manually curated database covering only N cycle genes. In order to estimate the consistency and variance in the results caused by the choice of approach we then applied them to real world metatranscriptomes from microbial communities in 1) active-layer permafrost soil from Svalbard and 2) Ash impacted

Danish Forest soil. With the help of this comprehensive benchmarking and comparative analysis we then standardized and developed an open source Comparative Metatranscriptomic Workflow (*CoMW).*

2. Findings

2.1 Evaluation

In order to compare the performance of the assembly-based and assembly-free approaches, and to standardize a workflow using either of these, we simulated community transcript data using 4943 full length genes provided by Martinez et al. [8]. We analyzed both approaches separately and compared against direct annotation of full-length genes. The full-length genes were annotated using all three databases (M5nr, CAZy and NCycDB) independently to classify them into functional subsystems and gene families. Figure 1 shows detailed workflow of comparative analysis using both approaches.

Figure 1: Flowchart illustrating the benchmarking scheme used for comparison of approaches. Red path indicates the full-length genes workflow, Green indicates the steps in assembly-based and Blue indicates the steps in the assembly-free approach.

2.1.1 Functional assignment

2.1.1.1 ***M5nr Alignment***

Full length genes of the simulated community dataset were aligned and identified into 671 unique eggNOG orthologs, belonging to 19 distinct functional subsystems (level II). At the default confidence threshold (BTS score 50) of Diamond [15], assembly-free approach produced alignments to 820 orthologs with a precision of 85% (14.9% FPs), whereas the assembly-based

approach identified 665 orthologs with a precision of 99.3% (0.6% FPs) at the default SWORD [16] confidence threshold of 1E-5. Repeating the alignments using a gradient of 15 varying confidence thresholds for each approach (Low - T_L, Medium - T_M and High – $T_{H;}$ 5 thresholds / category) resulted in dissimilar performance for both approaches. The precision and recall of assembly-based approach did not change from 99.3% and 98.5% respectively throughout all categories whereas the assembly-free approach had a maximum precision of 96.3% at T_M and decreases to 85% at T_L and T_H. The assembly-based approach also produced fewer (only 0.6%) FPs consistently compared to assembly-free approach of FPs ranging from 14.9% to minimum 3.6% at highest precision. Based on F-Score the most optimal alignment for each approach is given in Table 1, whereas detailed values for precision, recall, F-Score and FDR are listed in Supplementary Table S1. We then also evaluated both approaches by selectively removing sequences belonging to a certain functional subsystem from the M5nr database in a controlled manner (segmented cross validation) in order to replicate real world metatranscriptomes where a certain functional subsystem can be completely or partially absent from the reference database. We removed four (level II) subsystems ("[D] Cell cycle control, cell division, chromosome partitioning"; "[L] Replication, recombination and repair"; "[E] Amino acid transport and metabolism" and "[R] General function prediction only" and "[S] Function unknown"). The level II subsystems were removed one at a time realigning full-length genes and simulated reads using both Assembly-based and assembly-free approaches to the cropped database to compare identification consistency. In each validation round, the number of unique (eggNOG) orthologs identified by the assembly-based approach were consistent to full length

gene alignment whereas from the assembly-free approach, the orthologs dropped significantly along with its ability to recall TPs. Table 2 provides details for each validation cycle.

Table 1 : Mean of Precision, Recall and F Score for both approaches against all three databases. Bold emphasizes better precision, recall, F-Score and FDR in each category across approaches

Databases	Approach	Threshold	Threshold Category	Recall	Precision	F-Score	FDR (%)
eggNOG	Assembly-free	*BTS 120*	*Strict [TH]*	**0.9880**	0.9540	0.9707	4.5977
	Assembly-based	*1.00E-15*	*Strict [TH]*	0.9851	**0.9939**	**0.9895**	**0.6006**
CAZy	Assembly-free	*BTS 110*	*Strict [TH]*	0.3510	0.5325	0.4231	46.7433
	Assembly-based	*1.00E-08*	*Medium [TM]*	**0.8131**	**0.7759**	**0.7940**	**22.4096**
NCycDB	Assembly-free	*BTS150*	*Strict [TH]*	0.1666	0.0581	0.0862	94.1860
	Assembly-based	*1.00E-14*	*Strict [TH]*	**0.6666**	**0.8333**	**0.7407**	**16.6666**

Table 2 Selective removal of functional subsystems from eggnog database (segmented cross-validation) of approaches. Bold emphasizes better consistency across approaches

	Full Length Genes Unique Orthologs		Unique orthologs from the assembly-free approach		Unique Orthologs from the Assembly-based approach	
Complete Database	671		784		667	
[D] removed	628	93.59%	572	72.95%	**624**	**93.55%**
[L] removed	640	95.38%	584	74.48%	**636**	**95.35%**
[E] removed	640	95.38%	583	74.36%	**636**	**95.35%**
[R], [S] removed	347	51.7%	334	42.60%	**352**	**52.77%**

2.1.1.2 CAZY Alignment

Out of a total 2395 full length genes, 500 sequences were aligned to 395 unique functional genes in the CAZY database, which belonged to 130 gene families and were further classified as 7 enzyme classes. Using default confidence thresholds (BTS 50 & 1E-5), the assembly-free approach identified 765 functional genes belonging to 112 unique families and 6 enzyme classes with a precision of 28.5% (71.4% FPs). The assembly-based approach identified 488 functional genes from CAZY database that were classified into 147 gene families from 7 enzyme classes with a precision of 66% (FDR 33.9%) at the default confidence threshold. However, when we repeated

the process with 15 various confidence thresholds, precision improved consistently for the assembly-based approach and FPs decreased, whereas for assembly-free approach, precision dropped significantly with increasing confidence threshold (see Table 1 and Supplementary Table S2).

2.1.1.3 *NCycDB Alignment*

410 of 2395 full length genes aligned to this database, identified as 29 unique Nitrogen cycle genes and further belonging to 15 functional gene families in 5 pathways. Using default confidence thresholds, the assembly-free approach identified 1541 functional genes belonging to 25 functional gene families classified into 6 pathways with a precision of 0.9% (99% FPs). The assembly-based approach identified 42 Nitrogen cycle genes classified into 25 gene families from 6 pathways with a precision of 59.5% (40.4% FPs) at a default confidence threshold of 1E-5. Like comparisons against M5nr and CAZY we repeated the process with 15 different confidence thresholds for each approach. Precision improved significantly for the assembly-based approach at stringent thresholds whereas for the assembly-free approach, the best precision achieved was 5.8%. (Table 1, Supplementary Table S3).

2.1.2 *Expression Quantification*

We also compared the ability of both approaches to quantify the expression of identified transcripts by performing differential expression analysis of two groups in simulated communities and compared against the full-length gene expression simulated. We selected three best identification thresholds for both approaches based on highest F-Score and performed differential expression analysis using DESeq2 [16] algorithm in SARTools [17]. This analysis for both approaches was carried out against all three databases using the most specific level of

hierarchy in the respective databases in order to capture their ability to quantify expression levels of specific genes. According to full-length gene alignments against eggNOG, 123 genes were significantly upregulated and 270 were significantly downregulated. Using assembly-free approach with best F-Score 73 genes were up-regulated (precision 94.5%, 5.4% FPs) and 380 (precision 65.7%, 34.2% FPs) were down regulated. whereas in the assembly-based approach 99 (precision 94.9%, 5% FPs) genes were up-regulated and 249 (precision 97.1%, 2.8% FPs) down regulated. For CAZy database full-length genes 81 and 189 genes were significantly up and down regulated respectively. Using assembly-free approach 31 (precision 19.3%, 80.6% FPs) genes significantly up regulated and 137 genes (precision 52.5%, 47.4% FPs) whereas the assembly-based approach 83 (precision 71%, 28.9% FPs) genes were up-regulated and 191 (precision 73.8%, 26.1% FPs) genes were down regulated. In the NCyc database expression analysis, 3 and 14 genes were significantly up and down-regulated respectively. Using assembly-free approach 26 (precision 0%, 100% FPs) and 107 (precision 4.6%, 95.3% FPs) genes up and down regulated respectively, whereas using assembly-based approach 3 (precision 33.3%, 66.6% FPs) genes up regulated and 18 (precision 55.5%, 44% FPs) were down regulated. Precision, Recall and FDR for both approaches against all three databases are available in Supplementary Table S4. Additionally, we then collapsed the functional genes into functional subsystems and gene families to remove FPs produced due to identification of homologous proteins or proteins with multiple inheritance. Fold change (log2 transformed) was then calculated for each subsystem/gene family. (see Figure 3)

Figure 2: Differential Expression comparison of Assembly-free and Assembly-based approaches using A) M5nr database, B) NCycDB and C) CAZy database.

2.1.3 Real-World metatranscriptomes

To evaluate the effect of the two approaches on real world data, two metatranscriptomes from microbial communities were studied. The first study investigated the transcriptional response during warming from -10 °C to 2 °C and subsequent cooling of 2 °C to -10 °C of an Arctic tundra active layer soil from Svalbard, Norway . The aim of the study was to understand taxonomic and functional shifts in microbial communities caused by climate change in the Arctic. A pronounced shift during the incubation period was noticed by Schostag *et al.* [17] (under review Molecular Ecology, SRA Bio-Project: PRJNA417839) which was not replicated by the assembly-free approach. However, the assembly-based approach identified an increase of genes in "[P] Inorganic ion transport and metabolism". For cooling, the assembly-based approach also captured the upregulation and downregulation of genes related to "[J] Translation, ribosomal structure and biogenesis" and "[C] Energy production and conversion" respectively (Figure 6) unlike assembly-free approach. These findings may have implications for our understanding of carbon dioxide emission, nitrogen cycling and plant nutrient availability in Arctic soils.

Figure 3: Functional expression dynamics in Arctic permafrost soil identified against eggNOG functional subsystems using Assembly-based and Assembly-free approach

In the second study, Bang-Andreasen *et al.* [18] (under review ISME, SRA Bio-Project: PRJNA512608) investigated the effects of wood ash amendment on Danish forest soils. Ash was added in 3 different quantities (0/control, 3, 12 and 90 tonnes ash per hectare (t ha^{-1})). In addition to ash concentration, the effect over time was analysed in soil communities at 0, 3, 30 and 100

days after ash addition. This resulted in strong effects on functional expression as seen in Figure 7. Both approaches once again displayed varying results such as changes in genes related to eggNOG functional subsystem "[W] Extracellular structures". Assembly-free approach also identified 75% of genes as "[S] Function unknown" consistently unlike assembly-based.

Figure 4: Functional expression dynamics in Danish forest soil due to Ash amendment and time elapsed, identified against eggNOG functional subsystems using Assembly-based and Assembly-free approach

2.2 Standardized Workflow (CoMW)

By the virtue of thorough benchmarking we standardized, implemented, and validated a metatranscriptomic workflow (CoMW) using Assembly-based approach. The workflow was implemented by keeping in mind the databases and tools for each step are ever improving thus optional steps can be skipped, changed or even improved in a structural manner. CoMW is open source workflow written in python available at (https://github.com/anwarMZ/CoMW). It is based on four major steps: 1) Assembly and Mapping short reads to assembled contigs; 2) Filtering of contigs; 3) Gene Prediction and Alignment and 4) Annotation. These scripts make each step of the workflow straightforward and help to make these complex analyses more reproducible and the components re-useable in different contexts. Help regarding input, output and parameters is provided with each script and an overall tutorial is presented in the data repository at GitHub. We here wanted to build an open source work flow for metatranscriptomics analysis that can assist in analyzing large metatranscriptomics data. Processes like ORF detection, alignment against the database and calculations of the gene expression are vital in any metatranscriptomic analyses and are, therefore, present uniformly in all workflows. However, since we use the

assembly-based workflow where we assemble the reads into longer contigs we also propose a 2-step filtering of the contigs to remove any chimeric or false contig made as a result of assembly or sequencing error by removing contigs that have an expression level less than a specific threshold and to remove any potential non-coding RNA contigs assembled.

Assembly and Mapping of short reads back to assembled contigs is done using Trinity [19] and BWA [5] respectively. Various tools have been developed for metatranscriptome reconstruction that usually rely on graph-theory. Trinity however generates the most optimal assemblies for coding RNA reads [11,20,21]. However, the user can generate contigs by any assembler preferred but it can reduce the quality of the following steps such as alignment of contigs.

Filtering of Contigs is done to remove variance in sequences/samples. We can filter contig abundance data by removing all contigs with relative expression lower than a specific cutoff, e.g. 1% (selected based on dataset variance) of the number of sequences in the dataset with least number of sequences. This threshold is also flexible for different datasets and in some cases not required at all so CoMW allows user to bypass this step or change the threshold up and down based on data variation. The filtered contigs are subject to potential non-coding RNA filtration by aligning them against the RFam database [22] using infernal [23] which is a secondary-structure-aware aligner that predicts the secondary structure of RNA sequences and similarities based on the consensus structure models. Once again, the ncRNA filtering is an optional step in CoMW, though highly recommended in order to reduce FPs.

Gene Prediction and Alignment Transeq from EMBOSS [24] is used to predict probable open reading frames (ORFs) of the contigs (customizable, by default 6 per contig). We used SWORD [16] as alignment tool against reference databases. SWORD can be used in parallel based on

computational resources available and the aligned results are parsed and cut-off at a specific confidence threshold of combination of e-value and alignment length (usually 1e-5, can be changed given the assembly distribution in datasets).

Annotation of aligned transcripts from the previous step can be done using the databases such as eggNOG which is a hierarchically structured annotation using a graph-based unsupervised clustering available algorithm to produce genome wide orthology inferences. Aligned proteins are then placed into functional subsystems based on their best hits.), CAZy which is a knowledge-based resource specialized in the Glycogenomics, and NCycDB; a Nitrogen cycle database. This results in a count table with a contig and eggNOG ortholog or CAZy gene or NCyc gene having a certain count from each sample depending upon database used. This count table can be then used for differential expression using state-of-the-art expression analysis.

CoMW is based on the results and findings from comparison of approaches. However, it has multiple optional steps such as abundance based and non-coding RNA filtering which can be different in data sets from a different environment. Similarly, the scripts are designed to cater more than one assembler output to enable diverse range of environments to be studied.

3 Discussion

The application of metatranscriptomics is less common than other DNA-based genomics techniques and thus most analysis pipelines are built ad hoc. The majority of these pipelines follow the assembly-free approach [11] such as COMAN [25], Metatrans [8], and SAMSA2 [26]. The lack of thorough benchmarking studies and standardized workflows in metatranscriptomics has made it a more challenging task to analyze the typically big datasets produced. Previous

287 studies have compared *de novo* sequence assemblers including Trinity, MetaVelvet [27], Oases[28], AbySS [29] and SOAPden-ovo [30] but an independent comparison of the two different approaches based on including assembly or directly aligning reads (here "assembly-free") has been lacking. We have attempted to assist this decision-making for processing metatranscriptomic analysis by independently assessing the performance of the two for functional annotation and expression quantification against three databases ranging from inclusive to specialized.

With simulated samples comprised of genes collected from abundant genomes provided by Martinez *et al.* [8] we show that both approaches provide high recall rates against the general comprehensive database M5nr. However, the assembly-based approach provided a significantly better precision for identification and quantification. For relatively compact and specialized databases, recall and precision drop for both approaches (especially for the most compact database NCyc). However, the assembly-based approach still appeared to be more precise, meaning that fewer genes were mis-assigned against these database and significantly lower FPs were produced. The precision in identification and expressional fold change comparison of gene families and functional subsystems for simulated samples against all three databases confirmed that while an assembly step is challenging computationally it holds the potential to reveal information regarding the gene expressions that is not attainable without it.

Selecting a single best workflow or pipeline for all types of metatranscriptomics studies is not a straightforward affair, and we believe that choice of approach changes the outcome of study significantly as observed with real-world datasets from active-layer permafrost soil from Svalbard and Ash impacted Danish Forest soil. In addition to choosing the right workflow, combining that

with the appropriate reference database is equally important to ensure the best annotation performance. With databases specialized for one or more specific environments or functional categories assembly-free approach underperforms due to its inability to identify conserved sequences in reference database. We also show that assembly-free approach can increase the rate of FPs in annotation when a database is dominant in specific functional subsystem or does not possess certain category which can also lead to wrong estimation of fold change in expression In summary, we show that the choice of approach (assembly-free or assembly-based) and database significantly affects the quality of the identification, annotation and expression results. Given the impact of each of these variables, it is inevitable that it significantly affects the results of an individual study and comparison of across studies. By standardizing and developing CoMW we believe our work presented here further assists the microbial ecology research community to make more informed decisions about the most appropriate methodological approach to analyze large metatranscriptomic datasets with improved precision.

4 Methods

For the assembly-free approach we used the Metatrans pipeline [8], which uses FragGeneScan [31] for ORF predictions in short reads, CD-Hit [32] for gene clustering and Diamond [15] for alignment to the M5nr database. For assembly-based approach we assembled the simulated short reads using Trinity [19] which has been studied to outperform other de novo RNA-seq assemblers and aligned the resulting contigs using SWORD (an efficient protein database search implementation especially optimized for large databases) [16] against the M5nr [9], CAZy [13] and NCyc [14] database. We then wrote an annotation script which Is included in CoMW. For

expression analysis gene counts were normalized between samples using the DESeq2 [33] algorithm. Significantly differentially expressed genes were analyzed in SARTools [34] using parametric relationship and p-value 0.05 as significance threshold. The Benjamini and Hochberg correction procedure [35] was used to adjust p-value.

4.1 Composition of Simulated Communities

In this study we utilised a set of simulated communities from Martinez *et al*. [8] where they collected 4943 genes from five abundant microbial genomes: *Bacteroides vulgatus* ATCC 8482, *Ruminococcus torques* L2-14, *Faecalibacterium prausnitzii* SL3/3, *Bacteroides thetaiotaomicron* VPI-5482 and *Parabacteroides distasonis* ATCC 8503. We simulated short reads into 100 samples using Polyester [36] embedded in a script provided by Martinez *et al.* [8] at coverage of 20x which resulted in a count table and short reads with 2395 genes to add the impact of sequencing coverage. Their abundance was then regulated up and down and by knocking out few genes in a controlled manner in order to make the composition similar to real world metatranscriptomes. The process of regulation of abundance was done by first dividing the 100 samples into 2 groups ("A" and "B") and then increasing the abundance of 10% genes up to 4-fold, decreasing the abundance of another 10% of the genes 4-fold and completely removing another 5% of the genes from both simulated reads and count tables. The process of selection of samples and genes was random but tracked. To include quality bias, we used the ART simulator [37] to produce an equal number of reads in FASTQ format to those produced by Polyester. ART was initially trained with Hi-Seq 2500 Illumina quality error model from dataset discussed above to have a consistent error bias. After simulating FASTQ files we then extracted the quality data and bound it to the FASTA

files generating new FASTQ files. With the coverage bias and quality training included we had a total of 62,035,912 reads (310,179 ± 3,454 reads/sample).

4.2 Evaluation Measures

We used the standard measures of precision (also named positive predictive value, PPV), accounting for how many annotations and identifications of significantly differentially expressed gene families and subsystems are correct and defined as $\frac{TP}{TP+FP}$ and recall (also named sensitivity or true positive rate, TPR), accounting for how many correct annotations are selected, defined as $\frac{TP}{TP+FN}$ where TP indicates the number of orthologs that have been correctly annotated, FN indicates the number of orthologs/genes/functional subsystem which are in the simulated communities but were not found by a certain approach and FP indicates the number of orthologs/genes/functional subsystem that have been wrongly annotated (because they do not appear in the simulated communities). The F-score is the harmonic mean of precision and recall, defined as $2 * \frac{Precision * Recall}{Precision + Recall}$

Availability of source code and requirements

- Project name: Comparative Metatranscriptomics Workflow [*CoMW*]
- Project home page: https://github.com/anwarMZ/CoMW
- Operating system(s): Platform independent
- Programming language: Python, R, and bash
- Other requirements: Requirements mentioned in detailed manual at GitHub
- License: GNU General Public License v3.0

Availability of supporting data and materials

Raw sequence data generated using simulation of full-length genes were deposited in the NCBI Sequence Read Archive and are accessible through BioProject accession number PRJNA509064

All databases can be accessed in one place at http://tiny.cc/CoMW_DBs

Supplementary File 1 – Precision Recall Analysis of both approaches

Supplementary File 2 – Differential Expression Analysis of all approaches using eggNOG database

Supplementary File 3 – Differential Expression Analysis of all approaches using CAZy database

Supplementary File 4 – Differential Expression Analysis of all approaches using NCyc database

Declarations

--

List of abbreviations

FDR: False Discovery Rate, FP: False Positives, TP: True Positives, FN: False Negatives, mRNA: messenger RNA

Ethical Approval (optional)

Not applicable

Consent for publication

Not applicable

Competing Interests

The authors declare that they have no competing interests.

Funding

This work was supported by a grant from the European Commission’s Marie Sklowdowska Curie Actions program under project number 675546 (*MicroArctic*).

Author's Contributions

MZA & CSJ conceived and designed the study. MZA, TBA and AL carried out the data production. MZA and AL carried out analysis. MZA drafted the manuscript and AL, TBA and CSJ revised and approved the final version.

Acknowledgements

Authors would like to acknowledge MicroArctic project for the funding and would like to thank authors of Metatrans for providing the data used for simulation.

Authors' information (optional)

References

1. Coolen MJL, Orsi WD. The transcriptional response of microbial communities in thawing Alaskan permafrost soils. Front Microbiol [Internet]. 2015 [cited 2018 Oct 25];6. Available from: https://www.frontiersin.org/articles/10.3389/fmicb.2015.00197/full

2. Gonzalez E, Pitre FE, Pagé AP, Marleau J, Guidi Nissim W, St-Arnaud M, et al. Trees, fungi and bacteria: tripartite metatranscriptomics of a root microbiome responding to soil contamination. Microbiome. 2018;6:53.

3. Leimena MM, Ramiro-Garcia J, Davids M, van den Bogert B, Smidt H, Smid EJ, et al. A comprehensive metatranscriptome analysis pipeline and its validation using human small intestine microbiota datasets. BMC Genomics. 2013;14:530.

4. Jung JY, Lee SH, Jin HM, Hahn Y, Madsen EL, Jeon CO. Metatranscriptomic analysis of lactic acid bacterial gene expression during kimchi fermentation. Int J Food Microbiol. 2013;163:171–9.

5. Langmead B, Trapnell C, Pop M, Salzberg SL. Ultrafast and memory-efficient alignment of short DNA sequences to the human genome. Genome Biol. 2009;10:R25.

6. Poulsen M, Schwab C, Jensen BB, Engberg RM, Spang A, Canibe N, et al. Methylotrophic methanogenic Thermoplasmata implicated in reduced methane emissions from bovine rumen. Nat Commun. 2013;4:1428.

7. Rodrigue S, Materna AC, Timberlake SC, Blackburn MC, Malmstrom RR, Alm EJ, et al. Unlocking Short Read Sequencing for Metagenomics. PLOS ONE. 2010;5:e11840.

8. Martinez X, Pozuelo M, Pascal V, Campos D, Gut I, Gut M, et al. MetaTrans: an open-source pipeline for metatranscriptomics. Sci Rep. 2016;6:26447.

9. Wilke A, Harrison T, Wilkening J, Field D, Glass EM, Kyrpides N, et al. The M5nr: a novel non-redundant database containing protein sequences and annotations from multiple sources and associated tools. BMC Bioinformatics. 2012;13:141.

10. Huerta-Cepas J, Szklarczyk D, Forslund K, Cook H, Heller D, Walter MC, et al. eggNOG 4.5: a hierarchical orthology framework with improved functional annotations for eukaryotic, prokaryotic and viral sequences. Nucleic Acids Res. 2016;44:D286–93.

11. Aguiar-Pulido V, Huang W, Suarez-Ulloa V, Cickovski T, Mathee K, Narasimhan G. Metagenomics, Metatranscriptomics, and Metabolomics Approaches for Microbiome Analysis. Evol Bioinforma Online. 2016;12:5–16.

12. Almeida A, Mitchell AL, Tarkowska A, Finn RD. Benchmarking taxonomic assignments based on 16S rRNA gene profiling of the microbiota from commonly sampled environments. GigaScience. 2018;7.

13. Cantarel BL, Coutinho PM, Rancurel C, Bernard T, Lombard V, Henrissat B. The Carbohydrate-Active EnZymes database (CAZy): an expert resource for Glycogenomics. Nucleic Acids Res. 2009;37:D233-238.

14. Tu Q, Lin L, Cheng L, Deng Y, He Z. NCycDB: a curated integrative database for fast and accurate metagenomic profiling of nitrogen cycling genes. Bioinforma Oxf Engl. 2018;

15. Buchfink B, Xie C, Huson DH. Fast and sensitive protein alignment using DIAMOND. Nat Methods. 2015;12:59–60.

16. Vaser R, Pavlović D, Šikić M. SWORD—a highly efficient protein database search. Bioinformatics. 2016;32:i680–4.

17. Schostag MD, Anwar MZ, Jacobsen CS, Larose C, Vogel TM, Maccario L, et al. Transcriptomic responses to warming and cooling of microbial communities in an Arctic tundra soil. Moleuclar Ecol. Under Review;

18. Bang-Andreasen T, Anwar MZ, Lanzen A, Kjoller R, Ronn R, Ekelund F, et al. Total RNA-sequencing reveals complex taxonomic and functional responses to wood ash application in agricultural and forest soil multi-level microbial communities. ISME. Under Review;

19. Grabherr MG, Haas BJ, Yassour M, Levin JZ, Thompson DA, Amit I, et al. Trinity: reconstructing a full-length transcriptome without a genome from RNA-Seq data. Nat Biotechnol. 2011;29:644–52.

20. Celaj A, Markle J, Danska J, Parkinson J. Comparison of assembly algorithms for improving rate of metatranscriptomic functional annotation. Microbiome. 2014;2:39.

21. Lau MCY, Harris RL, Oh Y, Yi MJ, Behmard A, Onstott TC. Taxonomic and Functional Compositions Impacted by the Quality of Metatranscriptomic Assemblies. Front Microbiol [Internet]. 2018 [cited 2018 Oct 25];9. Available from: https://www.ncbi.nlm.nih.gov/pmc/articles/PMC6019464/

22. Griffiths-Jones S, Bateman A, Marshall M, Khanna A, Eddy SR. Rfam: an RNA family database. Nucleic Acids Res. 2003;31:439–41.

23. Nawrocki EP, Eddy SR. Infernal 1.1: 100-fold faster RNA homology searches. Bioinformatics. 2013;29:2933–5.

24. Rice P, Longden I, Bleasby A. EMBOSS: The European Molecular Biology Open Software Suite. Trends Genet. 2000;16:276–7.

25. Ni Y, Li J, Panagiotou G. COMAN: a web server for comprehensive metatranscriptomics analysis. BMC Genomics. 2016;17:622.

26. Westreich ST, Treiber ML, Mills DA, Korf I, Lemay DG. SAMSA2: a standalone metatranscriptome analysis pipeline. BMC Bioinformatics. 2018;19:175.

27. Namiki T, Hachiya T, Tanaka H, Sakakibara Y. MetaVelvet: an extension of Velvet assembler to de novo metagenome assembly from short sequence reads. Nucleic Acids Res. 2012;40:e155.

28. Schulz MH, Zerbino DR, Vingron M, Birney E. Oases: robust de novo RNA-seq assembly across the dynamic range of expression levels. Bioinformatics. 2012;28:1086–92.

29. Simpson JT, Wong K, Jackman SD, Schein JE, Jones SJM, Birol İ. ABySS: A parallel assembler for short read sequence data. Genome Res. 2009;19:1117–23.

30. Luo R, Liu B, Xie Y, Li Z, Huang W, Yuan J, et al. SOAPdenovo2: an empirically improved memory-efficient short-read de novo assembler. GigaScience. 2012;1:18.

31. Rho M, Tang H, Ye Y. FragGeneScan: predicting genes in short and error-prone reads. Nucleic Acids Res. 2010;38:e191.

32. Li W, Godzik A. Cd-hit: a fast program for clustering and comparing large sets of protein or nucleotide sequences. Bioinforma Oxf Engl. 2006;22:1658–9.

33. Love MI, Huber W, Anders S. Moderated estimation of fold change and dispersion for RNA-seq data with DESeq2. Genome Biol [Internet]. 2014 [cited 2018 Oct 25];15. Available from: https://www.ncbi.nlm.nih.gov/pmc/articles/PMC4302049/

34. Varet H, Brillet-Guéguen L, Coppée J-Y, Dillies M-A. SARTools: A DESeq2- and EdgeR-Based R Pipeline for Comprehensive Differential Analysis of RNA-Seq Data. PLOS ONE. 2016;11:e0157022.

35. Benjamini Y, Hochberg Y. Controlling the False Discovery Rate: A Practical and Powerful Approach to Multiple Testing. J R Stat Soc Ser B Methodol. 1995;57:289–300.

36. Frazee AC, Jaffe AE, Langmead B, Leek JT. Polyester: simulating RNA-seq datasets with differential transcript expression. Bioinformatics. 2015;31:2778–84.

37. Huang W, Li L, Myers JR, Marth GT. ART: a next-generation sequencing read simulator. Bioinformatics. 2012;28:593–4.

Figure1 Click here to download Figure Figure 1.png

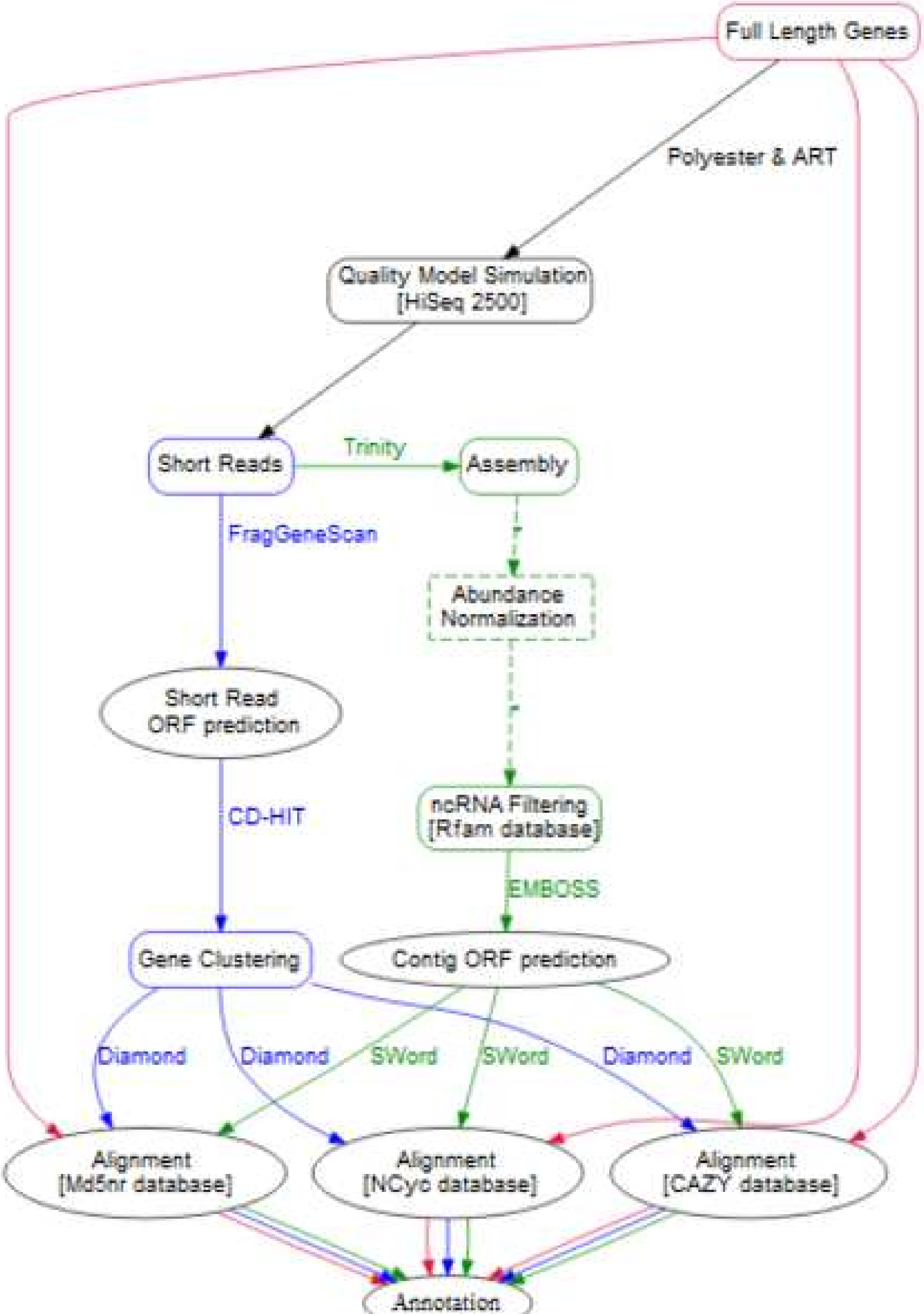

* Custom python scripts, added in CoMW

Figure2

Click here to download Figure Figure 2.tiff

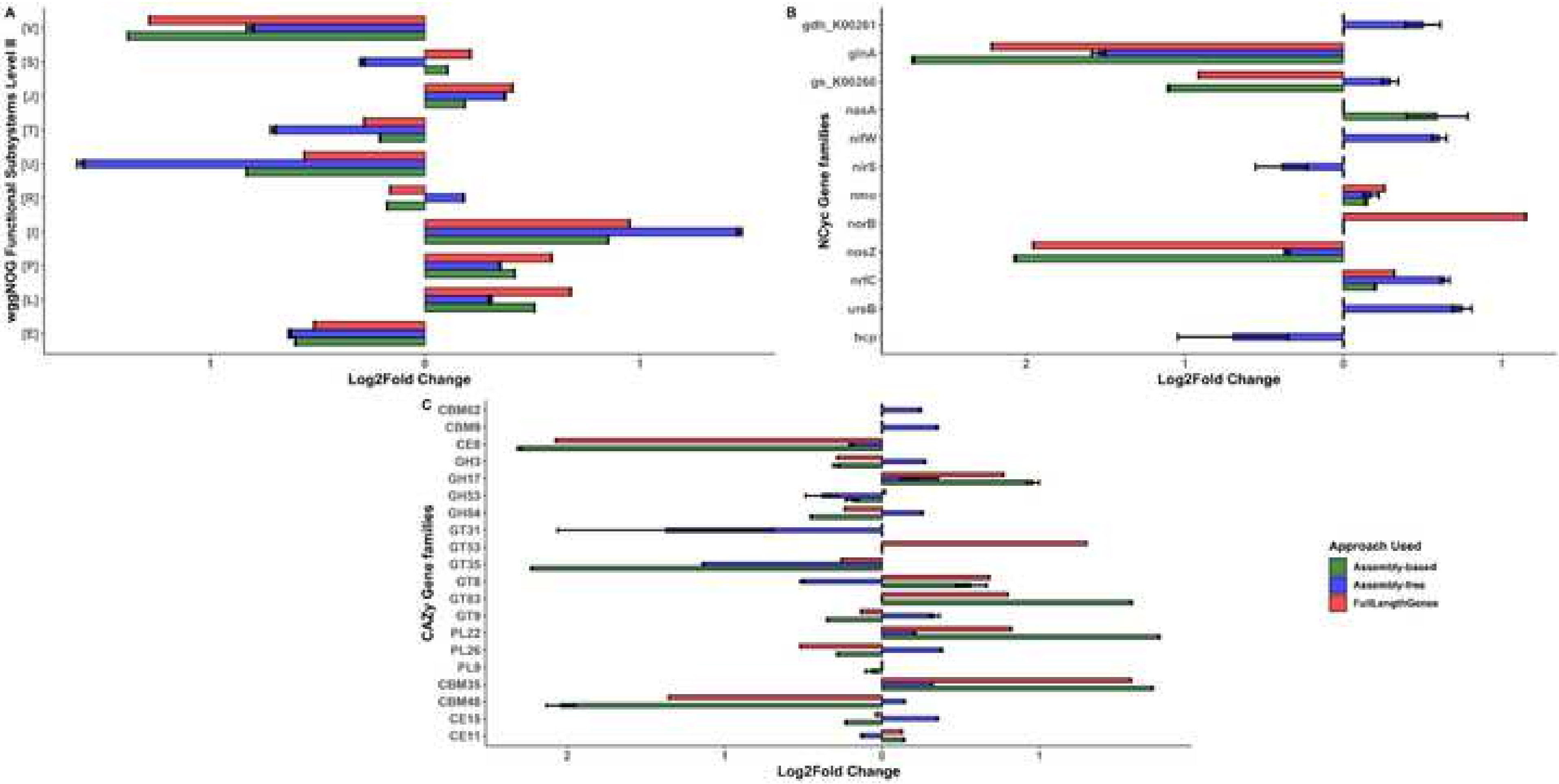

Figure3

Click here to download Figure Figure 3.tiff

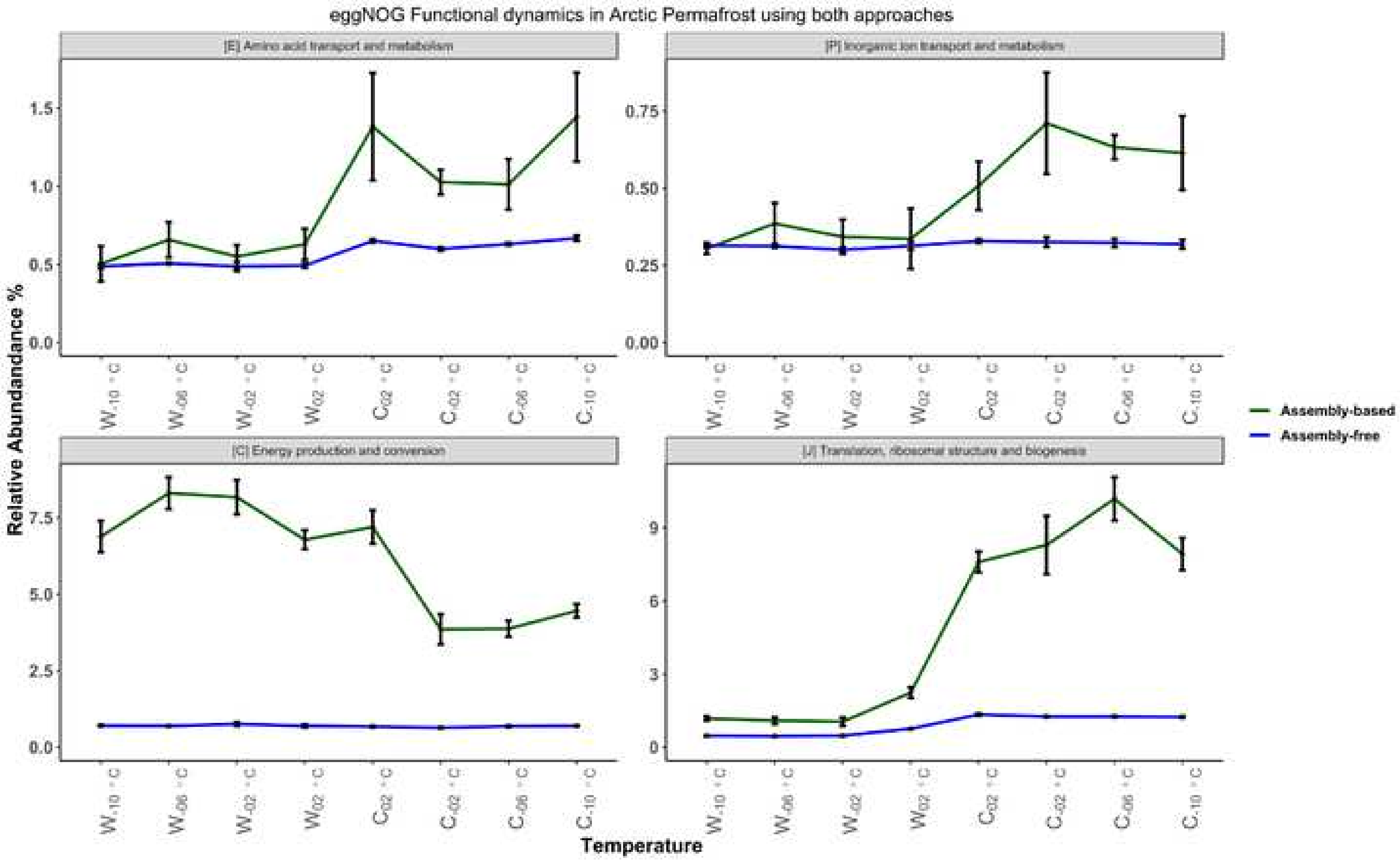

Figure4

Click here to download Figure Figure 4.tiff

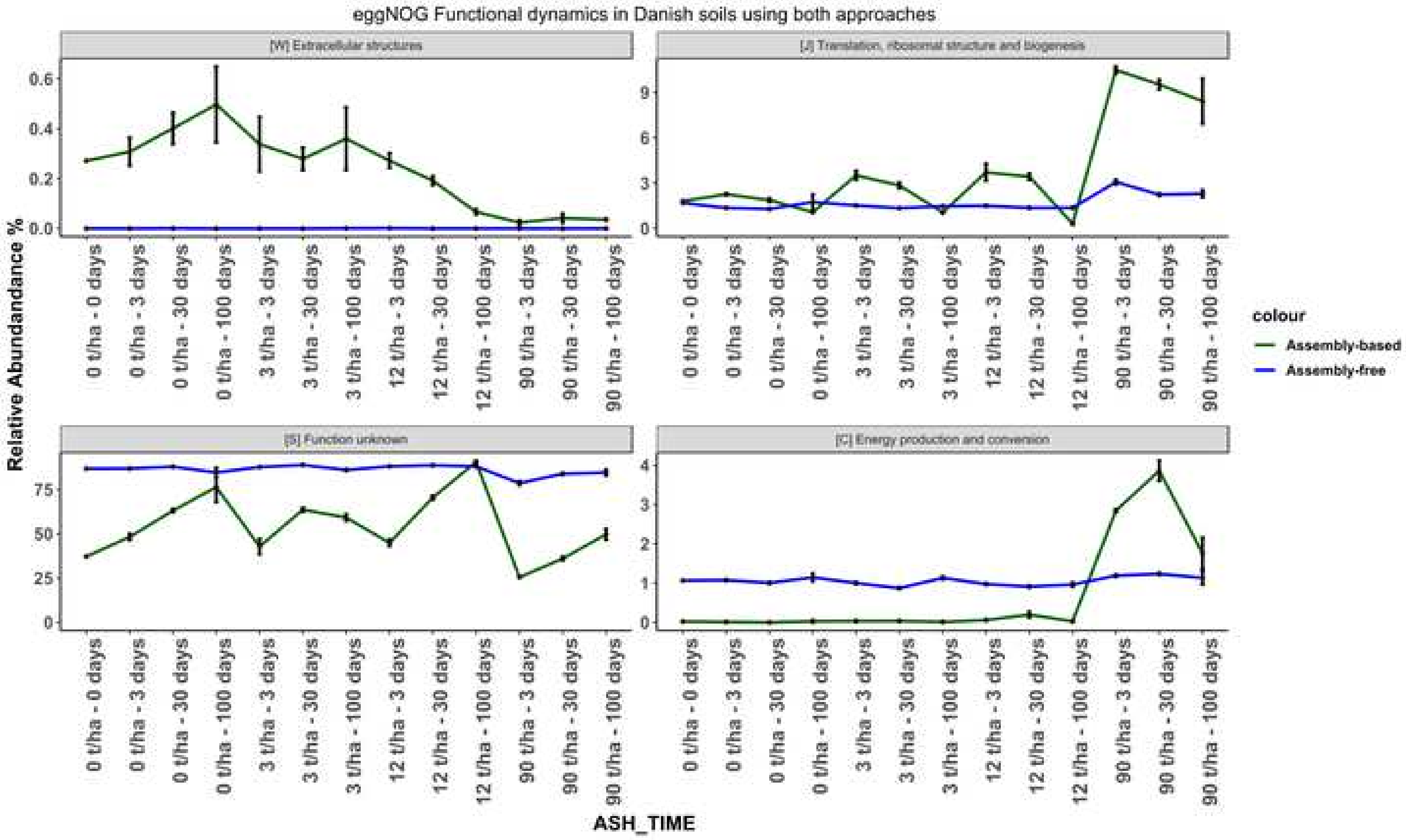

Click here to access/download
Supplementary Material
SupplementaryFile1_PrecisionRecall.docx

Click here to access/download
Supplementary Material
SupplementaryFile2_eggNOG_DEAnalysis.xlsx

Click here to access/download
Supplementary Material
SupplementaryFile3_CAZy_DEAnalysis.xlsx

Click here to access/download
Supplementary Material
SupplementaryFile4_NCyC_DEAnalysis.xlsx

Cover Letter Click here to download Personal Cover Anwar_Coverletter.pdf

AARHUS
UNIVERSITY
DEPARTMENT OF ENVIRONMENTAL SCIENCE

To: Editor, *GigaScience*

Re: Submission of manuscript entitled “To assemble or not to resemble – benchmarking of metatranscriptomic practices and a validated Comparative Metatranscriptomics Workflow (CoMW)” to *GigaScience*

Metatranscriptomics has recently gained popularity, thanks to its ability to uncover the active functional profiles of microbial communities. Being a relatively recent approach, there are still several analytical obstacles that limit its large-scale application. Sequence reference databases are also limited in their coverage and thus the use of different workflows and databases can lead to different outcomes, rendering it difficult to compare results between independent studies.

We have conducted a comprehensive comparison of workflows representing the two main alternatives for metatranscriptome analysis, namely assembly-based or assembly-free. This comparison was done using both simulated datasets and real world metatranscriptomes using three different hierarchical databases. To the best of our knowledge this is first independent comparison of these alternatives that will assist decision making and analysis of metatranscriptomics. Subsequently we also present a validated workflow using assembly-based analysis, which provided the best results according to simulated datasets.

We believe that GigaScience would be an outstanding forum for this manuscript, due to its intention of featuring interdisciplinary research; it would be of interest both to microbial ecologists, clinical microbiologists and bioinformaticians. To maintain open data and transparency in our benchmarking we have made all code, test data, results and supporting documents for CoMW available at different links provided within manuscript as per GigaScience policies. This manuscript presents material that has not previously been published and is not under consideration for publication elsewhere and all authors have seen and approved the final version submitted.

Thank you again for considering our manuscript.

Kind regards

Zohaib

Muhammad Zohaib Anwar
On behalf of Authors

Environmental microbiology & biotechnology

Muhammad Zohaib Anwar
PhD Student

Date: 10 January 2019

E-mail:
mzanwar@envs.au.dk
Web:
au.dk/en/mzanwar@envs

Sender's CVR no.:
31119103

SOLIDUM PETIT IN PROFUNDIS · UNIVERSITAS ARHUSIENSIS ·

Environmental microbiology & biotechnology
Aarhus University
Frederiksborgvej 399
PO box 358
DK-4000 Roskilde
Denmark

Tel.: +45 8715 0000
Fax: +45 8715 5010
E-mail: envs@au.dk
Web: envs.au.dk/en
