# Supplementary material for: To assemble or not to resemble—A validated Comparative Metatranscriptomics Workflow (CoMW)
Source: Gigascience. 2019 Jul 30;8(8):giz096. doi: 10.1093/gigascience/giz096 (PMC6667343; doi:10.1093/gigascience/giz096)
Supplement: giz096_GIGA-D-19-00009_Revision_1 [file giz096_giga-d-19-00009_revision_1.pdf]

GigaScience

To assemble or not to resemble – A validated Comparative Metatranscriptomics Workflow (CoMW)

--Manuscript Draft--

**Manuscript Number:**	GIGA-D-19-00009R1
**Full Title:**	To assemble or not to resemble – A validated Comparative Metatranscriptomics Workflow (CoMW)
**Article Type:**	Technical Note
**Funding Information:**	H2020 Marie Skłodowska-Curie Actions (675546) — Prof. Carsten Suhr Jacobsen
**Abstract:**	Background<br><br>Metatranscriptomics has been used widely for investigation and quantification of microbial communities' activity in response to external stimuli. By assessing the genes expressed, metatranscriptomics provide an understanding of the interactions between different major functional guilds and the environment. Here, we present de-novo assembly-based Comparative Metatranscriptomics Workflow (CoMW) implemented in a modular, reproducible structure, significantly improving the annotation and quantification of metatranscriptomes. Metatranscriptomics typically utilize short sequence reads, which can either be directly aligned to external reference databases ("assembly-free approach") or first assembled into contigs before alignment ("assembly-based approach"). We also compare CoMW (assembly-based implementation) with assembly-free alternative workflow, using simulated and real-world metatranscriptomes from Arctic and Temperate terrestrial environments. We evaluate their accuracy in precision and recall using generic and specialized hierarchical protein databases.<br><br>Results<br>CoMW provided significantly fewer false positives resulting in more precise identification and quantification of functional genes in metatranscriptomes. Using the comprehensive database M5nr, the assembly-based approach identified genes with only 0.6% false positives at thresholds ranging from inclusive to stringent compared to the assembly-free approach yielding up to 15% false positives. Using specialized databases (Carbohydrate Active-enzyme and Nitrogen Cycle), the assembly-based approach identified and quantified genes with 3-5x less false positives. We also evaluated the impact of both approaches on real-world datasets.<br><br>Conclusions<br>We present an open source de-novo assembly-based Comparative Metatranscriptomics Workflow (CoMW). Our benchmarking findings support the argument of assembling short reads into contigs before alignment to a reference database, since this provides higher precision and minimizes false positives.
**Corresponding Author:**	Muhammad Zohaib Anwar<br>Aarhus University<br>Roskilde, Copenhagen DENMARK
**Corresponding Author Secondary Information:**	
**Corresponding Author's Institution:**	Aarhus University
**Corresponding Author's Secondary Institution:**	
**First Author:**	Muhammad Zohaib Anwar
**First Author Secondary Information:**	
**Order of Authors:**	Muhammad Zohaib Anwar
	Anders Lanzen

	Toke Bang-Andreasen
	Carsten Suhr Jacobsen
**Order of Authors Secondary Information:**	
**Response to Reviewers:**	We thank the journal for conducting a thorough review of our manuscript, and value the thoughtful comments provided by the reviewers. Our responses to specific reviewer comments are below. We have attempted to address all comments individually and incorporated changes as needed. We have attached a response file to the file inventory that might be of help.<br>Comment = C, Response R<br><br>Editor:<br><br>C1: Although it is of interest, we are unable to consider it for publication in its current form. The reviewers have raised a number of points which we believe would improve the manuscript and may allow a revised version to be published in GigaScience. As a major concern the reviewers, in particular reviewer 2, highlight that many details, especially regarding the methods, are missing.<br><br>R1: We thank the editor and reviewers for their valuable feedback and thoughtful comments. We also acknowledge the missing details that were missing in manuscript and thus have subsequently made all details explicitly available in the manuscript and below in specific responses to reviewers with external links where appropriate. We also believe that now we have addressed every comment by improving the manuscript and supplementary information, please see specific answers to each comment below.<br><br>C2: They also recommend to package the tool with docker or conda, as they had some problems installing the software.<br><br>R2: Installation has been made significantly easier and reproducible using a conda environment as suggested. Now the user has to just create a conda environment with provided configuration file that includes all framework tools, libraries, dependencies and third-party tools along with optimized versions. A small bash script is also added which will download the databases directly from the FTP server https://mzacomw.au.dk hosted by Aarhus University.<br>> conda env create -f CoMW.yml<br>> source activate CoMW<br>> bash install.sh<br>A detailed answer is given to reviewers below. Also, an updated Readme and Manual are made available at the Github repository. See<br>https://github.com/anwarMZ/CoMW<br><br>C3: Please improve the description of methods and parameters.<br><br>R3: We have now also made a configuration file available that is used for the installation. This file also contains the versions of the libraries, channels, third party tools and versions of framework tools (e.g. python, R). Please see<br>https://github.com/anwarMZ/CoMW/blob/master/CoMW.yml.Parameters used are also now added in to the comments here or manuscript depending upon suitability.<br><br>C4: Please also share all supporting scripts and code (this can be done via GitHub, or via our repository GigaDB).<br><br>R4: All scripts are shared in the Github repository– https://github.com/anwarMZ/CoMW<br>Script used for simulating short reads from mock communities used is also now referenced in the text. The script used was made available by the Metatrans authors:<br>https://media.nature.com/original/nature-<br>assets/srep/2016/160523/srep26447/extref/srep26447-s1.doc Additional scripts for removing a functional subsystem from the database and SARTools differential expression analysis and parameters for running metatrans (assembly-free) approach are now added to separate Github-repo as suggested by the 2nd reviewer -<br>https://github.com/anwarMZ/CoMW_supp

C5: Submit code and data to code ocean as a “computational capsule” (https://codeocean.com/). Code ocean assigns DOIs, which you can cite in your GigaScience manuscript.

R5: As suggested, CoMW is now published as a peer-reviewed computational capsule at codeocean [1] and can be accessed through https://doi.org/10.24433/CO.1793842.v1

C6: Please also clarify the origin of the “real world datasets” and the papers referred to as being “under review” – is there a preprint that can be cited in the paper, if these are not yet published.

R6: We have now made both manuscripts available at BioRxiv. Authors MZA and CSJ are co-authors on both studies, whereas AL is co-author on one study led by TBA. Both the manuscripts contain experimental setup, details about the datasets and implementation of CoMW for the analysis. 1. Schostag, Morten Dencker, Muhammad Zohaib Anwar, Carsten Suhr Jacobsen, Catherine Larose, Timothy M. Vogel, Lorrie Maccario, Samuel Jacquiod, Samuel Faucherre, and Anders Priemé. "Transcriptomic responses to warming and cooling of an Arctic tundra soil microbiome." bioRxiv (2019): 599233. 2. Bang-Andreasen, Toke, Muhammad Zohaib Anwar, Anders Lanźen, Rasmus Kjøller, Regin Rønn, Flemming Ekelund, and Carsten Suhr Jacobsen. "Total RNA-sequencing reveals multi-level microbial community changes and functional responses to wood ash application in agricultural and forest soil." bioRxiv (2019): 621557.

C7: Both reviewers also feel that the overall structure of the manuscript needs improvement. Reviewer 1 got the impression that it reads almost as if being two separate manuscripts, with two separate aims. I recommend that you should consider this remark to improve the clarity of the paper and its message, how it all ties together.

R7: We appreciate the comments of both reviewers that remarked about this. In line with both, but more specifically as suggested by Reviewer 2, we have restructured the paper significantly to make it easier to read and to make the Methods section clearer. We first describe the overall implementation of CoMW, described in detail in Results, then the alternative approach, followed by the benchmarking and comparison between the two, using several reference databases for functional annotation, for both a simulated and a real-world dataset. We believe that now the manuscript with improved structure and previously missing details sync well for readers of GigaScience.

C8: In addition, please register any new software application in the SciCrunch.org database to receive a RRID (Research Resource Identification Initiative ID) number, and include this in your manuscript. This will facilitate tracking, reproducibility and re-use of your tool.

R8: CoMW is now registered at SciCrunch.org with RRID – SCR_017109. Also updated in the manuscript as per appropriate.

Reviewer 1:

C1: The authors use a previously published simulated dataset as well as two real-world metaT datasets from two environments. In recent years more and more microbiome studies are coming out using metaT instead of metagenomics (metaG) to describe active genes and species within microbial communities. Therefore, it is right time to benchmark different methods and analysis pipelines for metaT studies.

R1: We appreciate the acknowledgment of our work and constructive feedback. We have made improvements as suggested by the reviewer where necessary. Please see below for more detailed response to each comment.

C2: The main problem that I have with the paper is that it’s actually two papers, one

Powered by Editorial Manager® and ProduXion Manager® from Aries Systems Corporation

paper on testing. Different strategies for metaT analysis and one paper on a new analysis workflow for metaT analysis. For the second part, the CoMW pipeline, a more detailed description would be good, especially since it contains steps that are not necessary for the benchmark part before, e.g. the noncoding analysis. In general, I would strongly recommend to focus only on one of both parts and restructure the paper accordingly or have two separate papers.

R2: We appreciate the feedback and we have changed the overall structure of the paper to first of all present CoMW, then benchmark it using different sensitivity settings, and compare it to an assembly-free approach.

C3: The benchmarking part has the weakness that it is not really a comparison of existing tools or pipelines but of two strategies. The authors defined for each strategy the workflow they considered as the best, but obviously each strategy could be implemented with different tools and steps, and e.g. for the assembly-based strategy different tools and preprocessing steps can have a major impact for the resulting assemblies and therefore for all downstream analysis, but this is not really discussed here. For each strategy specific tools and parameters have been used. It should be clearer be stated why they have chosen, e.g. there are newer metaT assemblers than trinity, e.g. rnaspades and megahit, for the proposed benchmark I would at least want to see two or more implementations for each strategy.

R3: We agree with this comment, and thus we have restructured the manuscript significantly, as described above It should now be clearer that the idea and scope of this study is focused on elucidating the difference of using a typical assembly-free approach, to a typical assembly-based approach, specifically our implementation of CoMW, using the state-of-the-art tools for each strategy and evaluating them as holistically as possible. Additionally, several benchmarking studies have already been done for certain steps included in CoMW eg. Zhao et al. & Celaj et al. [2,3]. Thus, we have used Trinity as our assembler based on these benchmarking results. Similarly, for assembly-free approach we have used DIAMOND as short read mapper which has been thoroughly compared to its similar tools such as BLASTX and RAPSearch2 [4]. Megahit is metagenomic assembler and we believe should not be used for metatranscriptomics. However, one of the reasons to make CoMW modular was to make the users able to use an alternative tool (if preferred due to any reason) at certain step. For example, rnaspades can be used as an assembler and skip the step of assembling through CoMW.

C4: In the introduction examples from human gut studies are missing.

R4: We agree with the comment and have subsequently added two example studies from Gosalbes et al., and Abu-Ali et al. [5], [6] in the introduction of the manuscript (please refer to L55)

C5:A critical review of prior benchmarks in metaG and/or metaT should be given, e.g. CAMI should be mentioned (https://www.ncbi.nlm.nih.gov/pubmed/28967888) and the authors should discuss if the results for metaG and metaT can be compared and what are the specific problems of benchmarking metaT workflows.

R5: We now refer to CAMI [7] in the Discussion (L264 in manuscript). However, it is mainly benchmarking tools for certain steps in metagenomics analyses.

C6Why those three databases have been chosen, why not others.

R6: The databases were selected in order to include a representative selection of three different degrees of specialization, on a range from a more inclusive database with wide coverage (universality) and low degree of expert curation, to a smaller, highly curated database, with more narrow coverage. The three databases were also chosen because they are among the most widely used representing these categories. This is now also added to the introduction in manuscript. Please see L86-92.

C7:No discussion of other workflows available like anvi'o or IMP that could do similar things

R7: Both IMP and CoMW are modular can also be used together based on the preferred choice of the user. We have now clarified this in the Discussion and cited IMP. Please see L67 & from L266 in manuscript. Anvio is based on “maxBin” [8] which is a binning method mainly used for recovering individual genomes from metagenomes which is out of the scope of this study. IMP has some overlaps with our study, and some significant differences, which is now mentioned in the manuscript as well.

C8: The two real world datasets are only referenced as 'under review', either they should be submitted as preprint or added as supplement to the reviewers. Also, it is not clear if the authors are part of those studies.

R8: We agree and have now made both the manuscripts available at BioRxiv and have cited them in the revised manuscript. Please refer comment 6 to editor for more details.

C9: For the simulated dataset it is not totally clear how it was generated. Did the authors use only full-length genes or did they also include the non-coding parts of fully transcribed operon sequences as you would find expect them in real metaT datasets. This will have a big impact on the assembly quality, mapping and expression analysis.

R9: We used the full-length genes provided by the Martinez et al. [9]. (only coding part and not full operons). This is now explained in the manuscript (L368 onwards)

C10: How are the subsystems that were removed during the benchmarking procedure were chosen?

R10: Subsystems removed from each category were chosen on random to keep it unbiased removal. This is now updated in the manuscript along with reference to now updated script for the method. (L153 in manuscript)

C11:How the optimal alignment and mapping, e.g. BTS parameters were chosen for alignment and mapping?

R11: Both alignment and mapping were done on a range of parameters as described in Methods and specified in detail in supplementary file 1 to evaluate the performance for different (user selectable) sensitivity thresholds and avoid pre-selecting a certain threshold value. Assembled contigs were mapped from e-value 1E-1 to 1E-15. Similarly diamond alignment using short reads was tested on 15 BTS scores from BTS 10 to 150.

C12: The software is not straightforward to install, especially for non-bioinformaticians a real reproducible version using docker or conda would be highly appreciated. Also implementing the workflow as set of scripts should be transformed into a more formal structure using a workflow description like snakemake

R12: The software has now made significantly easier by using it in conda env and can be installed relatively easy in a separate conda container using the configuration file https://github.com/anwarMZ/CoMW/blob/master/CoMW.yml as described in the updated manuscript and attached CoMW manual. Now the user has to just create a conda env with provided configuration file that includes all framework tools, libraries, dependencies and third-party tools along with optimized versions and Run installation file

```
> conda env create -f configuration.yml
> source activate CoMW
> bash install.sh
```

Moreover, the aim of the workflow is to be modular and possible to adapt to alternative tools and databases. Though we appreciate the ease of use of Snakemake, especially for a user without expertise in python, we do not feel that this would allow for sufficient modularity. Instead CoMW is targeted to users with basic bioinformatics expertise comfortable to work in the command line, or for more advanced users with python expertise to allow changes in configuration, databases and programs used.

C13: Title is way too long and should be changed according the possible restructuring of the manuscript

Powered by Editorial Manager® and ProduXion Manager® from Aries Systems Corporation

R13: Suggestion well taken and since we have restructured the manuscript according to feedback we have also restructured the title a bit.

C14:Line 193: wrong figure seems to be referenced. Figure 3 should be Figure 2.
R14: We have changed the manuscript accordingly
C15:In paragraph 2.1.3 the reference to Figure 3 (not 6) is missing. Figures 3 and 4: colours not explained and unclear.
R15: We have corrected the reference to the figure and have improved the figure and caption.
C16: Numbers zero to nine should be given as words.
R16: We have corrected this style error throughout the manuscript.
C17: 132: Assembly-based should be lower case
R17: Correction well taken and we have updated for consistency all throughout the manuscript accordingly.
C18:Table 2: not clear when lower and upper case is used and why
R18: We apologize the unclear annotation and have changed incorrect uppercase words
C19: 161: Nitrogen upper case
R19: We have updated for consistency all throughout the manuscript accordingly.
C20:SARTOOLs has a different reference
https://www.ncbi.nlm.nih.gov/pmc/articles/PMC4900645/
R20: Thank you for pointing out. During in-text citation, one of the citations is mis-cited. Corrected now throughout
C21:Ref 21, reference style is wrong
R21: Correction well taken and references are consistent now
C22:Not clear how to handle references that are currently under review, should be put on bioarxiv e.g. reference 17 and 18
R22: Please see comment no 8 and comment 6 to editor. We have now made both manuscripts available on BioRxiv
C23: Description of required software versions is missing, e.g. which Java and Python version
R23: The missing information of versions is now part of configuration file along with all libraries and dependencies. See configuration file at
https://github.com/anwarMZ/CoMW/blob/master/CoMW.yml

Reviewer 2:

C1: My primary concern with the paper is that as it stands I am not sure that anyone would be able to replicate these analyses. Broadly, I think that the methods section is confusingly structured. I found myself skipping back and forth to try to figure out what you had done. The methods section should be restructured and organized to follow the flow of the analysis presented in the results. I suggest starting with a section describing how the mock data sets were created, then describe the use of the real-world meta-transcriptomes (which aren't well covered in the materials and methods), and then describe the two pipelines that were used including your novel CoMW.

R1: We appreciate the comments of the two reviewers that both remarked about this. In line with both, but more specifically as suggested by Reviewer 2, we have restructured the paper to make it easier to read and to make the Methods section clearer. We first describe the overall implementation of COMW, described in detail in Results, then the alternative approach, followed by the benchmarking and comparison between the two, using several reference databases for functional annotation, for both a simulated and a real-world dataset.

C2: Throughout the materials and methods, you should make sure that sufficient detail on all programs/tools used are included - meaning that the version of all software products should be included (e.g. DESeq2, Trinity, Diamond, etc.). Additionally, all parameters used should be included.

R2: We agree and have now made a configuration file available that is used for the installation. This file also contains the versions of the libraries, channels, third party tools and versions of framework tools (e.g. python, R). Please see this - https://github.com/anwarMZ/CoMW/blob/master/CoMW.yml Parameters used are also now added in manuscript depending upon suitability.

C3: Notably the description of the removal of "functional subsystems" was missing from the methods. How was this done?

R3: Subsystems to remove from each category were chosen on random to keep it unbiased removal.
https://mzacomw.au.dk/eggNOG.md52id2ont.zip
A file provided with eggNOG annotations was used to filter all sequences using script provided in supplementary repository,
https://github.com/anwarMZ/CoMW_supp/blob/master/remove_m5nr_subsystem.py
from database file by tracking the md5sum of Orthologous groups belonging to a certain functional subsystem. The manuscript has been updated to describe this in detail.

C4: I also do not think sufficient information was provided regarding how the mock RNA libraries were generated.

R4: These libraries were generated by Martinez et al. [9] and are described in more detail in the cited paper. Briefly, a subsample of 1000 genes from five different microorganisms was selected randomly, injected into Polyester to simulate short reads for two groups with different transcription levels, each containing 50 simulated samples.

C5: Moreover, the authors should clarify how the differential expression analysis was done-it appears to be absent from the paper.

R5: We used the template script provided by the SARTools for DESeq2 analysis.
https://github.com/PF2-pasteur-fr/SARTools/blob/master/template_script_DESeq2.r
now updated in the manuscript as well. L354 in manuscript

C6: Broadly speaking, I think it would be useful to have another github repo or a folder on the current github that contains all the supplementary scripts that were used for this publication (e.g. the Polyester scripts used, DE expression, etc.)

R6: All scripts are shared in the Github repository– https://github.com/anwarMZ/CoMW

One additional script used for simulating short reads from mock communities used is already made available by the Metatrans authors:
https://media.nature.com/original/nature-assets/srep/2016/160523/srep26447/extref/srep26447-s1.doc

C7: The pipeline is great as written-- but I do wonder why it is written in python and not implemented in some sort of workflow language/system (e.g pydoit, snakemake, etc.). If the goal is reproducibility, conda environments (or the like) should be included to facilitate the use of the same software versions throughout the workflow. Additionally, it would appear that the current scripts within CoMW are written to work with specific versions of each of the software packages covered (e.g. Trinity). What version were these scripts tested with? I tried running and ran into issues with Trinity that I think were due to the version I was trying to use. I suggest making a conda environment file (https://conda.io/projects/conda/en/latest/user-guide/tasks/manage-environments.html) that can be included in the Github repo to detail what version of each of the software programs should be used for the pipeline.

R8: The software has now made significantly easier by using it in conda environment and can be installed relatively easy in a separate conda container using the configuration file
https://github.com/anwarMZ/CoMW/blob/master/CoMW.yml as described in the updated manuscript and attached CoMW manual. Now the user has to just create a

	conda environment with provided configuration file that includes all framework tools, libraries, dependencies and third-party tools along with optimized versions and Run installation file<br>> conda env create -f CoMW.yml<br>> source activate CoMW<br>> bash install.sh<br><br>Moreover, the aim of the workflow is to be modular and possible to adapt to alternative tools and databases. Though we appreciate the ease of use of Snakemake, especially for a user without expertise in python, we do not feel that this would allow for sufficient modularity. Instead CoMW is targeted to users with basic bioinformatics expertise comfortable to work in the command line, or for more advanced users with python expertise to allow changes in configuration, databases and programs used.<br><br>C8: I am perhaps missing it-- but it would appear that no quality trimming of the raw short reads was used in this study. This is generally accepted as best practice. Please explain its omission from both pipelines in the text. More broadly, I think it should be added to the CoMW workflow.<br><br>R8: The real-world dataset was trimmed as described in the cited preprints [10,11] . However, we would also like to mention in general that quality trimming is not always desirable for alignment to a protein database when only doing so for the purpose of annotation, since it can decrease sensitivity by removing informative, though erroneous bases [12]. For assembly however, a gentle trimming based on the above paper is already included in Trinity by default.<br><br>C9: The comparisons made in the paper focus primarily upon functional analysis (e.g. the identification of functional gene sets), and the simulated data set that is used is not well-suited to taxonomic data. The comparisons made in the paper are interesting and valid-- but I think that the authors should make clear that they are only really testing functional annotation and not the resolution of taxonomic annotation.<br><br>R9: We agree that the simulated datasets are not suitable for taxonomic annotation and that taxonomic annotation is outside of the scope of this paper. We have now clarified this in a paragraph regarding taxonomic annotation in the Discussion. L295 in manuscript<br><br>C10:Looking at Table 1, I am inclined to say that eggNOG does pretty well in general with or without assembly and that the other databases perform less well. Moreover, that other databases seem to be more depend upon assembly vs. not. I am not sure that this shows a clear-cut win for assembly. Additionally, how much variation might be driven by mapping approach used (Diamond) as compared to some other short read mapper/blasting.<br><br>R10: We have improved the caption of the Table 1. However, our interpretation of these results differs from the reviewer's; annotations using eggNOG are not completely comparable or similar between both approaches, since the precision drops significantly at medium and higher threshold of confidence by adding excessive false positives. Whereas, for the assembly-based approach or CoMW the precision doesn't drop at all even at a very high threshold. In Table 1 we have only added the best value for each database based on the F-scores. Please see Supplementary table 1 to have a complete view. The false positives remain less than 1% compared to 15% using assembly-free approach. Also, if one considers another mapper's than Diamond they are less or equally sensitive as Diamond. Using more sensitive tools such as SWORD without denovo assembly is not possible given the computational restrain especially for large metaT datasets.<br><br>C11: One approach used frequently in the analysis of microbial communities dominated by prokaryotes is to sequence both the metaG and metaT of the community, assemble the metaG, and then map the metaT to the assembled metaG or binned genomes from the metaG (e.g. https://www.nature.com/articles/s41396-017-

	0041-5). It seems worthwhile to discuss how your approach differs from these approaches.<br><br>R11: We agree that this is possible and might be good idea in many cases. However, with CoMW, we aim to develop a workflow applicable to pure metatranscriptomics studies, providing a resource for de novo assembly and functional analysis. This is especially relevant for environments such as soil where the high diversity and amount of metabolically active organisms means that the sequencing depth needed for metaG assembly becomes economically unfeasible for many studies, especially when, as here, microeukaryotes are also targeted.<br><br>C12: Line 69: Without a meta-analysis I am not sure that this can be stated-citation 11 only lists 5 studies that take that approach in a fairly limited subset of studies. I would remove this statement unless you have solid numbers on how many studies use assembly vs mapping approaches? Also, you should qualify your extrapolation that it is "because of less computational expense." You don't know people's rationale for choosing one pipeline over another.<br><br>R12: We agree with the reviewer's comment and have removed the statement in introduction.<br><br>C13:Line 70: Some discussion of the CAMI metagenomic comparisons (https://data.cami-challenge.org/) seems justified here. They did extensive comparisons of assembly approaches as well as direct taxonomic analysis of short reads. The CAMI parallels your investigation of functional gene annotation nicely.<br>R13: We agree and now mention CAMI in manuscript L264, though as mentioned above, CAMI has a different focus and its benchmarks are mostly out of the scope of CoMW.<br><br><br>C14: Line 99: It would be helpful to the reader to directly state what the 'full-length genes' are. I am assuming that they are from the genome data that were used to generate the simulated data. The language should again be clarified in Line 110. One line 110 I am not sure what "full length genes" are being referred to. Perhaps changing the term used to "gold standard" or the like would help the reader follow the logical progression better?<br><br>R14: We have now revised the language in the lines as suggested, but prefer to keep the term "full length genes". It is now better explained to improve clarity.<br><br>C15: Line 112: Define BTS score.<br>R15: BTS = "bit score". This is now revised.<br><br>C16: Line 116: What numerical cutoffs were used for each of the Low, Medium, and High thresholds? Are the parameters detailed somewhere? I looked at the supplemental tables-but couldn't figure out which if any columns contained these values. Apologies if I missed them.<br><br>R16: They are specified as one column in each supplementary table in supplementary file 1. However, for more clarity, we have also mentioned here<br><br>Threshold Categoryassembly-basedassembly-free<br>Low – TL1E-1 – 1E-5BTS 10 – 50<br>Medium – TM 1E-6 – 1E-10BTS 60 – 100<br>High – TH1E-11 – 1E-15BTS 110 – 150<br><br>C17:Line 132: Watch capitalization of your two approaches throughout the paper ("Assembly-based and assembly-free approaches"). You change around quite a bit whether or not you capitalize.<br>R17: We have updated for consistency all throughout the manuscript accordingly.<br><br>C18: Line 152: As with line 116, what were the parameters used in the 15 "various confidence thresholds". I think it would be helpful to add a supplemental table that specifies the confidence thresholds used.R18: The parameters for alignment were

Powered by Editorial Manager® and ProduXion Manager® from Aries Systems Corporation

	consistent throughout the 15 confidence thresholds except the confidence threshold itself in both approaches. For the cut-off used or for the definition of the thresholds see response to comment # 16 above.<br><br>C19: Line 178: It is unclear to me how "full length" gene alignments were used for quantification? Was this based on mapping the simulated short reads against the original full-length genes that were annotated with eggNOG? Clarify in the methods or here.<br>R19: Yes exactly, simulated short reads were mapped back to the full-length genes (annotated using all three databases) for quantification and comparing the quantification using “assembly-based” (CoMW) and “assembly-free” methods. We have also updated in the methods for clarity.<br><br>C20: Line 204: What approach was used by Schostag? Was it assembly free? What was found by the assembly free approach?<br>R20: We have used assembly-based approach (CoMW) in Schostag et al. [10] as both MZA and CSJ are co-authors on the study. However, to elucidate the impact of the choice, we used “assembly-free” approach too and compared the results. Which is shown in figure 3 in manuscript<br><br>C21:Line 209: There is no Figure 6.<br>R21: Updated accordingly<br><br>C22:Line 209: There is no Figure 7.<br>R22: Updated accordingly<br><br>C23:Line 243: What was the estimated chimeric sequence frequency from your analysis of the mock datasets? What about false contigs? I would be surprised if they were terribly high as the mock data set didn't contain any species that had high average nucleotide identity.<br><br>R23: Simulated data didn’t have any chimeric sequences since Polyester does not simulate chimeric reads but the real data might have chimeric sequences based upon various things such as the de novo assembler used or environment under study or the dominating species. However, these contigs can be identified and filtered based on their abundance using the optional script provided filter_contigs_by_abundance.py where the user can identify a minimum threshold as a contig to not be considered as false contig based on its relative abundance to contigs pool.<br><br><br>C24: Line 282: Sure, I agree but do you have numbers to support this or a citation?<br>R24: We don’t have the exact numbers to support the argument however this review [13] of metaG, metaT and metaP approaches for microbiome analysis had a similar view. They mentioned that most of the metaT analysed studies have a custom/ad-hoc built analysis pipeline which can be due to many reasons (Computational expense, lack of computational experience, mRNA quality, databases of interest etc.). We have accordingly added the citation to the argument.<br><br>C25:Line 283: What are "these pipelines"? Metatranscriptomic pipelines? Also, I am not sure if you have the data to say that the majority of analyses use assembly-free approaches.<br>R25: Agreed that we don’t have a numerical data to confirm if the majority is using assembly-free approach so subsequently we have removed the statement.<br><br>C26: Line 324: Can you provide the parameters used in the various steps of the Metatrans pipeline? Also, do you have the scripts used to run the Metatrans pipeline? If so, they should be published somewhere (Supplemental text or GitHub repo for the paper).<br><br>R26: Metatrans package was downloaded and used from http://www.metatrans.org/ . We cited their manuscript [9] where they had mentioned the availability in abstract. The parameters for each part of the workflow are now made available at https://github.com/anwarMZ/CoMW_supp/blob/master/Metatrans_version_parameters.yml

C27: Line 336: Please provide the scripts used to generate the short reads.
R27: The script used was also available in supplementary material of Martinez et al. [9] direct link -https://media.nature.com/original/nature-assets/srep/2016/160523/srep26447/extref/srep26447-s1.doc
This script uses Polyester to simulate 100 samples in two groups (50 each) with varying expression.

C28: Line 341: Please clarify what "resulted in a count table and short reads with 2395 genes to add the impact of sequencing coverage" means. It is unclear to me what was produced and why only 2395 genes were recovered if 4943 were generated.
C28: Read simulator mimics the coverage bias and thus some genes were removed

C29:Lines 342: What does knocking out genes mean in the context of short reads? How was this done? Please publish the scripts used-- I couldn't find them in the GitHub repository.

R29:Knocking out is referring to the process of removing the abundance of randomly selected 5% of genes from the simulated data. 5% genes in the table were randomly set as 0 in 5 samples in the countable in order to mimic real data. Following that BioPython was used to remove reads belonging to that genes similarly from the simulated sequence files. We have now added this script to the supplementary repository. https://github.com/anwarMZ/CoMW_supp

C30:Line 353: Include the accession number for your simulated data set in the text as well as at the end of the paper.

R30: The information was added to the manuscript in Availability of Supporting data and Materials - Raw sequence data generated using simulation of full-length genes were deposited in the NCBI Sequence Read Archive and are accessible through BioProject accession number PRJNA509064. These reads are under the simulated Mock Communities classification of SRA

C31: Figure 1: You have "custom python scripts added in CoMW" at the bottom-but I can't see anything highlighted with that in the figure. It might be obscured because the figure is low resolution.
R31: We have improved the resolution of image. However, we don't think the "custom python scripts added in CoMW" are needed after the restructuring of manuscript and also now CoMW is easier to use for all steps after improving installation.

C32: Figure 2: Figure 2 is never referenced in the text. Also, I can't read this figure well-the text is very small and the resolution is not good. Generally, instead of plotting Log2Fold change I wonder if it would be more powerful to plot the difference relative to the Full-length gene analysis.
R32: We have improved the figure resolution and structure. However, we believe that since we want to compare both methods in relation to full-length genes and number of reads in both methods are not directly comparable, we use Log2Fold change instead of relative abundance of a transcript.

C33: Table 2: All tables should be able to stand alone with their caption without the text of the paper. This table is lacking some key information. What is the percentage column? What are the numbers (the number of orthologs retrieved with each method?) Label the letters-- specify what they are/where they are from. Check consistency in capitalization in titles. Specify that this is for eggNog? I also wonder if it would be better to report Recall/Precision values for the two different approaches as you did in Table 1-as it is a bit strange to recover more values in the assembly-based approach compared to the full-length genes but then not penalize what should be false discover.

R33: We have improved the readability of caption and tables throughout. For table 2 specifically, yes, the numbers show the unique homologs retrieved in Full length-genes and in both assembly-based and assembly-free methods. The letters were labelled in the text before, however as you correctly pointed out we have also added the full name of the letters in the table. We have also corrected the capitalization and caption. As suggested we have replaced the table 2 with Recall/Precision values in order to keep

the consistency and better understanding. As pointed out rightly, the values were misleading the fact that the False positives were not reported which is now corrected.

C34: Figures 3 and 4: The figure legends require more information: such as what is being plotted-relative abundance calculated how? Relative to all transcripts or relative to the study? It might also be interesting to add the relative abundance values that are reported in the original papers. These figures are interesting as the dynamic range of the data recovered is so different between the two different approaches. What do you think is the origin of this difference? Lack of mapping in the assembly free? Or spurious mapping of reads in the assembly free? It might be nice to add some more discussion of this in the text.

R34: Relative abundance of the functional subsystems in Figure 3 and 4 were calculated for each sample using both assembly-free and assembly-based methods. We have also updated the captions as per the reviewer has rightly pointed. Moreover, now that both manuscripts are made available at BioRxiv along with detailed supplementary methods we believe that the values can be seen for each dataset from the manuscripts. We have not added more information in order to keep clarity. We believe that our findings support our arguments that the main reason for the difference between the assembly-based and assembly-free approaches is the False positives produced by the assembly-free method. We saw that with simulated datasets it produced upto 15% False positives even with eggNOG databases which is inclusive and generic.

References:

1. Anwar MZ, Lanzen A, Bang-Andreasen T, Jacobsen CS. Comparative Metatranscriptomic Workflow (CoMW) [source code]. codeocean; 2019. Available from: https://doi.org/10.24433/CO.1793842.v1
2. Zhao Q-Y, Wang Y, Kong Y-M, Luo D, Li X, Hao P. Optimizing de novo transcriptome assembly from short-read RNA-Seq data: a comparative study. BMC Bioinformatics. 2011;12:S2.
3. Celaj A, Markle J, Danska J, Parkinson J. Comparison of assembly algorithms for improving rate of metatranscriptomic functional annotation. Microbiome. 2014;2:39.
4. Buchfink B, Xie C, Huson DH. Fast and sensitive protein alignment using DIAMOND. Nat Methods. 2015;12:59–60.
5. Gosalbes MJ, Durbán A, Pignatelli M, Abellan JJ, Jiménez-Hernández N, Pérez-Cobas AE, et al. Metatranscriptomic Approach to Analyze the Functional Human Gut Microbiota. PLOS ONE. 2011;6:e17447.
6. Abu-Ali GS, Mehta RS, Lloyd-Price J, Mallick H, Branck T, Ivey KL, et al. Metatranscriptome of human faecal microbial communities in a cohort of adult men. Nat Microbiol. 2018;3:356.
7. Sczyrba A, Hofmann P, Belmann P, Koslicki D, Janssen S, Dröge J, et al. Critical Assessment of Metagenome Interpretation – a benchmark of computational metagenomics software. Nat Methods. 2017;14:1063–71.
8. Wu Y-W, Tang Y-H, Tringe SG, Simmons BA, Singer SW. MaxBin: an automated binning method to recover individual genomes from metagenomes using an expectation-maximization algorithm. Microbiome. 2014;2:26.
9. Martinez X, Pozuelo M, Pascal V, Campos D, Gut I, Gut M, et al. MetaTrans: an open-source pipeline for metatranscriptomics. Sci Rep. 2016;6:26447.
10. Schostag MD, Anwar MZ, Jacobsen CS, Larose C, Vogel TM, Maccario L, et al. Transcriptomic responses to warming and cooling of an Arctic tundra soil microbiome. bioRxiv. 2019;599233.
11. Bang-Andreasen T, Anwar MZ, Lanźen A, Kjøller R, Rønn R, Ekelund F, et al. Total RNA-sequencing reveals multi-level microbial community changes and functional responses to wood ash application in agricultural and forest soil. bioRxiv. 2019;621557.
12. MacManes MD. On the optimal trimming of high-throughput mRNA sequence data. Front Genet [Internet]. 2014 [cited 2019 Apr 2];5. Available from: https://www.frontiersin.org/articles/10.3389/fgene.2014.00013/full
13. Aguiar-Pulido V, Huang W, Suarez-Ulloa V, Cickovski T, Mathee K, Narasimhan G. Metagenomics, Metatranscriptomics, and Metabolomics Approaches for Microbiome Analysis: Supplementary Issue: Bioinformatics Methods and Applications for Big

	Metagenomics Data. Evol Bioinforma. 2016;12s1:EBO.S36436.
**Additional Information:**	
**Question**	**Response**
Are you submitting this manuscript to a special series or article collection?	No
**Experimental design and statistics**<br><br>Full details of the experimental design and statistical methods used should be given in the Methods section, as detailed in our Minimum Standards Reporting Checklist. Information essential to interpreting the data presented should be made available in the figure legends.<br><br>Have you included all the information requested in your manuscript?	Yes
**Resources**<br><br>A description of all resources used, including antibodies, cell lines, animals and software tools, with enough information to allow them to be uniquely identified, should be included in the Methods section. Authors are strongly encouraged to cite Research Resource Identifiers (RRIDs) for antibodies, model organisms and tools, where possible.<br><br>Have you included the information requested as detailed in our Minimum Standards Reporting Checklist?	Yes
**Availability of data and materials**<br><br>All datasets and code on which the conclusions of the paper rely must be either included in your submission or deposited in publicly available repositories (where available and ethically appropriate), referencing such data using a unique identifier in the references and in the "Availability of Data and Materials" section of your manuscript.	Yes

Have you have met the above requirement as detailed in our Minimum Standards Reporting Checklist?	

Powered by Editorial Manager® and ProduXion Manager® from Aries Systems Corporation

Revised Manuscript
Click here to view linked References
Click here to access/download;Manuscript;CoMW_Revised_Manuscript.docx

To assemble or not to resemble – A validated Comparative Metatranscriptomics Workflow (CoMW)

Authors

Muhammad Zohaib Anwar[1]*

Anders Lanzen[2,3]

Toke Bang-Andreasen[1,4]

Carsten Suhr Jacobsen[1]*

Author Affiliations

1 Department of Environmental Science, Aarhus University RISØ Campus, Frederiksborgvej 399, 4000 Roskilde, Denmark

2 AZTI, Herrera Kaia, Pasaia, Spain

3 IKERBASQUE, Basque Foundation for Science, 48011 Bilbao, Spain

4 Department of Biology, University of Copenhagen, Copenhagen, Denmark

***Corresponding Authors:**

Muhammad Zohaib Anwar: mzanwar@envs.au.dk

Carsten Suhr Jacobsen: csj@envs.au.dk

Abstract

Background

Metatranscriptomics has been used widely for investigation and quantification of microbial communities' activity in response to external stimuli. By assessing the genes expressed, metatranscriptomics provide an understanding of the interactions between different major functional guilds and the environment. Here, we present *de-novo* assembly-based Comparative Metatranscriptomics Workflow (CoMW) implemented in a modular, reproducible structure, significantly improving the annotation and quantification of metatranscriptomes. Metatranscriptomics typically utilize short sequence reads, which can either be directly aligned to external reference databases ("assembly-free approach") or first assembled into contigs before alignment ("assembly-based approach"). We also compare CoMW (assembly-based implementation) with assembly-free alternative workflow, using simulated and real-world metatranscriptomes from Arctic and Temperate terrestrial environments. We evaluate their accuracy in precision and recall using generic and specialized hierarchical protein databases.

Results

CoMW provided significantly fewer false positives resulting in more precise identification and quantification of functional genes in metatranscriptomes. Using the comprehensive database M5nr, the assembly-based approach identified genes with only 0.6% false positives at thresholds ranging from inclusive to stringent compared to the assembly-free approach yielding up to 15% false positives. Using specialized databases (Carbohydrate Active-enzyme and Nitrogen Cycle), the assembly-based approach identified and quantified genes with 3-5x less false positives. We also evaluated the impact of both approaches on real-world datasets.

Conclusions

We present an open source *de-novo* assembly-based Comparative Metatranscriptomics Workflow (CoMW). Our benchmarking findings support the argument of assembling short reads into contigs before alignment to a reference database, since this provides higher precision and minimizes false positives.

Key Words

Metatranscriptomics, Benchmarking, Assembly, Alignment, Precision, Recall, False positives

1 Introduction

Metatranscriptomics provides an unprecedented insight to complex functional dynamics of microbial communities in various environments. The method has been applied to study the microbial activity in thawing permafrost and the related biogeochemical mechanisms contributing to greenhouse gas emissions [1], and Gonzalez *et al.* [2] applied metatranscriptomics to evaluate root microbiome response to soil contamination. Metatranscriptomics has also been used to study the functional human gut microbiota [3,4]. The method is typically used to identify, quantify and compare the functional response of microbial communities in natural habitats or in relation to environmental or physio-chemical impacts.

Using high-throughput sequencing techniques such as Illumina, metatranscriptomics offers a non PCR biased method for looking at transcriptional activity occurring within a complex and diverse microbial population at a specific point in time [5]. However, curation and annotation of this complex data has emerged as a major challenge. To date, several studies have used various analytic workflows. Typically, short sequence reads are utilized, which can either be individually aligned directly to external reference databases (hereafter “assembly-free”) or assembled into longer contiguous fragments (contigs) for alignment (hereafter “assembly-based”). Various studies have used either of these two general approaches. For example, Poulsen *et al.* [6] used an assembly-based approach. An open-source pipeline, IMP [7] also uses this approach in

integrated metagenomic and metatranscriptomic analyses. The assembly-free Approach has instead been used by e.g. Jung *et al.* [8], aligning short reads to reference genomes of lactic acid bacterial strains associated with the kimchi microbial community. Similarly, an open source pipeline developed by Martinez *et al.* [9] to analyse metatranscriptomics data-sets also aligns short reads directly to a protein database before annotation. The choice of either of these two alternatives for metatranscriptomics analyses may depend on lack of thorough comparisons. Since no independent and direct comparison between them has been performed presently, various metatranscriptomics analysis approaches may at times produce inconsistent observations, even if identical databases are used in the analysis. Thus, standardization of computational analysis is necessary to enable further propagation of metatranscriptomics approaches and their integration into microbial ecology research. Benchmarking provides a critical view of the efficiency and precision of different workflows and use of simulated communities for benchmarking enables the analysis to be independent of experimental variation and biases [10].

Here, we present Comparative Metatranscriptomic Workflow (CoMW) implemented using the *de-novo* assembly-based approach, standardized and validated for functional annotation and quantitative expression analysis. We validated the suitability of CoMW for functional analysis by comparing it to a typical assembly-free approach using simulated datasets and evaluated the accuracy of both approaches using precision, recall and False Discovery Rates (FDR). Three different protein databases were selected for this benchmarking in order to include a representative selection of three different degrees of specialization, on a range from a more inclusive database with wide coverage (universality) and low degree of expert curation, to a

smaller, highly curated database, with more narrow coverage: 1) M5nr [11] :-- an inclusive and comprehensive non-redundant protein database in combination with eggNOG hierarchical annotation 2) Carbohydrate-Active Enzymes (CAZymes) [12] :-- a database dedicated to describing the families of structurally-related catalytic and carbohydrate-binding modules of enzymes and 3) Nitrogen Cycling Database (NCycDB) [13] :-- a specialized and manually curated database covering only N cycle genes. Finally, in order to estimate the consistency and variance in the results caused by the choice of approach we then applied them to real world metatranscriptomes from microbial communities in 1) active-layer permafrost soil from Svalbard [14] and 2) Ash impacted Danish Forest soil [15].

2 Findings

2.1 **Comparative Metatranscriptomics Workflow (CoMW)**

We have standardized, implemented, and validated a metatranscriptomic workflow (CoMW) using de-novo assembly-based approach that can assist in analysing large metatranscriptomics data. It makes each step of the metatranscriptomic workflow straightforward and help to make these complex analyses more reproducible and the components re-useable in different contexts. The core processes such as ORF detection and alignment against the functional database are vital in any metatranscriptomic analyses and are, therefore, present uniformly in all workflows. However, since most of the tools performing these core processes are ever improving, the workflow is implemented in modular format in order to have the possibility of using alternative tools and databases if preferred or use a newer version of these tools. Modularity additionally also provides choice where optional steps can be skipped, changed or

even improved in a structural manner for example the scripts are designed to cater contigs from more than one assembler. In addition to core process CoMW has a couple of optional steps such as abundance based and non-coding RNA filtering which can be different in data sets from a different environment. CoMW is open source workflow written in python available at (https://github.com/anwarMZ/CoMW) and published as a computational capsule on codeocean [16]. An Anaconda cloud environment is created with the provided configuration file to install third-party tools and dependencies. Help regarding input, output and parameters is provided with each script and a comprehensive tutorial is presented in the GitHub repository.

2.2 *Evaluation of CoMW (assembly-based Approach) and comparison to an assembly-free method*

In order to compare the performance of the assembly-based workflow CoMW and assembly-free approaches, we simulated community transcript data using 4943 full length genes provided by Martinez *et al.* [9]. We analysed both approaches separately and compared against direct annotation of full-length genes. The full-length genes were annotated using all three databases (M5nr, CAZy and NCycDB) independently to classify them into functional subsystems and gene families. Figure 1 shows detailed workflow of comparative analysis using both approaches.

Figure 1: Flowchart illustrating the evaluation and benchmarking scheme used for the comparison of alternative approaches. Red path indicates the full-length genes workflow, Green indicates the steps in the assembly-based workflow CoMW and Blue indicates the steps in the assembly-free approach.

2.2.1 *Functional assignment*

M5nr Alignment Full length genes of the simulated community dataset were aligned and identified into 671 unique eggNOG orthologs, belonging to 19 distinct functional subsystems (level II). At the default confidence threshold (bit score 50), the, assembly-free approach produced alignments to 820 orthologs with a precision of 85% (14.9% FPs), whereas CoMW identified 665 orthologs with a precision of 99.3% (0.6% FPs) at the default confidence threshold of 1E-5. Repeating the alignments using a gradient of 15 varying confidence thresholds for each approach (Low - T_L, Medium - T_M and High – $T_{H;}$ five thresholds / category) resulted in dissimilar performance for both approaches. The precision and recall of CoMW did not decrease below 99.3% and 98.5% respectively throughout all categories whereas the assembly-free approach had a maximum precision of 96.3% at T_M and decreases to 85% at T_L and T_H. CoMW also produced fewer (only 0.6%) FPs consistently compared to the assembly-free Approach of FPs ranging from 14.9% to minimum 3.6% at highest precision. Based on F-Score the most optimal alignment for each approach is given in Table 1, whereas detailed values for precision, recall, F-Score and FDR are listed in Supplementary Table S1. We then also evaluated both approaches by selectively removing sequences belonging to a certain functional subsystem from the M5nr database in a controlled manner (segmented cross validation) in order to replicate real world metatranscriptomes where a certain functional subsystem can be completely or partially absent from the reference database. We removed four (level II) subsystems ("[D] Cell cycle control, cell division, chromosome partitioning"; "[L] Replication, recombination and repair"; "[E] Amino acid transport and metabolism" and "[R] General function prediction only" and "[S] Function

unknown”). The level II subsystems were randomly removed (see data availability for the script used for the removal) one at a time realigning full-length genes and simulated reads using both CoMW and assembly-free approaches to the cropped database to compare identification consistency. In each validation round, both precision and recall of CoMW were significantly higher than assembly-free approach. Recalling ability of assembly-free approach dropped significantly in this validation as compared to full database comparison. CoMW also produced less FPs as compared to assembly-free approach. Table 2 provides details for each validation cycle.

CAZY Alignment From 2395 full length genes, 500 sequences were aligned to 395 unique functional genes in the CAZY database, which belonged to 130 gene families and were further classified as seven enzyme classes. Using default confidence thresholds (BTS 50, 1E-5), the assembly-free approach identified 765 functional genes belonging to 112 unique families and six enzyme classes with a precision of 28.5% (71.4% FPs). CoMW identified 488 functional genes from CAZY database that were classified into 147 gene families from seven enzyme classes with a precision of 66% (FDR 33.9%) at the default confidence threshold. However, when we repeated the process with 15 various confidence thresholds, precision improved consistently and FPs decreased, whereas for the assembly-free approach, precision dropped significantly with increasing confidence threshold (see Table 1 and Supplementary Table S2).

NCycDB Alignment 410 out of 2395 full-length genes were aligned to this database, identified as 29 unique Nitrogen cycle genes and further belonging to 15 functional gene families in five pathways. Using default confidence thresholds, the assembly-free approach identified 1541 functional genes belonging to 25 functional gene families classified into six pathways with a

precision of 0.9% (99% FPs). CoMW identified 42 Nitrogen cycle genes classified into 25 gene families from six pathways with a precision of 59.5% (40.4% FPs) at a default confidence threshold of 1E-5. Like comparisons against M5nr and CAZY we repeated the process with 15 different confidence thresholds for each approach. Precision improved significantly for CoMW at stringent thresholds whereas for the assembly-free approach, the best precision achieved was 5.8%. (Table 1, Supplementary Table S3).

Table 1 Comparison of Precision, Recall, F Score and FDR for the assembly-free and the CoMW (assembly-based) approaches using all three databases based on best F-Score (Full table for both approaches and databases can be seen in Table S1, S2 and S3). Bold emphasizes better precision, recall, F-Score and FDR in each database between both approaches

Databases	Approach	Threshold	Threshold Category	Recall	Precision	F-Score	FDR (%)
eggNOG	assembly-free	*BTS 120*	*Strict [TH]*	**0.9880**	0.9540	0.9707	4.5977
	CoMW	*1.00E-15*	*Strict [TH]*	0.9851	**0.9939**	**0.9895**	**0.6006**
CAZy	assembly-free	*BTS 110*	*Strict [TH]*	0.3510	0.5325	0.4231	46.7433
	CoMW	*1.00E-08*	*Medium [TM]*	**0.8131**	**0.7759**	**0.7940**	**22.4096**
NCycDB	assembly-free	*BTS150*	*Strict [TH]*	0.1666	0.0581	0.0862	94.1860
	CoMW	*1.00E-14*	*Strict [TH]*	**0.6666**	**0.8333**	**0.7407**	**16.6666**

Table 2 Comparison of Precision, Recall, F Score and FDR for the assembly-free and CoMW (assembly-based) approaches using the selective removal of functional subsystems from eggNOG database (segmented cross-validation) to evaluate the consistency of both approaches. Bold emphasizes better consistency compared to Full length genes

Removed Subsystem	Approach	Recall	Precision	F-Score	FDR (%)
Cell wall/membrane/envelope biogenesis [M]	assembly-free	0.8726	0.9580	0.9133	4.1958
	CoMW	**0.9792**	**0.9855**	**0.9824**	**1.4423**
Replication, recombination and repair [L]	assembly-free	0.8734	0.9588	0.9141	4.1166
	CoMW	**0.9796**	**0.9858**	**0.9827**	**1.415**
Amino acid transport and metabolism [E]	assembly-free	0.8750	0.9589	0.9150	4.1095

	CoMW	**0.9812**	**0.9874**	**0.9843**	**1.2578**
General function prediction only and Function unknown [R], [S]	assembly-free	0.8933	0.9281	0.9104	7.1856
	CoMW	**0.9884**	**0.97443**	**0.9814**	**2.5568**

2.2.2 *Expression Quantification*

We also compared the ability of both approaches to quantify the expression of identified transcripts by performing differential expression analysis of two groups in simulated communities and compared against the full-length gene expression simulated. We selected three best identification thresholds for both approaches based on highest F-Score and performed differential expression analysis. This analysis for both approaches was carried out against all three databases using the most specific level of hierarchy in the respective databases in order to capture their ability to quantify expression levels of specific genes.

According to full-length gene alignments against eggNOG, 123 genes were significantly upregulated and 270 were significantly downregulated. According to the assembly-free Approach (with the best resulting F-Score), 73 genes were up-regulated (precision 94.5%, 5.4% FPs) and 380 (precision 65.7%, 34.2% FPs) were down regulated. whereas using the assembly-based Approach CoMW, 99 genes were identified as up-regulated (precision 94.9%, 5% FPs) and 249 down-regulated (precision 97.1%, 2.8% FPs). For the CAZy database full-length genes, 81 and 189 genes were identified as significantly up- and down regulated, respectively. Using the assembly-free approach 31 up-regulated (precision 19.3%, 80.6% FPs) and 137 down-regulated genes (precision 52.5%, 47.4% FPs) where identified, whereas the CoMW identified 83 (precision 71%, 28.9% FPs) and 191 (precision 73.8%, 26.1% Fps), respectively- In the NCyc database expression analysis, three and 14 genes were seen as significantly up and down-

regulated respectively using full-length genes. According to the assembly-free approach, 26 (precision 0%, 100% FPs) and 107 (precision 4.6%, 95.3% FPs) genes were up and down regulated respectively, whereas according to CoMW, three (precision 33.3%, 66.6% FPs) genes were up-regulated and 18 (precision 55.5%, 44% FPs) were down-regulated. Precision, Recall and FDR for both approaches against all three databases are available in Supplementary Table S4. Additionally, we collapsed the functional genes into functional subsystems and gene families to remove FPs produced due to identification of homologous proteins or proteins with multiple inheritance. Fold change (log2 transformed) was then calculated for each subsystem/gene family. (see Figure 2)

Figure 2: Differential Expression comparison of the assembly-free and the CoMW assembly-based approaches using A) M5nr database, B) NCycDB and C) CAZy database.

2.2.3 ***Real-World metatranscriptomes***

To evaluate the effect of the two approaches on real world data, two metatranscriptomes from microbial communities were studied. In the first study we investigated the transcriptional response during warming from -10 °C to 2 °C and subsequent cooling of 2 °C to -10 °C of an Arctic tundra active layer soil from Svalbard, Norway . The aim of the study was to understand taxonomic and functional shifts in microbial communities caused by climate change in the Arctic. A pronounced shift during the incubation period was noticed by Schostag *et al.* [14] which was not replicated by the assembly-free approach. However, using CoMW, we identified an increase of genes in the subsystem "[P] Inorganic ion transport and metabolism". During cooling, CoMW also captured the upregulation and downregulation of genes related to "[J]

Translation, ribosomal structure and biogenesis" and "[C] Energy production and conversion" respectively (Figure 3) unlike the assembly-free approach. These findings may have implications for our understanding of carbon dioxide emission, Nitrogen cycling and plant nutrient availability in Arctic soils.

Figure 3: Relative abundance of eggNOG functional subsystems in Arctic permafrost soil identified and quantified using both CoMW and the assembly-free approach compares the differences in observed functional dynamics. Blue dotted line represents trends using CoMW (assembly-based) whereas Red Solid line represents assembly-free approach

In the second study, we investigated the effects of wood ash amendment on Danish forest soils [15]. Ash was added in three different quantities (0/control, 3, 12 and 90 tonnes ash per hectare (t ha^{-1})) and the effect over time was analysed in soil communities at 0, 3, 30 and 100 days after ash addition. This resulted in strong effects on functional expression as seen in Figure 4. Both approaches once again displayed varying results such as changes in genes related to eggNOG functional subsystem "[W] Extracellular structures". assembly-free approach also identified 75% of genes as "[S] Function unknown" consistently unlike assembly-based.

Figure 4: Relative abundance of eggNOG functional subsystems in Ash deposited Danish forest soil with time identified using both the CoMW and an assembly-free approach. Blue dotted line represents trends using CoMW (assembly-based) whereas Red Solid line represents assembly-free approach

3 Discussion

The application of metatranscriptomics is less common than other DNA-based genomics techniques and thus most analysis pipelines are built *ad hoc* [17]. An assembly-free approach is

used in a few pipelines/workflows such as COMAN [18], Metatrans [9], and SAMSA2 [19] , while an assembly-based approach is used in a few such as IMP [7]. The lack of thorough benchmarking studies and standardized workflows in metatranscriptomics has made it a more challenging task to analyse the typically big datasets produced. Previous studies e.g. Zhao *et al.* & Celaj *et al.* [20,21] have compared *de-novo* sequence assemblers including Trinity [22], MetaVelvet [23], Oases [24], AbySS [25] and SOAPden-ovo [26]. Similarly, for assembly-free approach direct short read mappers have been compared thoroughly such as DIAMOND [27], BLASTX [28] and RAPSearch2 [29] but an independent comparison of the two different approaches based on including assembly or directly aligning reads (here "assembly-free") has been lacking. Critical Assessment of Metagenomic Interpreter (CAMI) [30] is so far the most comprehensive benchmarking effort, however it lacks any similar metatranscriptomics benchmarking. IMP [7] uses an integrated approach of metagenomics and metatranscriptomics and has some overlapping areas to CoMW and can be used together due to modular approach of CoMW.

Using simulated samples comprised of genes collected from abundant genomes provided by Martinez *et al.*, we show that both approaches provide similarly high recall rates against the general comprehensive database M5nr. However, CoMW provided a significantly better precision and a lower false discovery rate for identification and quantification. For relatively compact and specialized databases, recall and precision drop for both approaches (especially for the most compact database NCyc). Whereas, CoMW still appeared to be more precise, meaning that fewer genes were mis-assigned against these database and significantly lower FPs were produced.

We have attempted to assist this decision-making for processing metatranscriptomic analysis by independently assessing the performance of the two most common approaches and provide a road map for functional annotation and expression quantification against databases ranging from inclusive to specialized. The significantly higher precision in identification and quantification for gene families and functional subsystems in simulated samples, against all three databases, confirmed that while an assembly step is challenging computationally, it holds the potential to reveal information regarding the gene expressions that is not attainable without it. Selecting a single best workflow or pipeline for all types of metatranscriptomics studies is not a straightforward affair, and we believe that choice of approach changes the outcome of study significantly as observed with real-world datasets from active-layer permafrost soil from Svalbard and Ash impacted Danish Forest soil. In addition to choosing the right workflow, combining that with the appropriate reference database is equally important to ensure the best annotation performance. With databases specialized for one or more specific environments or functional categories, the assembly-free Approach under-performs due to its inability to identify alignments to homologs in the reference database. We also show that the assembly-free Approach can increase the FDR in annotation when a database is dominant in specific functional subsystem, which can also lead to wrong estimation of fold change in expression

While taxonomic annotation is beyond the scope of CoMW and thus our benchmarking analyses, it is important to consider the limited value of most functional genes for and thus functional metatranscriptomics alone for structural profiling of environmental communities, due to the high rate of horizontal gene transfer (HGT) [31]. Approaches for this purpose include

the identification of a limited set of "phylogenetic marker genes" (eg.[32]) or "total RNA" metatranscriptomics whereby the rRNA content is retained and utilized for taxonomic analysis [33]. Though not shown here, we expect that the former approach would also benefit in accuracy from assembling mRNA to full length transcripts before classification, based on our results regarding functional diversity. The total RNA approach also benefits from custom rRNA targeted assembly [15], which may be incorporated into CoMW thanks to its modularity.

In summary, we present the assembly-based workflow CoMW and show that this approach results in consistently better accuracy for functional analysis of metatranscriptomics data. Our benchmarking results show that the choice of approach (assembly-free *v* assembly-based) and database significantly affects the quality of the identification, annotation and expression results. Given the impact of each of these variables, it is inevitable that it significantly affects the results of an individual study and comparison of across studies. We believe that the work presented here will both provide a useful tool for and assist the microbial ecology research community to make more informed decisions about the most appropriate methodological approach to analyze large metatranscriptomic datasets with improved precision.

4 Methods

4.1 CoMW Implementation

CoMW (assembly-based) is based on four major steps: 1) *De-novo* Assembly and Mapping; 2) Filtering; 3) Gene Prediction and Alignment 4) Annotation.

De-novo Assembly and Mapping of short reads back to assembled contigs is done using Trinity [22] and BWA [34] respectively. Various tools have been developed for de-novo

metatranscriptome reconstruction that usually rely on graph-theory. Trinity however generates the most optimal assemblies for coding RNA reads [17,21,35]. Nevertheless, in CoMW, user can assemble short reads into contigs by any assembler preferred but it can reduce the quality of the following steps such as alignment of contigs.

Filtering of Contigs is done to remove variance in sequences/samples. Since CoMW is assembly-based, after we assemble the reads into longer contigs we also propose a 2-step filtering of the contigs to remove any chimeric or false contig made as a result of assembly or sequencing error by removing contigs that have an expression level less than a specific threshold and to remove any potential non-coding RNA contigs assembled. We can filter contig abundance data by removing all contigs with relative expression lower than a specific cut-off, e.g. 1% (selected based on dataset variance) of the number of sequences in the dataset with least number of sequences. This threshold is also flexible for different datasets and in some cases not required at all so CoMW allows user to bypass this step or change the threshold up and down based on data variation. The filtered contigs are subject to potential non-coding RNA filtration by aligning them against the RFam database [36] using infernal [37] which is a secondary-structure-aware aligner that predicts the secondary structure of RNA sequences and similarities based on the consensus structure models. Once again, the ncRNA filtering is an optional step in CoMW, though highly recommended in order to reduce FPs.

Gene Prediction and Alignment is done using Transeq from EMBOSS [38] to predict probable open reading frames (ORFs) of the contigs (customizable, by default six per contig). We used SWORD [39] as alignment tool against reference databases. SWORD can be used in parallel based on computational resources available and the aligned results are parsed and cut-off at a

specific confidence threshold of combination of e-value and alignment length (usually 1e-5, can be changed given the assembly distribution in datasets).

Annotation of aligned transcripts from the previous step can be done using the databases such as eggNOG which is a hierarchically structured annotation using a graph-based unsupervised clustering available algorithm to produce genome wide orthology inferences. Aligned proteins are then placed into functional subsystems based on their best hits.), CAZy which is a knowledge-based resource specialized in the Glycogenomics, and NCycDB; a Nitrogen cycle database. This results in a count table with a contig and eggNOG ortholog or CAZy gene or NCyc gene having a certain count from each sample depending upon database used. This count table can be then used for differential expression using state-of-the-art expression analysis suit such as DESeq2 [40] or its wrapper SARTools [41]. For evaluation of CoMW we used the template script provided by the SARTools for DeSeq2 analysis where we specified first group of samples as the reference samples and second group as condition with a parametric mean-variance and Benjamini & Hochberg method for P adjustment [42].

4.2 *Assembly-free Workflow*

For the assembly-free approach we used the Metatrans pipeline [9], which uses FragGeneScan [43] for ORF predictions in short reads, CD-Hit [44] for gene clustering and Diamond [27] for alignment against the M5nr, CAZy and NCyc [11–13] database. We then used the same annotation script which Is included in CoMW. For expression analysis gene counts were normalized between samples using the DESeq2 [40] algorithm. Significantly differentially expressed genes were analysed in SARTools [41] using parametric relationship and p-value 0.05 as significance threshold. The Benjamini and Hochberg correction procedure [42] was used to

adjust p-value. For parameters and versions of tools used in Metatrans see supplementary GitHub repository in data availability

4.3 Composition of Simulated Communities

In this study we utilised a set of simulated communities from Martinez *et al*. [9] where they collected 4943 genes (coding regions) from five abundant microbial genomes: *Bacteroides vulgatus* ATCC 8482, *Ruminococcus torques* L2-14, *Faecalibacterium prausnitzii* SL3/3, *Bacteroides thetaiotaomicron* VPI-5482 and *Parabacteroides distasonis* ATCC 8503. We simulated short reads into 100 samples using Polyester [45] embedded in a script provided by Martinez *et al.* [9] at coverage of 20x which resulted in a count table and short reads with 2395 genes to add the impact of sequencing coverage that the simulator mimics. The process of regulation of abundance was done by first dividing the 100 samples into two groups ("A" and "B") and then abundance of randomly selected 10% genes was regulated up- and down up to 4-folds, in addition to this we also knocked out (0 abundance) 5% genes completely from both simulated reads and count tables. The process of selection of samples and genes was random but tracked. To include quality and coverage bias, we used the ART simulator [46] that mimics the coverage bias and thus some genes were removed to produce an equal number of reads in FASTQ format to those produced by Polyester. ART was initially trained with Hi-Seq 2500 Illumina quality error model from dataset discussed above to have a consistent error bias. After simulating FASTQ files we then extracted the quality data and bound it to the FASTA files generating new FASTQ files. With the coverage bias and quality training included we had a total of 62,035,912 reads (310,179 ± 3,454 reads/sample).

4.4 Evaluation Measures

We used the standard measures of precision (also named positive predictive value, PPV), accounting for how many annotations and identifications of significantly differentially expressed gene families and subsystems are correct and defined as $\frac{TP}{TP+FP}$ and recall (also named sensitivity or true positive rate, TPR), accounting for how many correct annotations are selected, defined as $\frac{TP}{TP+FN}$ where TP indicates the number of orthologs that have been correctly annotated, FN indicates the number of orthologs/genes/functional subsystem which are in the simulated communities but were not found by a certain approach and FP indicates the number of orthologs/genes/functional subsystem that have been wrongly annotated (because they do not appear in the simulated communities). The F-score is the harmonic mean of precision and recall, defined as $\frac{2*Precision*Recall}{Precision+Recall}$.

Availability of source code and requirements

- Project name: Comparative Metatranscriptomics Workflow (*CoMW*)
- Project home page: https://github.com/anwarMZ/CoMW
- Operating system(s): Platform independent
- Programming language: Python, R, and bash
- Other requirements: Requirements mentioned in detailed manual at GitHub
- License: GNU General Public License v3.0

Availability of supporting data and materials

- Raw sequence data generated using simulation of full-length genes were deposited in the NCBI Sequence Read Archive and are accessible through BioProject accession number PRJNA509064
- Project supplementary scripts: https://github.com/anwarMZ/CoMW_supp
- Supplementary File 1 – Precision Recall Analysis of both approaches
- Supplementary File 2 – Differential Expression Analysis of all approaches using eggNOG database
- Supplementary File 3 – Differential Expression Analysis of all approaches using CAZy database
- Supplementary File 4 – Differential Expression Analysis of all approaches using NCyc database

Tracking and Reproducibility

- CoMW is published as computational capsule on codeocean and can be accessed through https://doi.org/10.24433/CO.1793842.v1

- CoMW is registered at SciCrunch.org with RRID – SCR_017109.

List of abbreviations

FDR: False Discovery Rate, FP: False Positives, TP: True Positives, FN: False Negatives, mRNA: messenger RNA

Ethical Approval

Not applicable

Consent for publication

Not applicable

Competing Interests

The authors declare that they have no competing interests.

Funding

This work was supported by a grant from the European Commission's Marie Sklowdowska Curie Actions program under project number 675546 (*MicroArctic*).

Author's Contributions

MZA & CSJ conceived and designed the study. MZA, TBA and AL carried out the data production. MZA and AL carried out analysis. MZA drafted the manuscript and AL, TBA and CSJ revised and approved the final version.

Acknowledgements

Authors would like to acknowledge European Commission's MicroArctic project for the funding. We would also like to thank authors of Metatrans for providing the data used for simulation. Additionally, we would like to thank Robert Vaser author of Sword to make it available on anaconda cloud and helping in integration with CoMW.

References

1. Coolen MJL, Orsi WD. The transcriptional response of microbial communities in thawing Alaskan permafrost soils. Front Microbiol. 2015;6.

2. Gonzalez E, Pitre FE, Pagé AP, Marleau J, Guidi Nissim W, St-Arnaud M, et al. Trees, fungi and bacteria: tripartite metatranscriptomics of a root microbiome responding to soil contamination. Microbiome. 2018;6:53.

3. Gosalbes MJ, Durbán A, Pignatelli M, Abellan JJ, Jiménez-Hernández N, Pérez-Cobas AE, et al. Metatranscriptomic Approach to Analyze the Functional Human Gut Microbiota. PLOS ONE. 2011;6:e17447.

4. Abu-Ali GS, Mehta RS, Lloyd-Price J, Mallick H, Branck T, Ivey KL, et al. Metatranscriptome of human faecal microbial communities in a cohort of adult men. Nat Microbiol. 2018;3:356.

5. Leimena MM, Ramiro-Garcia J, Davids M, van den Bogert B, Smidt H, Smid EJ, et al. A comprehensive metatranscriptome analysis pipeline and its validation using human small intestine microbiota datasets. BMC Genomics. 2013;14:530.

6. Poulsen M, Schwab C, Jensen BB, Engberg RM, Spang A, Canibe N, et al. Methylotrophic methanogenic Thermoplasmata implicated in reduced methane emissions from bovine rumen. Nat Commun. 2013;4:1428.

7. Narayanasamy S, Jarosz Y, Muller EEL, Heintz-Buschart A, Herold M, Kaysen A, et al. IMP: a pipeline for reproducible reference-independent integrated metagenomic and metatranscriptomic analyses. Genome Biol. 2016;17:260.

8. Jung JY, Lee SH, Jin HM, Hahn Y, Madsen EL, Jeon CO. Metatranscriptomic analysis of lactic acid bacterial gene expression during kimchi fermentation. Int J Food Microbiol. 2013;163:171–9.

9. Martinez X, Pozuelo M, Pascal V, Campos D, Gut I, Gut M, et al. MetaTrans: an open-source pipeline for metatranscriptomics. Sci Rep. 2016;6:26447.

10. Almeida A, Mitchell AL, Tarkowska A, Finn RD. Benchmarking taxonomic assignments based on 16S rRNA gene profiling of the microbiota from commonly sampled environments. GigaScience. 2018;7.

11. Wilke A, Harrison T, Wilkening J, Field D, Glass EM, Kyrpides N, et al. The M5nr: a novel non-redundant database containing protein sequences and annotations from multiple sources and associated tools. BMC Bioinformatics. 2012;13:141.

12. Cantarel BL, Coutinho PM, Rancurel C, Bernard T, Lombard V, Henrissat B. The Carbohydrate-Active EnZymes database (CAZy): an expert resource for Glycogenomics. Nucleic Acids Res. 2009;37:D233-238.

13. Tu Q, Lin L, Cheng L, Deng Y, He Z. NCycDB: a curated integrative database for fast and accurate metagenomic profiling of nitrogen cycling genes. Bioinforma Oxf Engl. 2018;

14. Schostag MD, Anwar MZ, Jacobsen CS, Larose C, Vogel TM, Maccario L, et al. Transcriptomic responses to warming and cooling of an Arctic tundra soil microbiome. bioRxiv. 2019;599233.

15. Bang-Andreasen T, Anwar MZ, Lanźen A, Kjøller R, Rønn R, Ekelund F, et al. Total RNA-sequencing reveals multi-level microbial community changes and functional responses to wood ash application in agricultural and forest soil. bioRxiv. 2019;621557.

16. Anwar MZ, Lanzen A, Bang-Andreasen T, Jacobsen CS. Comparative Metatranscriptomic Workflow (CoMW) [source code]. codeocean; 2019. Available from: https://doi.org/10.24433/CO.1793842.v1

17. Aguiar-Pulido V, Huang W, Suarez-Ulloa V, Cickovski T, Mathee K, Narasimhan G. Metagenomics, Metatranscriptomics, and Metabolomics Approaches for Microbiome Analysis: Supplementary Issue: Bioinformatics Methods and Applications for Big Metagenomics Data. Evol Bioinforma. 2016;12s1:EBO.S36436.

18. Ni Y, Li J, Panagiotou G. COMAN: a web server for comprehensive metatranscriptomics analysis. BMC Genomics. 2016;17:622.

19. Westreich ST, Treiber ML, Mills DA, Korf I, Lemay DG. SAMSA2: a standalone metatranscriptome analysis pipeline. BMC Bioinformatics. 2018;19:175.

20. Zhao Q-Y, Wang Y, Kong Y-M, Luo D, Li X, Hao P. Optimizing de novo transcriptome assembly from short-read RNA-Seq data: a comparative study. BMC Bioinformatics. 2011;12:S2.

21. Celaj A, Markle J, Danska J, Parkinson J. Comparison of assembly algorithms for improving rate of metatranscriptomic functional annotation. Microbiome. 2014;2:39.

22. Grabherr MG, Haas BJ, Yassour M, Levin JZ, Thompson DA, Amit I, et al. Trinity: reconstructing a full-length transcriptome without a genome from RNA-Seq data. Nat Biotechnol. 2011;29:644–52.

23. Namiki T, Hachiya T, Tanaka H, Sakakibara Y. MetaVelvet: an extension of Velvet assembler to de novo metagenome assembly from short sequence reads. Nucleic Acids Res. 2012;40:e155.

24. Schulz MH, Zerbino DR, Vingron M, Birney E. Oases: robust de novo RNA-seq assembly across the dynamic range of expression levels. Bioinformatics. 2012;28:1086–92.

25. Simpson JT, Wong K, Jackman SD, Schein JE, Jones SJM, Birol İ. ABySS: A parallel assembler for short read sequence data. Genome Res. 2009;19:1117–23.

26. Luo R, Liu B, Xie Y, Li Z, Huang W, Yuan J, et al. SOAPdenovo2: an empirically improved memory-efficient short-read de novo assembler. GigaScience. 2012;1:18.

27. Buchfink B, Xie C, Huson DH. Fast and sensitive protein alignment using DIAMOND. Nat Methods. 2015;12:59–60.

28. Altschul SF, Gish W, Miller W, Myers EW, Lipman DJ. Basic local alignment search tool. J Mol Biol. 1990;215:403–10.

29. Zhao Y, Tang H, Ye Y. RAPSearch2: a fast and memory-efficient protein similarity search tool for next-generation sequencing data. Bioinformatics. 2012;28:125–6.

30. Sczyrba A, Hofmann P, Belmann P, Koslicki D, Janssen S, Dröge J, et al. Critical Assessment of Metagenome Interpretation – a benchmark of computational metagenomics software. Nat Methods. 2017;14:1063–71.

31. Simonson AB, Servin JA, Skophammer RG, Herbold CW, Rivera MC, Lake JA. Decoding the genomic tree of life. Proc Natl Acad Sci U S A. 2005;102:6608–13.

32. Segata N, Izard J, Waldron L, Gevers D, Miropolsky L, Garrett WS, et al. Metagenomic biomarker discovery and explanation. Genome Biol. 2011;12:R60.

33. Urich T, Lanzén A, Qi J, Huson DH, Schleper C, Schuster SC. Simultaneous Assessment of Soil Microbial Community Structure and Function through Analysis of the Meta-Transcriptome. PLoS ONE. 2008;3.

34. Li H, Durbin R. Fast and accurate short read alignment with Burrows–Wheeler transform. Bioinformatics. 2009;25:1754–60.

35. Lau MCY, Harris RL, Oh Y, Yi MJ, Behmard A, Onstott TC. Taxonomic and Functional Compositions Impacted by the Quality of Metatranscriptomic Assemblies. Front Microbiol. 2018;9.

36. Griffiths-Jones S, Bateman A, Marshall M, Khanna A, Eddy SR. Rfam: an RNA family database. Nucleic Acids Res. 2003;31:439–41.

37. Nawrocki EP, Eddy SR. Infernal 1.1: 100-fold faster RNA homology searches. Bioinformatics. 2013;29:2933–5.

38. Rice P, Longden I, Bleasby A. EMBOSS: The European Molecular Biology Open Software Suite. Trends Genet. 2000;16:276–7.

39. Vaser R, Pavlović D, Šikić M. SWORD—a highly efficient protein database search. Bioinformatics. 2016;32:i680–4.

40. Love MI, Huber W, Anders S. Moderated estimation of fold change and dispersion for RNA-seq data with DESeq2. Genome Biol. 2014;15.

41. Varet H, Brillet-Guéguen L, Coppée J-Y, Dillies M-A. SARTools: A DESeq2- and EdgeR-Based R Pipeline for Comprehensive Differential Analysis of RNA-Seq Data. PLOS ONE. 2016;11:e0157022.

42. Benjamini Y, Hochberg Y. Controlling the False Discovery Rate: A Practical and Powerful Approach to Multiple Testing. J R Stat Soc Ser B Methodol. 1995;57:289–300.

43. Rho M, Tang H, Ye Y. FragGeneScan: predicting genes in short and error-prone reads. Nucleic Acids Res. 2010;38:e191.

44. Li W, Godzik A. Cd-hit: a fast program for clustering and comparing large sets of protein or nucleotide sequences. Bioinforma Oxf Engl. 2006;22:1658–9.

45. Frazee AC, Jaffe AE, Langmead B, Leek JT. Polyester: simulating RNA-seq datasets with differential transcript expression. Bioinformatics. 2015;31:2778–84.

46. Huang W, Li L, Myers JR, Marth GT. ART: a next-generation sequencing read simulator. Bioinformatics. 2012;28:593–4.

Figure1

Click here to access/download;Figure;Fig1.tiff

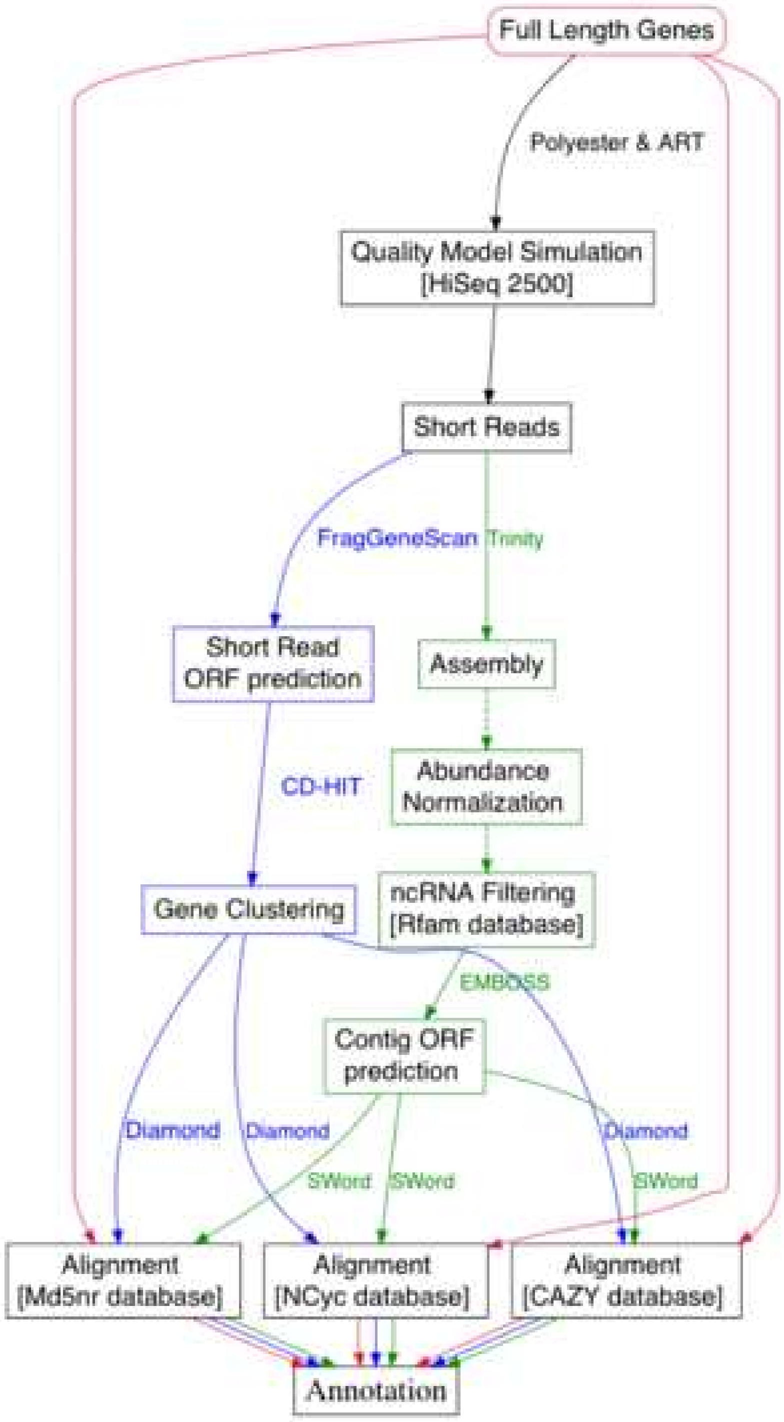

Figure2 Click here to access/download;Figure;Fig2.tiff

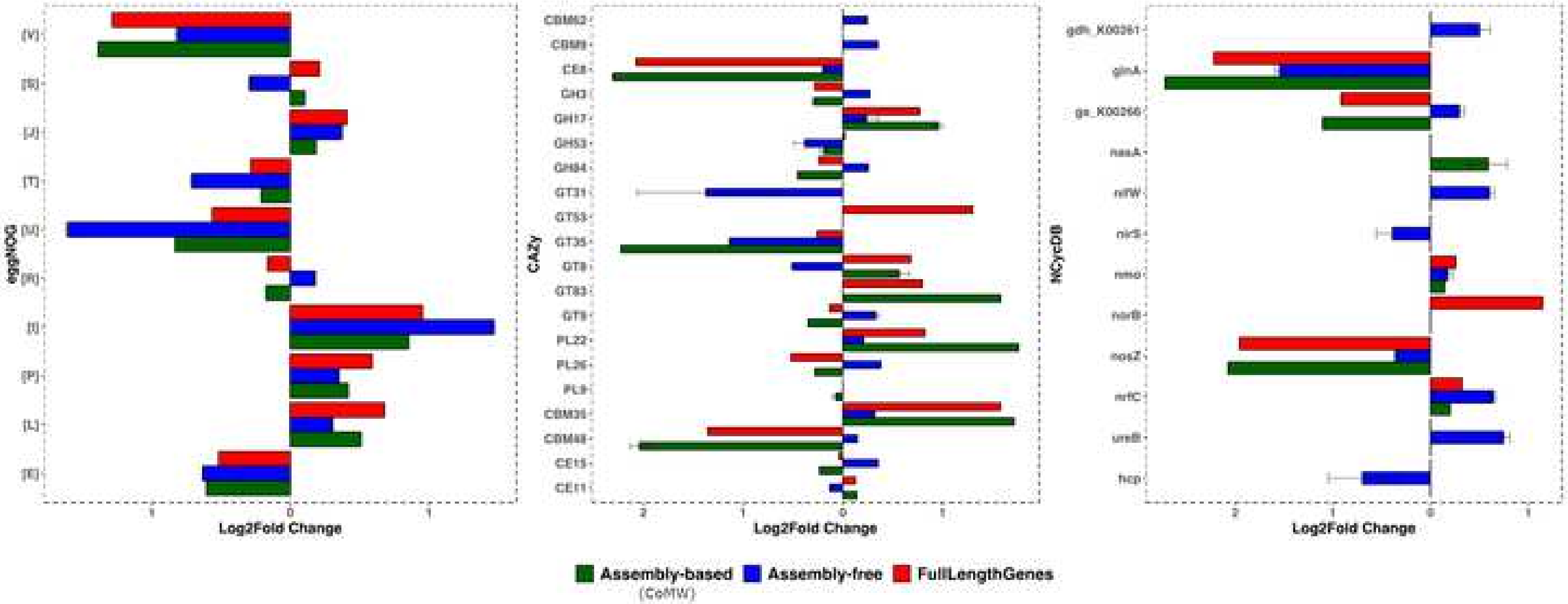

Figure3

Click here to access/download;Figure;Fig3.tiff

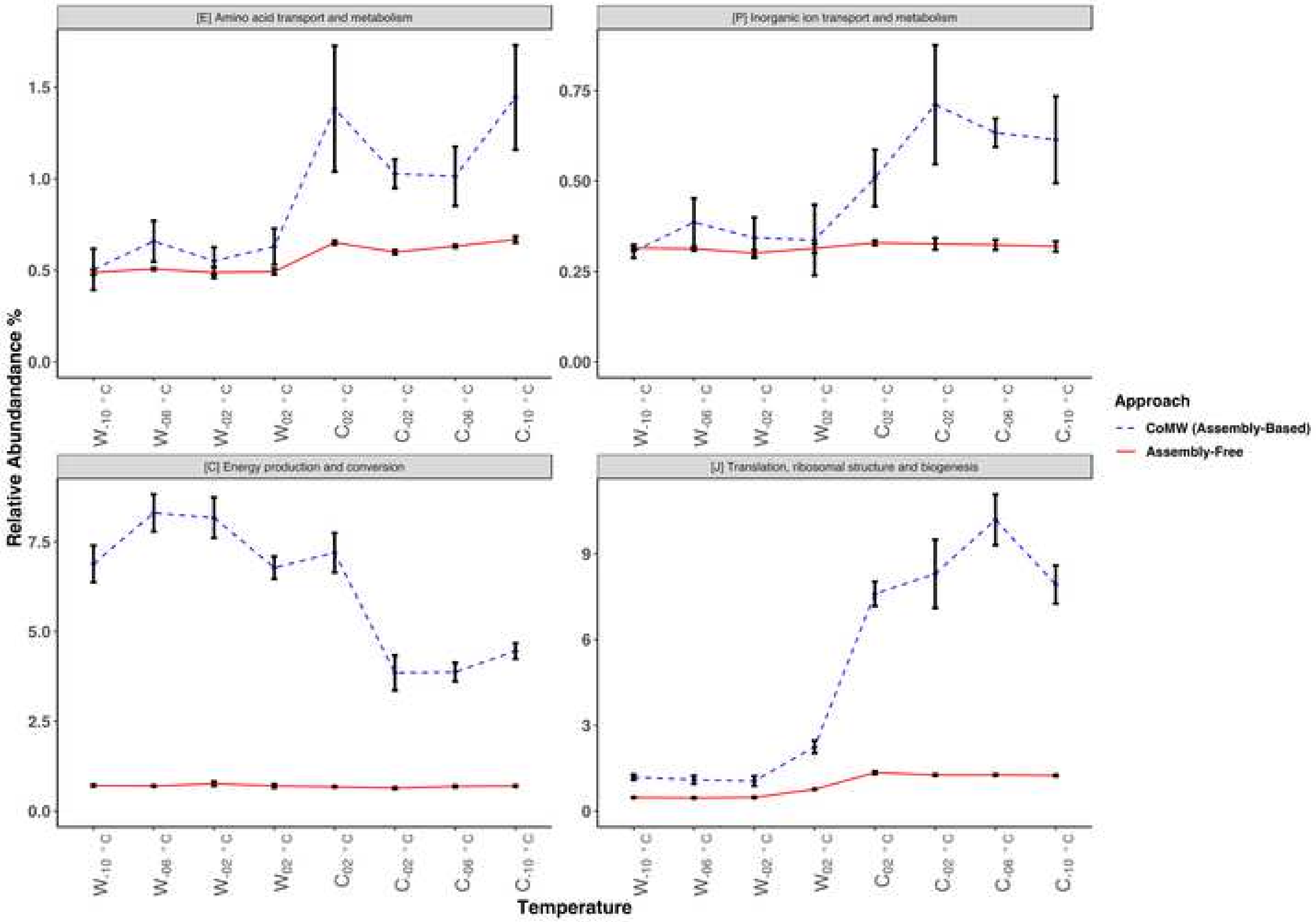

Figure4

Click here to access/download;Figure;Fig4.tiff

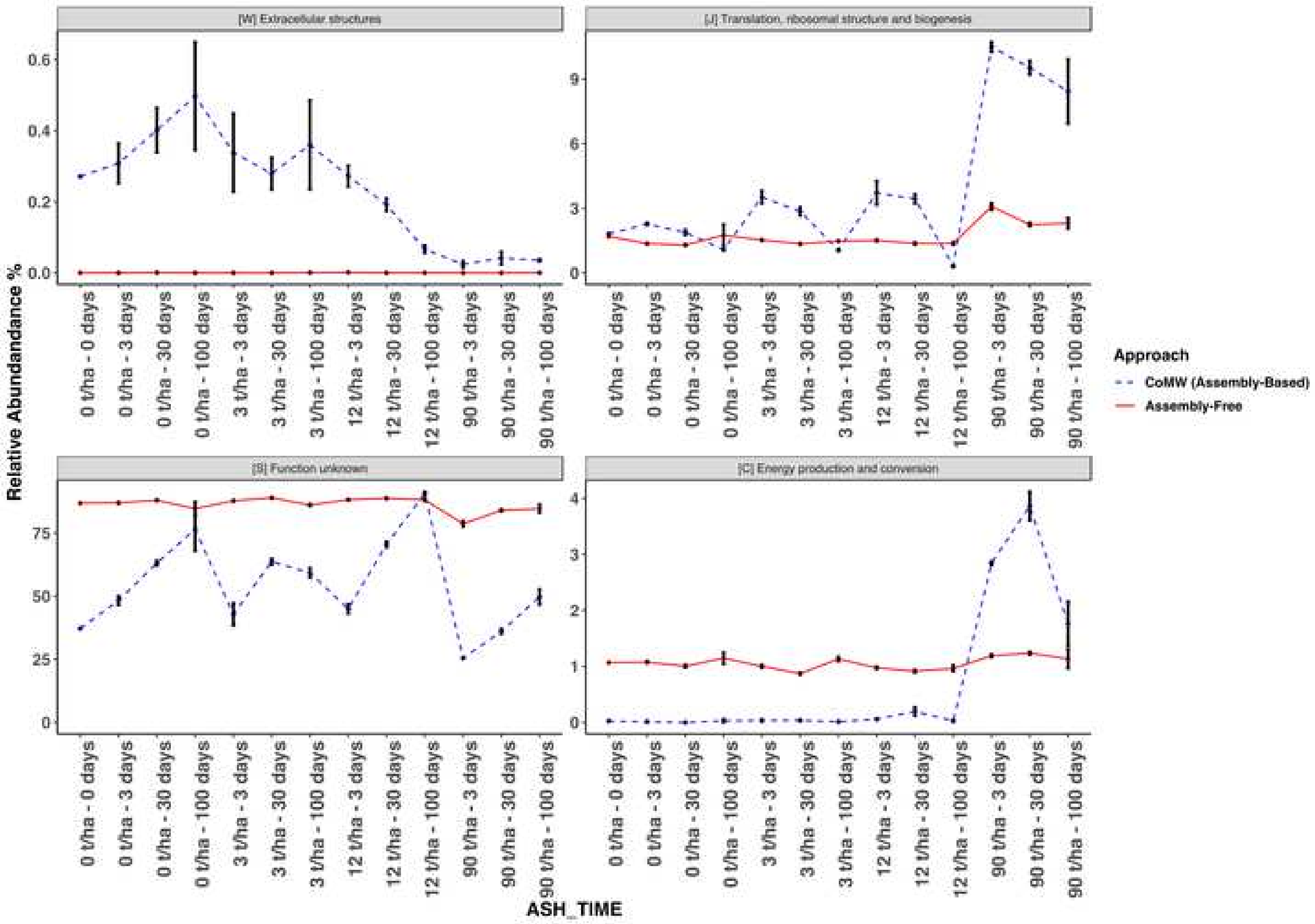

Supplementary Material File1

Click here to access/download
Supplementary Material
SupplementaryFile1_PrecisionRecall.docx

Supplementary Material File2

Click here to access/download
Supplementary Material
SupplementaryFile2_eggNOG_DEAnalysis.xlsx

Supplementary Material File3

Click here to access/download
Supplementary Material
SupplementaryFile3_CAZy_DEAnalysis.xlsx

Supplementary Material File4

Click here to access/download

Supplementary Material

SupplementaryFile4_NCyC_DEAnalysis.xlsx

Click here to access/download
Supplementary Material
Response_To_Reviewers_Comments_GigaScience.doc
x

CoverLetter_revision
Click here to access/download;Personal Cover;Anwar_CoverLetter

AARHUS UNIVERSITY
DEPARTMENT OF ENVIRONMENTAL SCIENCE

To: Editor, *GigaScience*

Re: Submission of a revised-manuscript GIGA-D-19-00009 to *GigaScience* in response to review received on 26 February 2019

We thank the editor and the reviewers for all the comments and the time and effort that have put into our submission. We believe that they provided huge guidance for improving the manuscript and reproducibility of this study and thus we have attempted to address every comment and provide responses in tabular form, please see attached. We have agreed to most of the concerns raised by the reviewers and have addressed them individually but we also have replied reasons where we feel the response is currently out of scope of the study.

In response to the major concerns that were summarized from the reviewers' comments we have spent significant efforts to firstly, make CoMW workflow easy to install and use with the anaconda configuration file provided, details of which are addressed in the response file. Secondly, in response to a healthy feedback from the reviewers we have also restructured the manuscript which we believe has improved the clarity and cohesion of the manuscript for readers of *GigaScience.*

Additionally, we have also spent considerable effort on improving data availability, reproducibility and dissemination of our results. We have made a supplementary GitHub repository as suggested by the reviewer 2 to include the scripts and parameters used by in benchmarking and generation of simulated data. As suggested we have also published CoMW as peer-reviewed compute capsule at oceancode and registered at scicrunch.org. Please see in the data availability below.

Finally, we have also made the manuscripts cited as under-review available at BioRxiv as pre-prints as asked by the editor and reviewers. This will further increase the understanding of real-world metatranscriptomes used in this manuscript and as pointed out by the Reviewer 2, detailed results and analyses of these metatranscriptomes is also available for the readers which further signifies our commitment to open and reproducible research.

Lastly, we have addressed all major and minor revision points in the manuscript and with the improvements and restructuring done we believe that the attached revised manuscript along with the Comparative Metatranscriptomics Workflow (CoMW) will be an appropriate for readers of GigaScience. We can

Environmental microbiology & biotechnology

Muhammad Zohaib Anwar
PhD Student

Date: 15 May 2019

E-mail:
mzanwar@envs.au.dk
Web:
au.dk/en/mzanwar@envs

Sender's CVR no.:
31119103

Environmental microbiology & biotechnology
Aarhus University
Frederiksborgvej 399
PO box 358
DK-4000 Roskilde
Denmark

Tel.: +45 8715 0000
Fax: +45 8715 5010
E-mail: envs@au.dk
Web: envs.au.dk/en

AARHUS
UNIVERSITY
DEPARTMENT OF ENVIRONMENTAL SCIENCE

confirm this manuscript presents material that has not previously been published and is not under consideration for publication elsewhere and all authors have seen and approved the revised version submitted.

Data availability:

- Raw sequence data generated using simulation of full-length genes were deposited in the NCBI Sequence Read Archive and are accessible through BioProject accession number PRJNA509064
- Project home page: https://github.com/anwarMZ/CoMW
- Project supplementary scripts: https://github.com/anwarMZ/CoMW_supp
- CoMW is published as a per-reviewed compute capsule at oceancode https://doi.org/10.24433/CO.1793842.v1
- Scicrunch RRID - SCR_017109

Once again, we would like to thank you for considering our manuscript in *GigaScience*. Please do not hesitate to contact us, should you have further questions.

Best regards,
on behalf of all authors

Muhammad Zohaib Anwar
