# Supplementary material for: To assemble or not to resemble—A validated Comparative Metatranscriptomics Workflow (CoMW)
Source: Gigascience. 2019 Jul 30;8(8):giz096. doi: 10.1093/gigascience/giz096 (PMC6667343; doi:10.1093/gigascience/giz096)
Supplement: giz096_Response_to_Reviewer_Comments_Original_Submission [file giz096_response_to_reviewer_comments_original_submission.pdf]

Author's Response To Reviewer Comments

Close

We thank the journal for conducting a thorough review of our manuscript, and value the thoughtful comments provided by the reviewers. Our responses to specific reviewer comments are below. We have attempted to address all comments individually and incorporated changes as needed. We have attached a response file to the file inventory that might be of help.
Comment = C, Response R

Editor:

C1: Although it is of interest, we are unable to consider it for publication in its current form. The reviewers have raised a number of points which we believe would improve the manuscript and may allow a revised version to be published in GigaScience. As a major concern the reviewers, in particular reviewer 2, highlight that many details, especially regarding the methods, are missing.

R1: We thank the editor and reviewers for their valuable feedback and thoughtful comments. We also acknowledge the missing details that were missing in manuscript and thus have subsequently made all details explicitly available in the manuscript and below in specific responses to reviewers with external links where appropriate. We also believe that now we have addressed every comment by improving the manuscript and supplementary information, please see specific answers to each comment below.

C2: They also recommend to package the tool with docker or conda, as they had some problems installing the software.

R2: Installation has been made significantly easier and reproducible using a conda environment as suggested. Now the user has to just create a conda environment with provided configuration file that includes all framework tools, libraries, dependencies and third-party tools along with optimized versions. A small bash script is also added which will download the databases directly from the FTP server https://mzacomw.au.dk hosted by Aarhus University.
> conda env create -f CoMW.yml
> source activate CoMW
> bash install.sh
A detailed answer is given to reviewers below. Also, an updated Readme and Manual are made available at the Github repository. See
https://github.com/anwarMZ/CoMW

C3: Please improve the description of methods and parameters.

R3: We have now also made a configuration file available that is used for the installation. This file also contains the versions of the libraries, channels, third party tools and versions of framework tools (e.g. python, R). Please see https://github.com/anwarMZ/CoMW/blob/master/CoMW.yml.Parameters used are also now added in to the comments here or manuscript depending upon suitability.

C4: Please also share all supporting scripts and code (this can be done via GitHub, or via our repository GigaDB).

R4: All scripts are shared in the Github repository– https://github.com/anwarMZ/CoMW Script used for simulating short reads from mock communities used is also now referenced in the text. The script used was made available by the Metatrans authors: https://media.nature.com/original/nature-assets/srep/2016/160523/srep26447/extref/srep26447-s1.doc Additional scripts for removing a functional subsystem from the database and SARTools differential expression analysis and parameters for running metatrans (assembly-free) approach are now added to separate Github-repo as suggested by the 2nd reviewer - https://github.com/anwarMZ/CoMW_supp

C5: Submit code and data to code ocean as a “computational capsule” (https://codeocean.com/). Code

ocean assigns DOIs, which you can cite in your GigaScience manuscript.

R5: As suggested, CoMW is now published as a peer-reviewed computational capsule at codeocean [1] and can be accessed through https://doi.org/10.24433/CO.1793842.v1

C6: Please also clarify the origin of the "real world datasets" and the papers referred to as being "under review" – is there a preprint that can be cited in the paper, if these are not yet published.

R6: We have now made both manuscripts available at BioRxiv. Authors MZA and CSJ are co-authors on both studies, whereas AL is co-author on one study led by TBA. Both the manuscripts contain experimental setup, details about the datasets and implementation of CoMW for the analysis. 1. Schostag, Morten Dencker, Muhammad Zohaib Anwar, Carsten Suhr Jacobsen, Catherine Larose, Timothy M. Vogel, Lorrie Maccario, Samuel Jacquiod, Samuel Faucherre, and Anders Priemé. "Transcriptomic responses to warming and cooling of an Arctic tundra soil microbiome." bioRxiv (2019): 599233. 2. Bang-Andreasen, Toke, Muhammad Zohaib Anwar, Anders Lanźen, Rasmus Kjøller, Regin Rønn, Flemming Ekelund, and Carsten Suhr Jacobsen. "Total RNA-sequencing reveals multi-level microbial community changes and functional responses to wood ash application in agricultural and forest soil." bioRxiv (2019): 621557.

C7: Both reviewers also feel that the overall structure of the manuscript needs improvement. Reviewer 1 got the impression that it reads almost as if being two separate manuscripts, with two separate aims. I recommend that you should consider this remark to improve the clarity of the paper and its message, how it all ties together.

R7: We appreciate the comments of both reviewers that remarked about this. In line with both, but more specifically as suggested by Reviewer 2, we have restructured the paper significantly to make it easier to read and to make the Methods section clearer. We first describe the overall implementation of CoMW, described in detail in Results, then the alternative approach, followed by the benchmarking and comparison between the two, using several reference databases for functional annotation, for both a simulated and a real-world dataset. We believe that now the manuscript with improved structure and previously missing details sync well for readers of GigaScience.

C8: In addition, please register any new software application in the SciCrunch.org database to receive a RRID (Research Resource Identification Initiative ID) number, and include this in your manuscript. This will facilitate tracking, reproducibility and re-use of your tool.

R8: CoMW is now registered at SciCrunch.org with RRID – SCR_017109. Also updated in the manuscript as per appropriate.

Reviewer 1:

C1: The authors use a previously published simulated dataset as well as two real-world metaT datasets from two environments. In recent years more and more microbiome studies are coming out using metaT instead of metagenomics (metaG) to describe active genes and species within microbial communities. Therefore, it is right time to benchmark different methods and analysis pipelines for metaT studies.

R1: We appreciate the acknowledgment of our work and constructive feedback. We have made improvements as suggested by the reviewer where necessary. Please see below for more detailed response to each comment.

C2: The main problem that I have with the paper is that it's actually two papers, one paper on testing. Different strategies for metaT analysis and one paper on a new analysis workflow for metaT analysis. For the second part, the CoMW pipeline, a more detailed description would be good, especially since it contains steps that are not necessary for the benchmark part before, e.g. the noncoding analysis. In general, I would strongly recommend to focus only on one of both parts and restructure the paper accordingly or have two separate papers.

R2: We appreciate the feedback and we have changed the overall structure of the paper to first of all

present CoMW, then benchmark it using different sensitivity settings, and compare it to an assembly-free approach.

C3: The benchmarking part has the weakness that it is not really a comparison of existing tools or pipelines but of two strategies. The authors defined for each strategy the workflow they considered as the best, but obviously each strategy could be implemented with different tools and steps, and e.g. for the assembly-based strategy different tools and preprocessing steps can have a major impact for the resulting assemblies and therefore for all downstream analysis, but this is not really discussed here. For each strategy specific tools and parameters have been used. It should be clearer be stated why they have chosen, e.g. there are newer metaT assemblers than trinity, e.g. rnaspades and megahit, for the proposed benchmark I would at least want to see two or more implementations for each strategy.

R3: We agree with this comment, and thus we have restructured the manuscript significantly, as described above It should now be clearer that the idea and scope of this study is focused on elucidating the difference of using a typical assembly-free approach, to a typical assembly-based approach, specifically our implementation of CoMW, using the state-of-the-art tools for each strategy and evaluating them as holistically as possible. Additionally, several benchmarking studies have already been done for certain steps included in CoMW eg. Zhao et al. & Celaj et al. [2,3]. Thus, we have used Trinity as our assembler based on these benchmarking results. Similarly, for assembly-free approach we have used DIAMOND as short read mapper which has been thoroughly compared to its similar tools such as BLASTX and RAPSearch2 [4]. Megahit is metagenomic assembler and we believe should not be used for metatranscriptomics. However, one of the reasons to make CoMW modular was to make the users able to use an alternative tool (if preferred due to any reason) at certain step. For example, rnaspades can be used as an assembler and skip the step of assembling through CoMW.

C4: In the introduction examples from human gut studies are missing.

R4: We agree with the comment and have subsequently added two example studies from Gosalbes et al., and Abu-Ali et al. [5], [6] in the introduction of the manuscript (please refer to L55)

C5:A critical review of prior benchmarks in metaG and/or metaT should be given, e.g. CAMI should be mentioned (https://www.ncbi.nlm.nih.gov/pubmed/28967888) and the authors should discuss if the results for metaG and metaT can be compared and what are the specific problems of benchmarking metaT workflows.

R5: We now refer to CAMI [7] in the Discussion (L264 in manuscript). However, it is mainly benchmarking tools for certain steps in metagenomics analyses.

C6 Why those three databases have been chosen, why not others.

R6: The databases were selected in order to include a representative selection of three different degrees of specialization, on a range from a more inclusive database with wide coverage (universality) and low degree of expert curation, to a smaller, highly curated database, with more narrow coverage. The three databases were also chosen because they are among the most widely used representing these categories. This is now also added to the introduction in manuscript. Please see L86-92.

C7:No discussion of other workflows available like anvi'o or IMP that could do similar things

R7: Both IMP and CoMW are modular can also be used together based on the preferred choice of the user. We have now clarified this in the Discussion and cited IMP. Please see L67 & from L266 in manuscript. Anvio is based on “maxBin” [8] which is a binning method mainly used for recovering individual genomes from metagenomes which is out of the scope of this study. IMP has some overlaps with our study, and some significant differences, which is now mentioned in the manuscript as well.

C8: The two real world datasets are only referenced as 'under review', either they should be submitted as preprint or added as supplement to the reviewers. Also, it is not clear if the authors are part of those studies.

R8: We agree and have now made both the manuscripts available at BioRxiv and have cited them in the revised manuscript. Please refer comment 6 to editor for more details.

C9: For the simulated dataset it is not totally clear how it was generated. Did the authors use only full-

length genes or did they also include the non-coding parts of fully transcribed operon sequences as you would find expect them in real metaT datasets. This will have a big impact on the assembly quality, mapping and expression analysis.

R9: We used the full-length genes provided by the Martinez et al. [9]. (only coding part and not full operons). This is now explained in the manuscript (L368 onwards)

C10: How are the subsystems that were removed during the benchmarking procedure were chosen?

R10: Subsystems removed from each category were chosen on random to keep it unbiased removal. This is now updated in the manuscript along with reference to now updated script for the method. (L153 in manuscript)

C11: How the optimal alignment and mapping, e.g. BTS parameters were chosen for alignment and mapping?

R11: Both alignment and mapping were done on a range of parameters as described in Methods and specified in detail in supplementary file 1 to evaluate the performance for different (user selectable) sensitivity thresholds and avoid pre-selecting a certain threshold value. Assembled contigs were mapped from e-value 1E-1 to 1E-15. Similarly diamond alignment using short reads was tested on 15 BTS scores from BTS 10 to 150.

C12: The software is not straightforward to install, especially for non-bioinformaticians a real reproducible version using docker or conda would be highly appreciated. Also implementing the workflow as set of scripts should be transformed into a more formal structure using a workflow description like snakemake

R12: The software has now made significantly easier by using it in conda env and can be installed relatively easy in a separate conda container using the configuration file https://github.com/anwarMZ/CoMW/blob/master/CoMW.yml as described in the updated manuscript and attached CoMW manual. Now the user has to just create a conda env with provided configuration file that includes all framework tools, libraries, dependencies and third-party tools along with optimized versions and Run installation file

```
> conda env create -f configuration.yml
> source activate CoMW
> bash install.sh
```

Moreover, the aim of the workflow is to be modular and possible to adapt to alternative tools and databases. Though we appreciate the ease of use of Snakemake, especially for a user without expertise in python, we do not feel that this would allow for sufficient modularity. Instead CoMW is targeted to users with basic bioinformatics expertise comfortable to work in the command line, or for more advanced users with python expertise to allow changes in configuration, databases and programs used.

C13: Title is way too long and should be changed according the possible restructuring of the manuscript

R13: Suggestion well taken and since we have restructured the manuscript according to feedback we have also restructured the title a bit.

C14: Line 193: wrong figure seems to be referenced. Figure 3 should be Figure 2.
R14: We have changed the manuscript accordingly
C15: In paragraph 2.1.3 the reference to Figure 3 (not 6) is missing. Figures 3 and 4: colours not explained and unclear.
R15: We have corrected the reference to the figure and have improved the figure and caption.
C16: Numbers zero to nine should be given as words.
R16: We have corrected this style error throughout the manuscript.
C17: 132: Assembly-based should be lower case
R17: Correction well taken and we have updated for consistency all throughout the manuscript accordingly.
C18: Table 2: not clear when lower and upper case is used and why
R18: We apologize the unclear annotation and have changed incorrect uppercase words
C19: 161: Nitrogen upper case
R19: We have updated for consistency all throughout the manuscript accordingly.

C20: SARTOOLs has a different reference https://www.ncbi.nlm.nih.gov/pmc/articles/PMC4900645/
R20: Thank you for pointing out. During in-text citation, one of the citations is mis-cited. Corrected now throughout
C21: Ref 21, reference style is wrong
R21: Correction well taken and references are consistent now
C22: Not clear how to handle references that are currently under review, should be put on bioarxiv e.g. reference 17 and 18
R22: Please see comment no 8 and comment 6 to editor. We have now made both manuscripts available on BioRxiv
C23: Description of required software versions is missing, e.g. which Java and Python version
R23: The missing information of versions is now part of configuration file along with all libraries and dependencies. See configuration file at https://github.com/anwarMZ/CoMW/blob/master/CoMW.yml

Reviewer 2:

C1: My primary concern with the paper is that as it stands I am not sure that anyone would be able to replicate these analyses. Broadly, I think that the methods section is confusingly structured. I found myself skipping back and forth to try to figure out what you had done. The methods section should be restructured and organized to follow the flow of the analysis presented in the results. I suggest starting with a section describing how the mock data sets were created, then describe the use of the real-world meta-transcriptomes (which aren't well covered in the materials and methods), and then describe the two pipelines that were used including your novel CoMW.

R1: We appreciate the comments of the two reviewers that both remarked about this. In line with both, but more specifically as suggested by Reviewer 2, we have restructured the paper to make it easier to read and to make the Methods section clearer. We first describe the overall implementation of COMW, described in detail in Results, then the alternative approach, followed by the benchmarking and comparison between the two, using several reference databases for functional annotation, for both a simulated and a real-world dataset.

C2: Throughout the materials and methods, you should make sure that sufficient detail on all programs/tools used are included - meaning that the version of all software products should be included (e.g. DESeq2, Trinity, Diamond, etc.). Additionally, all parameters used should be included.

R2: We agree and have now made a configuration file available that is used for the installation. This file also contains the versions of the libraries, channels, third party tools and versions of framework tools (e.g. python, R). Please see this -
https://github.com/anwarMZ/CoMW/blob/master/CoMW.yml Parameters used are also now added in manuscript depending upon suitability.

C3: Notably the description of the removal of "functional subsystems" was missing from the methods. How was this done?

R3: Subsystems to remove from each category were chosen on random to keep it unbiased removal.
https://mzacomw.au.dk/eggNOG.md52id2ont.zip
A file provided with eggNOG annotations was used to filter all sequences using script provided in supplementary repository,
https://github.com/anwarMZ/CoMW_supp/blob/master/remove_m5nr_subsystem.py
from database file by tracking the md5sum of Orthologous groups belonging to a certain functional subsystem. The manuscript has been updated to describe this in detail.

C4: I also do not think sufficient information was provided regarding how the mock RNA libraries were generated.

R4: These libraries were generated by Martinez et al. [9] and are described in more detail in the cited paper. Briefly, a subsample of 1000 genes from five different microorganisms was selected randomly, injected into Polyester to simulate short reads for two groups with different transcription levels, each

containing 50 simulated samples.

C5: Moreover, the authors should clarify how the differential expression analysis was done-it appears to be absent from the paper.

R5: We used the template script provided by the SARTools for DESeq2 analysis. https://github.com/PF2-pasteur-fr/SARTools/blob/master/template_script_DESeq2.r
now updated in the manuscript as well. L354 in manuscript

C6: Broadly speaking, I think it would be useful to have another github repo or a folder on the current github that contains all the supplementary scripts that were used for this publication (e.g. the Polyester scripts used, DE expression, etc.)

R6: All scripts are shared in the Github repository– https://github.com/anwarMZ/CoMW

One additional script used for simulating short reads from mock communities used is already made available by the Metatrans authors:
https://media.nature.com/original/nature-assets/srep/2016/160523/srep26447/extref/srep26447-s1.doc

C7: The pipeline is great as written-- but I do wonder why it is written in python and not implemented in some sort of workflow language/system (e.g pydoit, snakemake, etc.). If the goal is reproducibility, conda environments (or the like) should be included to facilitate the use of the same software versions throughout the workflow. Additionally, it would appear that the current scripts within CoMW are written to work with specific versions of each of the software packages covered (e.g. Trinity). What version were these scripts tested with? I tried running and ran into issues with Trinity that I think were due to the version I was trying to use. I suggest making a conda environment file (https://conda.io/projects/conda/en/latest/user-guide/tasks/manage-environments.html) that can be included in the Github repo to detail what version of each of the software programs should be used for the pipeline.

R8: The software has now made significantly easier by using it in conda environment and can be installed relatively easy in a separate conda container using the configuration file https://github.com/anwarMZ/CoMW/blob/master/CoMW.yml as described in the updated manuscript and attached CoMW manual. Now the user has to just create a conda environment with provided configuration file that includes all framework tools, libraries, dependencies and third-party tools along with optimized versions and Run installation file

```
> conda env create -f CoMW.yml
> source activate CoMW
> bash install.sh
```

Moreover, the aim of the workflow is to be modular and possible to adapt to alternative tools and databases. Though we appreciate the ease of use of Snakemake, especially for a user without expertise in python, we do not feel that this would allow for sufficient modularity. Instead CoMW is targeted to users with basic bioinformatics expertise comfortable to work in the command line, or for more advanced users with python expertise to allow changes in configuration, databases and programs used.

C8: I am perhaps missing it-- but it would appear that no quality trimming of the raw short reads was used in this study. This is generally accepted as best practice. Please explain its omission from both pipelines in the text. More broadly, I think it should be added to the CoMW workflow.

R8: The real-world dataset was trimmed as described in the cited preprints [10,11] . However, we would also like to mention in general that quality trimming is not always desirable for alignment to a protein database when only doing so for the purpose of annotation, since it can decrease sensitivity by removing informative, though erroneous bases [12]. For assembly however, a gentle trimming based on the above paper is already included in Trinity by default.

C9: The comparisons made in the paper focus primarily upon functional analysis (e.g. the identification of functional gene sets), and the simulated data set that is used is not well-suited to taxonomic data. The comparisons made in the paper are interesting and valid-- but I think that the authors should make clear that they are only really testing functional annotation and not the resolution of taxonomic

annotation.

R9: We agree that the simulated datasets are not suitable for taxonomic annotation and that taxonomic annotation is outside of the scope of this paper. We have now clarified this in a paragraph regarding taxonomic annotation in the Discussion. L295 in manuscript

C10: Looking at Table 1, I am inclined to say that eggNOG does pretty well in general with or without assembly and that the other databases perform less well. Moreover, that other databases seem to be more depend upon assembly vs. not. I am not sure that this shows a clear-cut win for assembly. Additionally, how much variation might be driven by mapping approach used (Diamond) as compared to some other short read mapper/blasting.

R10: We have improved the caption of the Table 1. However, our interpretation of these results differs from the reviewer's; annotations using eggNOG are not completely comparable or similar between both approaches, since the precision drops significantly at medium and higher threshold of confidence by adding excessive false positives. Whereas, for the assembly-based approach or CoMW the precision doesn't drop at all even at a very high threshold. In Table 1 we have only added the best value for each database based on the F-scores. Please see Supplementary table 1 to have a complete view. The false positives remain less than 1% compared to 15% using assembly-free approach. Also, if one considers another mapper's than Diamond they are less or equally sensitive as Diamond. Using more sensitive tools such as SWORD without denovo assembly is not possible given the computational restrain especially for large metaT datasets.

C11: One approach used frequently in the analysis of microbial communities dominated by prokaryotes is to sequence both the metaG and metaT of the community, assemble the metaG, and then map the metaT to the assembled metaG or binned genomes from the metaG (e.g. https://www.nature.com/articles/s41396-017-0041-5). It seems worthwhile to discuss how your approach differs from these approaches.

R11: We agree that this is possible and might be good idea in many cases. However, with CoMW, we aim to develop a workflow applicable to pure metatranscriptomics studies, providing a resource for de novo assembly and functional analysis. This is especially relevant for environments such as soil where the high diversity and amount of metabolically active organisms means that the sequencing depth needed for metaG assembly becomes economically unfeasible for many studies, especially when, as here, microeukaryotes are also targeted.

C12: Line 69: Without a meta-analysis I am not sure that this can be stated-citation 11 only lists 5 studies that take that approach in a fairly limited subset of studies. I would remove this statement unless you have solid numbers on how many studies use assembly vs mapping approaches? Also, you should qualify your extrapolation that it is "because of less computational expense." You don't know people's rationale for choosing one pipeline over another.

R12: We agree with the reviewer's comment and have removed the statement in introduction.

C13: Line 70: Some discussion of the CAMI metagenomic comparisons (https://data.cami-challenge.org/) seems justified here. They did extensive comparisons of assembly approaches as well as direct taxonomic analysis of short reads. The CAMI parallels your investigation of functional gene annotation nicely.
R13: We agree and now mention CAMI in manuscript L264, though as mentioned above, CAMI has a different focus and its benchmarks are mostly out of the scope of CoMW.

C14: Line 99: It would be helpful to the reader to directly state what the 'full-length genes' are. I am assuming that they are from the genome data that were used to generate the simulated data. The language should again be clarified in Line 110. One line 110 I am not sure what "full length genes" are being referred to. Perhaps changing the term used to "gold standard" or the like would help the reader follow the logical progression better?

R14: We have now revised the language in the lines as suggested, but prefer to keep the term "full length genes". It is now better explained to improve clarity.

C15: Line 112: Define BTS score.
R15: BTS = “bit score”. This is now revised.

C16: Line 116: What numerical cutoffs were used for each of the Low, Medium, and High thresholds? Are the parameters detailed somewhere? I looked at the supplemental tables-but couldn't figure out which if any columns contained these values. Apologies if I missed them.

R16: They are specified as one column in each supplementary table in supplementary file 1. However, for more clarity, we have also mentioned here

Threshold Category assembly-based assembly-free
Low – TL 1E-1 – 1E-5 BTS 10 – 50
Medium – TM 1E-6 – 1E-10 BTS 60 – 100
High – TH 1E-11 – 1E-15 BTS 110 – 150

C17: Line 132: Watch capitalization of your two approaches throughout the paper ("Assembly-based and assembly-free approaches"). You change around quite a bit whether or not you capitalize.
R17: We have updated for consistency all throughout the manuscript accordingly.

C18: Line 152: As with line 116, what were the parameters used in the 15 "various confidence thresholds". I think it would be helpful to add a supplemental table that specifies the confidence thresholds used. R18: The parameters for alignment were consistent throughout the 15 confidence thresholds except the confidence threshold itself in both approaches. For the cut-off used or for the definition of the thresholds see response to comment # 16 above.

C19: Line 178: It is unclear to me how "full length" gene alignments were used for quantification? Was this based on mapping the simulated short reads against the original full-length genes that were annotated with eggNOG? Clarify in the methods or here.
R19: Yes exactly, simulated short reads were mapped back to the full-length genes (annotated using all three databases) for quantification and comparing the quantification using “assembly-based” (CoMW) and “assembly-free” methods. We have also updated in the methods for clarity.

C20: Line 204: What approach was used by Schostag? Was it assembly free? What was found by the assembly free approach?
R20: We have used assembly-based approach (CoMW) in Schostag et al. [10] as both MZA and CSJ are co-authors on the study. However, to elucidate the impact of the choice, we used “assembly-free” approach too and compared the results. Which is shown in figure 3 in manuscript

C21: Line 209: There is no Figure 6.
R21: Updated accordingly

C22: Line 209: There is no Figure 7.
R22: Updated accordingly

C23: Line 243: What was the estimated chimeric sequence frequency from your analysis of the mock datasets? What about false contigs? I would be surprised if they were terribly high as the mock data set didn't contain any species that had high average nucleotide identity.

R23: Simulated data didn’t have any chimeric sequences since Polyester does not simulate chimeric reads but the real data might have chimeric sequences based upon various things such as the de novo assembler used or environment under study or the dominating species. However, these contigs can be identified and filtered based on their abundance using the optional script provided filter_contigs_by_abundance.py where the user can identify a minimum threshold as a contig to not be considered as false contig based on its relative abundance to contigs pool.

C24: Line 282: Sure, I agree but do you have numbers to support this or a citation?
R24: We don’t have the exact numbers to support the argument however this review [13] of metaG, metaT and metaP approaches for microbiome analysis had a similar view. They mentioned that most of the metaT analysed studies have a custom/ad-hoc built analysis pipeline which can be due to many reasons (Computational expense, lack of computational experience, mRNA quality, databases of interest

etc.). We have accordingly added the citation to the argument.

C25: Line 283: What are "these pipelines"? Metatranscriptomic pipelines? Also, I am not sure if you have the data to say that the majority of analyses use assembly-free approaches.
R25: Agreed that we don’t have a numerical data to confirm if the majority is using assembly-free approach so subsequently we have removed the statement.

C26: Line 324: Can you provide the parameters used in the various steps of the Metatrans pipeline? Also, do you have the scripts used to run the Metatrans pipeline? If so, they should be published somewhere (Supplemental text or GitHub repo for the paper).

R26: Metatrans package was downloaded and used from http://www.metatrans.org/ . We cited their manuscript [9] where they had mentioned the availability in abstract. The parameters for each part of the workflow are now made available at
https://github.com/anwarMZ/CoMW_supp/blob/master/Metatrans_version_parameters.yml

C27: Line 336: Please provide the scripts used to generate the short reads.
R27: The script used was also available in supplementary material of Martinez et al. [9] direct link - https://media.nature.com/original/nature-assets/srep/2016/160523/srep26447/extref/srep26447-s1.doc
This script uses Polyester to simulate 100 samples in two groups (50 each) with varying expression.

C28: Line 341: Please clarify what "resulted in a count table and short reads with 2395 genes to add the impact of sequencing coverage" means. It is unclear to me what was produced and why only 2395 genes were recovered if 4943 were generated.
C28: Read simulator mimics the coverage bias and thus some genes were removed

C29: Lines 342: What does knocking out genes mean in the context of short reads? How was this done? Please publish the scripts used-- I couldn't find them in the GitHub repository.

R29: Knocking out is referring to the process of removing the abundance of randomly selected 5% of genes from the simulated data. 5% genes in the table were randomly set as 0 in 5 samples in the countable in order to mimic real data. Following that BioPython was used to remove reads belonging to that genes similarly from the simulated sequence files. We have now added this script to the supplementary repository. https://github.com/anwarMZ/CoMW_supp

C30: Line 353: Include the accession number for your simulated data set in the text as well as at the end of the paper.

R30: The information was added to the manuscript in Availability of Supporting data and Materials - Raw sequence data generated using simulation of full-length genes were deposited in the NCBI Sequence Read Archive and are accessible through BioProject accession number PRJNA509064. These reads are under the simulated Mock Communities classification of SRA

C31: Figure 1: You have "custom python scripts added in CoMW" at the bottom-but I can't see anything highlighted with that in the figure. It might be obscured because the figure is low resolution.
R31: We have improved the resolution of image. However, we don’t think the “custom python scripts added in CoMW” are needed after the restructuring of manuscript and also now CoMW is easier to use for all steps after improving installation.

C32: Figure 2: Figure 2 is never referenced in the text. Also, I can't read this figure well-the text is very small and the resolution is not good. Generally, instead of plotting Log2Fold change I wonder if it would be more powerful to plot the difference relative to the Full-length gene analysis.
R32: We have improved the figure resolution and structure. However, we believe that since we want to compare both methods in relation to full-length genes and number of reads in both methods are not directly comparable, we use Log2Fold change instead of relative abundance of a transcript.

C33: Table 2: All tables should be able to stand alone with their caption without the text of the paper. This table is lacking some key information. What is the percentage column? What are the numbers (the number of orthologs retrieved with each method?) Label the letters-- specify what they are/where they are from. Check consistency in capitalization in titles. Specify that this is for eggNog? I also wonder if it would be better to report Recall/Precision values for the two different approaches as you did in Table 1-

as it is a bit strange to recover more values in the assembly-based approach compared to the full-length genes but then not penalize what should be false discover.

R33: We have improved the readability of caption and tables throughout. For table 2 specifically, yes, the numbers show the unique homologs retrieved in Full length-genes and in both assembly-based and assembly-free methods. The letters were labelled in the text before, however as you correctly pointed out we have also added the full name of the letters in the table. We have also corrected the capitalization and caption. As suggested we have replaced the table 2 with Recall/Precision values in order to keep the consistency and better understanding. As pointed out rightly, the values were misleading the fact that the False positives were not reported which is now corrected.

C34: Figures 3 and 4: The figure legends require more information: such as what is being plotted-relative abundance calculated how? Relative to all transcripts or relative to the study? It might also be interesting to add the relative abundance values that are reported in the original papers. These figures are interesting as the dynamic range of the data recovered is so different between the two different approaches. What do you think is the origin of this difference? Lack of mapping in the assembly free? Or spurious mapping of reads in the assembly free? It might be nice to add some more discussion of this in the text.

R34: Relative abundance of the functional subsystems in Figure 3 and 4 were calculated for each sample using both assembly-free and assembly-based methods. We have also updated the captions as per the reviewer has rightly pointed. Moreover, now that both manuscripts are made available at BioRxiv along with detailed supplementary methods we believe that the values can be seen for each dataset from the manuscripts. We have not added more information in order to keep clarity. We believe that our findings support our arguments that the main reason for the difference between the assembly-based and assembly-free approaches is the False positives produced by the assembly-free method. We saw that with simulated datasets it produced upto 15% False positives even with eggNOG databases which is inclusive and generic.

References:
1. Anwar MZ, Lanzen A, Bang-Andreasen T, Jacobsen CS. Comparative Metatranscriptomic Workflow (CoMW) [source code]. codeocean; 2019. Available from: https://doi.org/10.24433/CO.1793842.v1
2. Zhao Q-Y, Wang Y, Kong Y-M, Luo D, Li X, Hao P. Optimizing de novo transcriptome assembly from short-read RNA-Seq data: a comparative study. BMC Bioinformatics. 2011;12:S2.
3. Celaj A, Markle J, Danska J, Parkinson J. Comparison of assembly algorithms for improving rate of metatranscriptomic functional annotation. Microbiome. 2014;2:39.
4. Buchfink B, Xie C, Huson DH. Fast and sensitive protein alignment using DIAMOND. Nat Methods. 2015;12:59–60.
5. Gosalbes MJ, Durbán A, Pignatelli M, Abellan JJ, Jiménez-Hernández N, Pérez-Cobas AE, et al. Metatranscriptomic Approach to Analyze the Functional Human Gut Microbiota. PLOS ONE. 2011;6:e17447.
6. Abu-Ali GS, Mehta RS, Lloyd-Price J, Mallick H, Branck T, Ivey KL, et al. Metatranscriptome of human faecal microbial communities in a cohort of adult men. Nat Microbiol. 2018;3:356.
7. Sczyrba A, Hofmann P, Belmann P, Koslicki D, Janssen S, Dröge J, et al. Critical Assessment of Metagenome Interpretation – a benchmark of computational metagenomics software. Nat Methods. 2017;14:1063–71.
8. Wu Y-W, Tang Y-H, Tringe SG, Simmons BA, Singer SW. MaxBin: an automated binning method to recover individual genomes from metagenomes using an expectation-maximization algorithm. Microbiome. 2014;2:26.
9. Martinez X, Pozuelo M, Pascal V, Campos D, Gut I, Gut M, et al. MetaTrans: an open-source pipeline for metatranscriptomics. Sci Rep. 2016;6:26447.
10. Schostag MD, Anwar MZ, Jacobsen CS, Larose C, Vogel TM, Maccario L, et al. Transcriptomic responses to warming and cooling of an Arctic tundra soil microbiome. bioRxiv. 2019;599233.
11. Bang-Andreasen T, Anwar MZ, Lanźen A, Kjøller R, Rønn R, Ekelund F, et al. Total RNA-sequencing reveals multi-level microbial community changes and functional responses to wood ash application in agricultural and forest soil. bioRxiv. 2019;621557.
12. MacManes MD. On the optimal trimming of high-throughput mRNA sequence data. Front Genet [Internet]. 2014 [cited 2019 Apr 2];5. Available from: https://www.frontiersin.org/articles/10.3389/fgene.2014.00013/full
13. Aguiar-Pulido V, Huang W, Suarez-Ulloa V, Cickovski T, Mathee K, Narasimhan G. Metagenomics,

Metatranscriptomics, and Metabolomics Approaches for Microbiome Analysis: Supplementary Issue: Bioinformatics Methods and Applications for Big Metagenomics Data. Evol Bioinforma. 2016;12s1:EBO.S36436.

Close
