# Supplementary material for: To assemble or not to resemble—A validated Comparative Metatranscriptomics Workflow (CoMW)
Source: Gigascience. 2019 Jul 30;8(8):giz096. doi: 10.1093/gigascience/giz096 (PMC6667343; doi:10.1093/gigascience/giz096)
Supplement: giz096_Response_to_Reviewer_Comments_Revision_1 [file giz096_response_to_reviewer_comments_revision_1.pdf]

Author's Response To Reviewer Comments

Close

As asked by the editor, I have added the reference to the GigaDB dataset attached to the publication and cited it in text

Close
