# Supplementary material for: To assemble or not to resemble—A validated Comparative Metatranscriptomics Workflow (CoMW)
Source: Gigascience. 2019 Jul 30;8(8):giz096. doi: 10.1093/gigascience/giz096 (PMC6667343; doi:10.1093/gigascience/giz096)
Supplement: giz096_Reviewer_1_Report_Original_Submission — Patrick May -- 2/10/2019 Reviewed [file giz096_reviewer_1_report_original_submission.pdf]

Reviewer Report

Title: To assemble or not to resemble â€“ A validated Comparative Metatranscriptomics Workflow (CoMW)

Version: Original Submission **Date:** 2/10/2019

Reviewer name: Patrick May

Reviewer Comments to Author:

The manuscript "To assemble or not to resemble - benchmarking of metatranscriptomic practices and a validated Comparative Metatranscriptomics Workflow (CoMW)" describes the comparison of workflows for metatranscriptomics (metaT) analysis based on assembly-free and assembly-based strategies to evaluate precision and recall of functional assignments and expression. The authors use a previously published simulated dataset as well as two real-world metaT datasets from two environments. The main result is that the assembly-based strategy is resulting in less false positives and more precise identification and quantification of active genes. Additionally, an optimized metaT analysis workflow (CoMW) is presented.
In recent years more and more microbiome studies are coming out using metaT instead of metagenomics (metaG) to describe active genes and species within microbial communities. Therefore, it is right in time to benchmark different methods and analysis pipelines for metaT studies. The idea to use the functional assignment and expression as ground truth is a very interesting one. Also standardized, reproducible and optimized pipelines and workflows are important in the still young and moving microbiome field. The main problem that I have with the paper is that it is actually two papers, one paper on testing different strategies for metaT analysis and one paper on a new analysis workflow for metaT analysis. The benchmarking part has the weakness that it is not really a comparison of existing tools or pipelines but of two strategies. The authors defined for each strategy the workflow they considered as the best, but obviously each strategy could be implemented with different tools and steps, and e.g. for the assembly-based strategy different tools and preprocessing steps can have a major impact for the resulting assemblies and therefore for all downstream analysis, but this is not really discussed here. For the second part, the CoMW pipeline, a more detailed description would be good, especially since it contains steps that are not necessary for the benchmark part before, e.g. the noncoding analysis. In general, I would strongly recommend to focus only on one of both parts and restructure the paper accordingly or have two separate papers.
Major revisions:
* in the introduction examples from human gut studies are missing
* a critical review of prior benchmarks in metaG and/or metaT should be given, e.g. CAMI should be mentioned (https://www.ncbi.nlm.nih.gov/pubmed/28967888) and the authors should discuss if the results for metaG and metaT can be compared and what are the specific problems of benchmarking metaT workflows
* for each strategy specific tools and parameters have been used. It should be clearer be stated why they have chosen, e.g. there are newer metaT assemblers than trinity, e.g. rnaspades and megahit, for

the proposed benchmark I would at least want to see two or more implementations for each strategy.
* why those three databases have been chosen, why not others
* no discussion of other workflows available like anvi'o or IMP that could do similar things
* the two real world datasets are only referenced as 'under review', either they should be submitted as preprint or added as supplement to the reviewers. Also, it is not clear if the authors are part of those studies.
* For the simulated dataset it is not totally clear how it was generated. Did the authors use only full-length genes or did they also include the non-coding parts of fully transcribed operon sequences as you would find expect them in real metaT datasets. This will have a big impact on the assembly quality, mapping and expression analysis.
* how are the subsystems that were removed during the benchmarking procedure were chosen
* how the optimal parameters were chosen for alignment and mapping, e.g. BTS
* The software is not straightforward to install, especially for non-bioinformaticians a real reproducible version using docker or conda would be highly appreciated. Also implementing the workflow as set of scripts should be transformed into a more formal structure using a workflow description like snakemake
* Title is way too long and should be changed according the possible restructuring of the manuscript.
Minor revisions:
* line 193: wrong figure seems to be referenced. Figure 3 should be Figure 2
* in paragraph 2.1.3 the reference to Figure 3 (not 6) is missing. Figures 3 and 4: colors not explained and unclear.
* numbers zero to nine should be given as words
* 132: Assembly-based should be lower case
* Table 2: not clear when lower and upper case is used and why
* 161: Nitrogen upper case
* SARTOOLs has a different reference https://www.ncbi.nlm.nih.gov/pmc/articles/PMC4900645/
* ref 21, reference style is wrong
* not clear how to handle references that are currently under review, should be put on bioarxiv e.g. reference 17 and 18
* description of required software versions is missing, e.g. which Java and Python version

Level of Interest

Please indicate how interesting you found the manuscript: Choose an item.

Quality of Written English

Please indicate the quality of language in the manuscript: Choose an item.

Declaration of Competing Interests

Please complete a declaration of competing interests, considering the following questions:

- Have you in the past five years received reimbursements, fees, funding, or salary from an organisation that may in any way gain or lose financially from the publication of this manuscript, either now or in the future?
- Do you hold any stocks or shares in an organisation that may in any way gain or lose financially from the publication of this manuscript, either now or in the future?
- Do you hold or are you currently applying for any patents relating to the content of the manuscript?
- Have you received reimbursements, fees, funding, or salary from an organization that holds or has applied for patents relating to the content of the manuscript?
- Do you have any other financial competing interests?
- Do you have any non-financial competing interests in relation to this paper?

If you can answer no to all of the above, write 'I declare that I have no competing interests' below. If your reply is yes to any, please give details below.

I declare that I have no competing interests.

I agree to the open peer review policy of the journal. I understand that my name will be included on my report to the authors and, if the manuscript is accepted for publication, my named report including any attachments I upload will be posted on the website along with the authors' responses. I agree for my report to be made available under an Open Access Creative Commons CC-BY license (http://creativecommons.org/licenses/by/4.0/). I understand that any comments which I do not wish to be included in my named report can be included as confidential comments to the editors, which will not be published.

I agree to the open peer review policy of the journal

To further support our reviewers, we have joined with Publons, where you can gain additional credit to further highlight your hard work (see: https://publons.com/journal/530/gigascience). On publication of this paper, your review will be automatically added to Publons, you can then choose whether or not to claim your Publons credit. I understand this statement.

Yes Choose an item.
