# Supplementary material for: To assemble or not to resemble—A validated Comparative Metatranscriptomics Workflow (CoMW)
Source: Gigascience. 2019 Jul 30;8(8):giz096. doi: 10.1093/gigascience/giz096 (PMC6667343; doi:10.1093/gigascience/giz096)
Supplement: giz096_Reviewer_2_Report_Original_Submission — Harriet Alexander -- 2/24/2019 Reviewed [file giz096_reviewer_2_report_original_submission.pdf]

Reviewer Report

Title: To assemble or not to resemble â€“ A validated Comparative Metatranscriptomics Workflow (CoMW)

Version: Original Submission **Date:** 2/24/2019

Reviewer name: Harriet Alexander

Reviewer Comments to Author:

Summary:
The manuscript by Anwar et al. presents a novel Metatranscriptomics workflow (CoMW) and runs a comparison of assembly-free and assembly-based methods of metatranscriptome analysis using a simulated bacterial metatranscriptome. The comparison suggests that in most cases assembly-based methods provide results with higher precision and better recall. The authors then apply their novel CoMW workflow (and the assembly-free approach) to the analysis of two different "real" metatranscriptome studies and show major differences in results between the two approaches.
The analysis covered by this paper is very interesting-but the paper is confusingly written and outlined. Important information (such as parameters used for the running of various tools) is difficult to find in the paper or absent. With some reorganization and more detailed methods this paper will be a great contribution to the field.
Major comments:
My primary concern with the paper is that as it stands I am not sure that anyone would be able to replicate these analyses. Broadly, I think that the methods section is confusingly structured. I found myself skipping back and forth to try to figure out what you had done. The methods section should be restructured and organized to follow the flow of the analysis presented in the results. I suggest starting with a section describing how the mock data sets were created, then describe the use of the real world meta-transcriptomes (which aren't well covered in the materials and methods), and then describe the two pipelines that were used including your novel CoMW. Throughout the materials and methods you should make sure that sufficient detail on all programs/tools used are included - meaning that the version of all software products should be included (e.g. DeSeq, Trinity, Diamond, etc.). Additionally, all parameters used should be included. Notably the description of the removal of "functional subsystems" was missing from the methods. How was this done? I also do not think sufficient information was provided regarding how the mock RNA libraries were generated. Moreover, the authors should clarify how the differential expression analysis was done-it appears to be absent from the paper. More detailed comments are provided below.
Broadly speaking, I think it would be useful to have another github repo or a folder on the current github that contains all the supplementary scripts that were used for this publication (e.g. the Polyester scripts used, DE expression, etc.)
The pipeline is great as written-- but I do wonder why it is written in python and not implemented in some sort of workflow language/system (e.g pydoit, snakemake, etc.). If the goal is reproducibility, conda environments (or the like) should be included to facilitate the use of the same software versions

throughout the workflow. Additionally, it would appear that the current scripts within CoMW are written to work with specific versions of each of the software packages covered (e.g. Trinity). What version were these scripts tested with? I tried running and ran into issues with Trinity that I think were due to the version I was trying to use. I suggest making a conda environment file (https://conda.io/projects/conda/en/latest/user-guide/tasks/manage-environments.html) that can be included in the Github repo to detail what version of each of the software programs should be used for the pipeline.

I am perhaps missing it-- but it would appear that no quality trimming of the raw short reads was used in this study. This is generally accepted as best practice. Please explain its omission from both pipelines in the text. More broadly, I think it should be added to the CoMW workflow.

The comparisons made in the paper focus primarily upon functional analysis (e.g. the identification of functional gene sets), and the simulated data set that is used is not well-suited to taxonomic data. The comparisons made in the paper are interesting and valid-- but I think that the authors should make clear that they are only really testing functional annotation and not the resolution of taxonomic annotation.

Looking at Table 1, I am inclined to say that eggNOG does pretty well in general with or without assembly and that the other databases perform less well. Moreover, that other databases seem to be more depend upon assembly vs. not. I am not sure that this shows a clear-cut win for assembly.

Additionally, how much variation might be driven by mapping approach used (Diamond) as compared to some other short read mapper/blasting.

Minor comments:

One approach used frequently in the analysis of microbial communities dominated by prokaryotes is to sequence both the metaG and metaT of the community, assemble the metaG, and then map the metaT to the assembled metaG or binned genomes from the metaG (e.g. https://www.nature.com/articles/s41396-017-0041-5). It seems worthwhile to discuss how your approach differs from these approaches.

Minor comments:

Line 69: Without a meta-analysis I am not sure that this can be stated-citation 11 only lists 5 studies that take that approach in a fairly limited subset of studies. I would remove this statement unless you have solid numbers on how many studies use assembly vs mapping approaches? Also, you should qualify your extrapolation that it is "because of less computational expense." You don't know people's rationale for choosing one pipeline over another.

Line 70: Some discussion of the CAMI metagenomic comparisons (https://data.cami-challenge.org/) seems justified here. They did extensive comparisons of assembly approaches as well as direct taxonomic analysis of short reads. The CAMI parallels your investigation of functional gene annotation nicely.

Line 99: It would be helpful to the reader to directly state what the 'full-length genes' are. I am assuming that they are from the genome data that were used to generate the simulated data. The language should again be clarified in Line 110. One line 110 I am not sure what "full length genes" are being referred to. Perhaps changing the term used to "gold standard" or the like would help the reader follow the logical progression better?

Line 112: Define BTS score.

Line 116: What numerical cutoffs were used for each of the Low, Medium, and High thresholds? Are the

parameters detailed somewhere? I looked at the supplemental tables-but couldn't figure out which if any columns contained these values. Apologies if I missed them.
Line 132: Watch capitalization of your two approaches throughout the paper ("Assembly-based and assembly-free approaches"). You change around quite a bit whether or not you capitalize.
Line 152: As with line 116, what were the parameters used in the 15 "various confidence thresholds". I think it would be helpful to add a supplemental table that specifies the confidence thresholds used.
Line 178: It is unclear to me how "full length" gene alignments were used for quantification? Was this based on mapping the simulated short reads against the original full length genes that were annotated with eggNOG? Clarify in the methods or here.
Line 204: What approach was used by Schostag? Was it assembly free? What was found by the assembly free approach?
Line 209: There is no Figure 6.
Line 220: There is no Figure 7.
Line 243: What was the estimated chimeric sequence frequency from your analysis of the mock datasets? What about false contigs? I would be surprised if they were terribly high as the mock data set didn't contain any species that had high average nucleotide identity.
Line 282: Sure, I agree but do you have numbers to support this or a citation?
Line 283: What are "these pipelines"? Metatranscriptomic pipelines? Also, I am not sure if you have the data to say that the majority of analyses use assembly-free approaches.
Line 324: Can you provide the parameters used in the various steps of the Metatrans pipeline? Also, do you have the scripts used to run the Metatrans pipeline? If so, they should be published somewhere (Supplemental text or GitHub repo for the paper).
Line 336: Please provide the scripts used to generate the short reads.
Line 341: Please clarify what "resulted in a count table and short reads with 2395 genes to add the impact of sequencing coverage" means. It is unclear to me what was produced and why only 2395 genes were recovered if 4943 were generated.
Lines 342: What does knocking out genes mean in the context of short reads? How was this done? Please publish the scripts used-- I couldn't find them in the github repository.
Line 353: Include the accession number for your simulated data set in the text as well as at the end of the paper.
Figure 1: You have "custom python scripts added in CoMW" at the bottom-but I can't see anything highlighted with that in the figure. It might be obscured because the figure is low resolution.
Figure 2: Figure 2 is never referenced in the text. Also, I can't read this figure well-the text is very small and the resolution is not good. Generally, instead of plotting Log2Fold change I wonder if it would be more powerful to plot the difference relative to the Full length gene analysis.
Table 2: All tables should be able to stand alone with their caption without the text of the paper. This table is lacking some key information. What is the percentage column? What are the numbers (the number of orthologs retrieved with each method?) Label the letters-- specify what they are/where they are from. Check consistency in capitalization in titles. Specify that this is for eggNog? I also wonder if it would be better to report Recall/Precision values for the two different approaches as you did in Table 1- as it is a bit strange to recover more values in the assembly-based approach compared to the full length genes but then not penalize what should be false discover.

Figures 3 and 4: The figure legends require more information: such as what is being plotted-relative abundance calculated how? Relative to all transcripts or relative to the study? It might also be interesting to add the relative abundance values that are reported in the original papers. These figures are interesting as the dynamic range of the data recovered is so different between the two different approaches. What do you think is the origin of this difference? Lack of mapping in the assembly free? Or spurious mapping of reads in the assembly free? It might be nice to add some more discussion of this in the text.

Level of Interest

Please indicate how interesting you found the manuscript: Choose an item.

Quality of Written English

Please indicate the quality of language in the manuscript: Choose an item.

Declaration of Competing Interests

Please complete a declaration of competing interests, considering the following questions:

- Have you in the past five years received reimbursements, fees, funding, or salary from an organisation that may in any way gain or lose financially from the publication of this manuscript, either now or in the future?
- Do you hold any stocks or shares in an organisation that may in any way gain or lose financially from the publication of this manuscript, either now or in the future?
- Do you hold or are you currently applying for any patents relating to the content of the manuscript?
- Have you received reimbursements, fees, funding, or salary from an organization that holds or has applied for patents relating to the content of the manuscript?
- Do you have any other financial competing interests?
- Do you have any non-financial competing interests in relation to this paper?

If you can answer no to all of the above, write 'I declare that I have no competing interests' below. If your reply is yes to any, please give details below.

I declare that I have no competing interests.

I agree to the open peer review policy of the journal. I understand that my name will be included on my report to the authors and, if the manuscript is accepted for publication, my named report including any attachments I upload will be posted on the website along with the authors' responses. I agree for my report to be made available under an Open Access Creative Commons CC-BY license (http://creativecommons.org/licenses/by/4.0/). I understand that any comments which I do not wish to

be included in my named report can be included as confidential comments to the editors, which will not be published.

I agree to the open peer review policy of the journal

To further support our reviewers, we have joined with Publons, where you can gain additional credit to further highlight your hard work (see: https://publons.com/journal/530/gigascience). On publication of this paper, your review will be automatically added to Publons, you can then choose whether or not to claim your Publons credit. I understand this statement.

Yes Choose an item.
